# Inter‐ and intraspecific variation in mycotoxin tolerance: A study of four *Drosophila* species

**DOI:** 10.1002/ece3.9126

**Published:** 2022-07-24

**Authors:** Prajakta P. Kokate, Morgan Smith, Lucinda Hall, Kui Zhang, Thomas Werner

**Affiliations:** ^1^ Department of Biological Sciences Michigan Technological University Houghton Michigan USA; ^2^ Department of Mathematical Sciences Michigan Technological University Houghton Michigan USA

**Keywords:** geographical variation, interspecific genetic variation, intraspecific genetic variation, mycotoxin tolerance

## Abstract

Many mycophagous *Drosophila* species have adapted to tolerate high concentrations of mycotoxins, an ability not reported in any other eukaryotes. Although an association between mycophagy and mycotoxin tolerance has been established in many *Drosophila* species, the genetic mechanisms of the tolerance are unknown. This study presents the inter‐ and intraspecific variation in the mycotoxin tolerance trait. We studied the mycotoxin tolerance in four *Drosophila* species from four separate clades within the *immigrans*‐*tripunctata* radiation from two distinct locations. The effect of mycotoxin treatment on 20 isofemale lines per species was studied using seven gross phenotypes: survival to pupation, survival to eclosion, development time to pupation and eclosion, thorax length, fecundity, and longevity. We observed interspecific variation among four species, with *D. falleni* being the most tolerant, followed by *D. recens*, *D. neotestacea*, and *D. tripunctata*, in that order. The results also revealed geographical variation and intraspecific genetic variation in mycotoxin tolerance. This report provides the foundation for further delineating the genetic mechanisms of the mycotoxin tolerance trait.

## Graphical abstract

Many mycophagous Drosophila species have adapted to tolerate high concentrations of mycotoxins, an ability not reported in any other eukaryotes. We observed interspecific variation among four species, with D. falleni being the most tolerant, followed by D. recens, D. neotestacea, and D. tripunctata, in that order. We further observed fitness effects of the mycotoxin tolerance in two species: D. tripunctata and D. neotestacea.

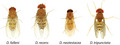

## 1 INTRODUCTION

Many species within the genus *Drosophila* have radiated to use a wide variety of hosts for feeding and breeding (Markow, 2019). These hosts are chemically and phenologically distinct and include fruits, flowers, cacti, slime fluxes, and mushrooms. These adaptations involve genetic and genomic changes. One such adaptive radiation is mycophagy. Many *Drosophila* species are mycophagous, and mushrooms appear to provide all the essential components of an insect diet (Courtney et al., 1990). However, some mushroom species also contain highly lethal compounds (mycotoxins) to protect themselves from mycophagy (Stump et al., 2011). Although the toxic mushroom species are fewer in number as compared to the nontoxic mushroom species (Graeme, 2014) and only constitute a small portion of the potential diet, many mycophagous *Drosophila* species can tolerate high concentrations of α‐amanitin, which is the most potent mycotoxin (Jaenike et al., 1983; Lacy, 1984; Spicer & Jaenike, 1996; Stump et al., 2011).

Seventeen mycophagous *Drosophila* species from five species groups within the *immigrans*‐*tripunctata* radiation have been shown to tolerate mycotoxins (Izumitani et al., 2016; Scott Chialvo & Werner, 2018). These species groups are *tripunctata*, *testacea*, *cardini*, *bizonata* and *quinaria*. Very little is known about the feeding habits for the species groups *cardini* and *bizonata*. The *tripunctata* species group comprises 83 species (O'Grady & DeSalle, 2018), and for most species in this group, larval feeding substrates have not yet been determined. The *testacea* species group contains four species, all of which are mycophagous, whereas 34 species belong to the *quinaria* group, most of which are mycophagous (O'Grady & DeSalle, 2018; Scott Chialvo et al., 2019). The *quinaria* species group is of particular interest as mycophagy has been gained and lost multiple times within this group. Furthermore, the loss of mycophagy has been followed by a loss of toxin tolerance without an evolutionary lag (Spicer & Jaenike, 1996), suggesting that mycotoxin tolerance is probably a costly trait.

Although the association between mycophagy and mycotoxin tolerance in certain *Drosophila* species was established almost three decades ago, the genetic mechanisms involved in the tolerance are mainly unknown. The most lethal mycotoxin, found in the notoriously deadly *Amanita* mushrooms, alpha‐amanitin, binds to RNA‐polymerase II (RNA‐pol II) and hinders its function. Jaenike et al. (1983) observed that the tolerance mechanism apparently did not involve target modification of RNA‐pol II. Another study demonstrated that Phase I detoxification enzymes (Cytochrome P450s) might be conferring mycotoxin tolerance in some but not all mycophagous species (Stump et al., 2011). Apart from these few reports, the understanding of the genetic basis of mycotoxin tolerance has remained inadequate.

To identify mechanisms that confer mycotoxin tolerance, we must understand the genetic architecture of the trait. To achieve this goal, we consider the following questions: (1) Does the mycotoxin tolerance trait show intraspecific genetic variation? (2) Do different species demonstrate variation in the extent of mycotoxin tolerance?

**FIGURE 1:**
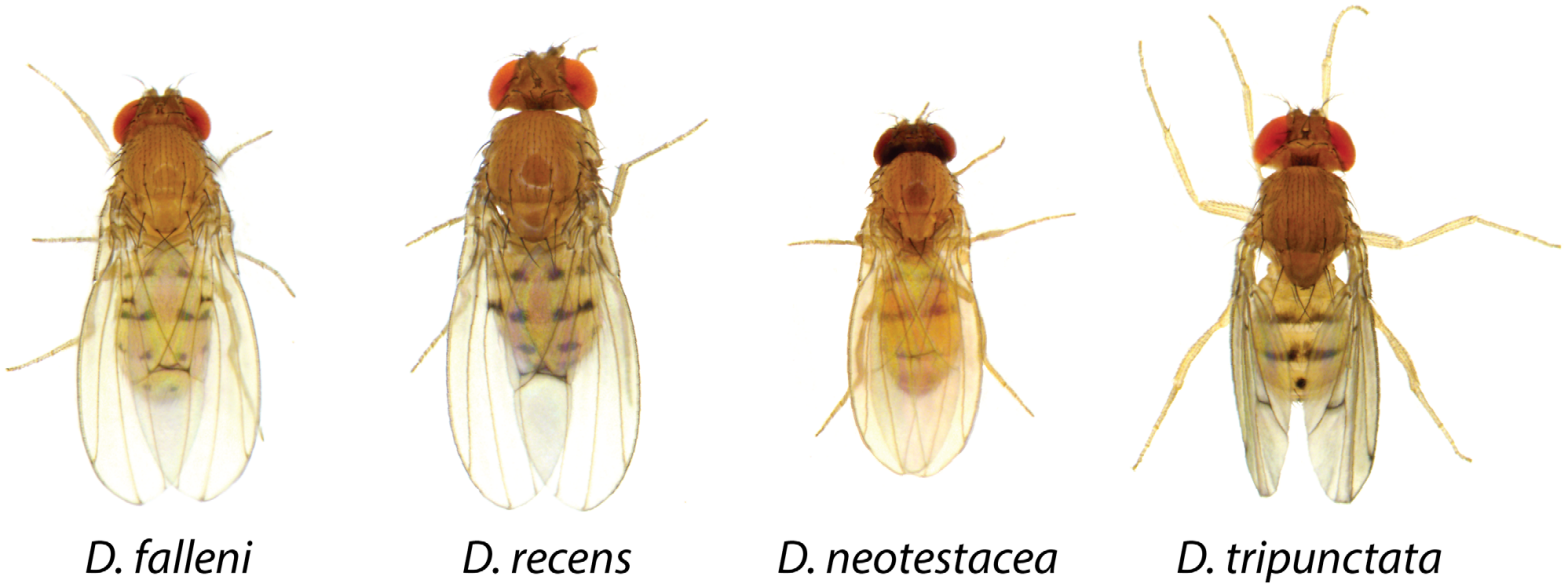
Representative images of the four organisms used in this study

**FIGURE 2:**
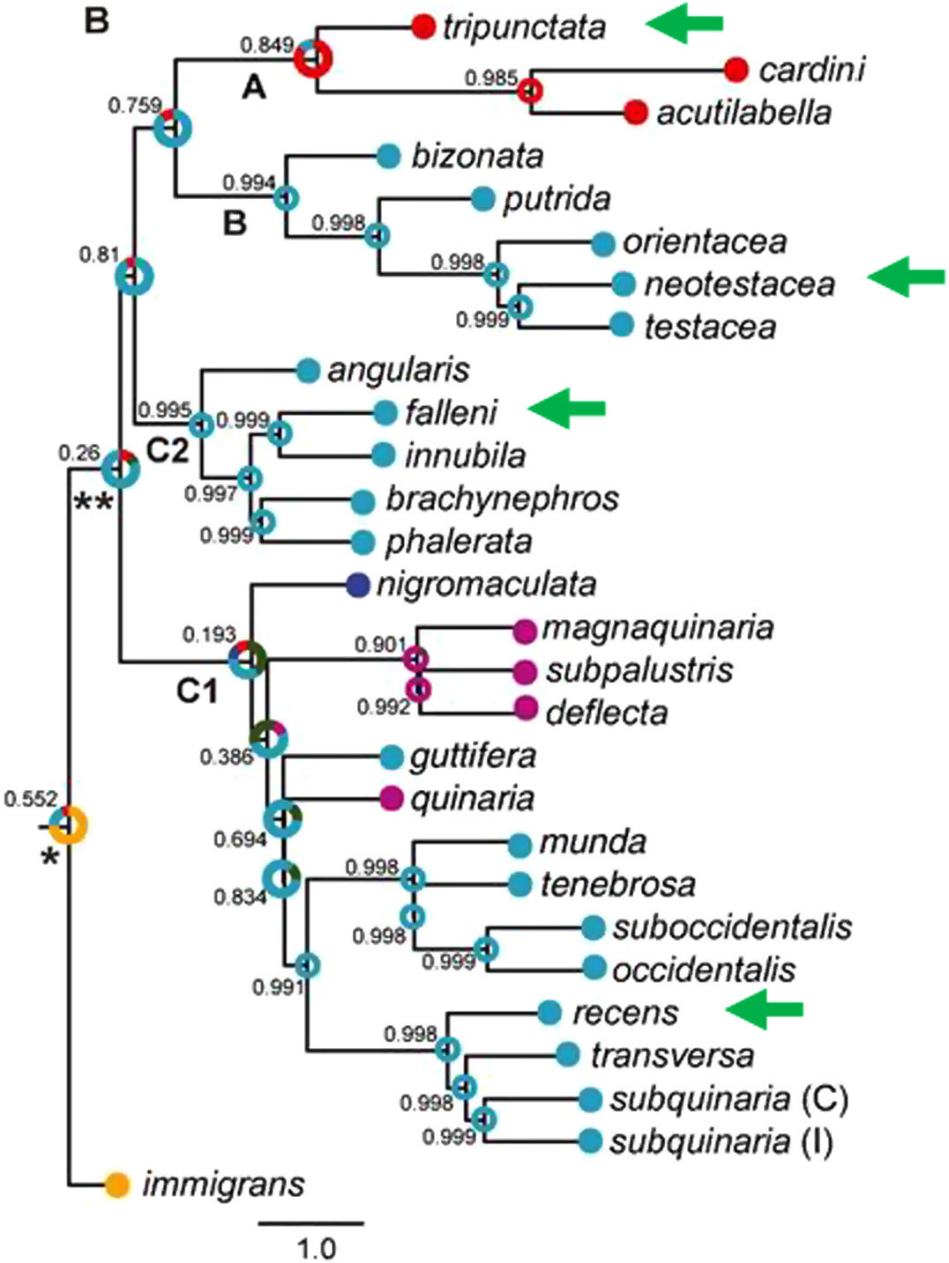
Phylogenetic tree showing different clades within the immigrans‐tripunctata radiation. The four species, one from each clade, are shown with green arrows. Image reproduced from Scott Chialvo et al. (2019), with copyright permission from the journal

To address these questions, we have performed mycotoxin tolerance assays on multiple isofemale lines of four species within the *immigrans*‐*tripunctata* radiation. Figure 1 provides images of the four species: *D. falleni*, *D. recens*, *D. neotestacea*, and *D. tripunctata* (Werner et al., 2020). Figure 2 shows each species in the phylogenetic context. Drosophila *tripunctata* belongs to the *tripunctata* species group (Clade A), *D. neotestecea* belongs to the *testacea* species group (Clade B), *D. recens* and *D. falleni* belong to the quinaria species group, Clade C1, and Clade C2, respectively. Each of these four species represents a major clade of the *immigrans*‐*tripunctata* radiation and is known to be mycotoxin tolerant.

Previous research on mycotoxin tolerance in *Drosophila* has focused on α‐amanitin, the toxin found at high concentrations in some *Amanita* mushroom species (Garcia et al., 2015; Jaenike et al., 1983; Jaenike, 1985; Lacy, 1984; Spicer & Jaenike, 1996; Tuno et al., 2007; Stump et al., 2011). However, toxic mushrooms contain a myriad of different toxins (Yin et al., 2019). Therefore, studies based on a single toxin in isolation have a drawback. They cannot account for the potential synergistic and antagonistic interactions among different mycotoxins found in wild toxic mushrooms. In this study, we have used a natural‐toxin mix (Scott Chialvo et al., 2020) as a source of mycotoxins. First, using this natural‐toxin mix extracted from *Amanita phalloides* mushrooms ensures a mycotoxin representation similar to that found in the wild, as it contains α‐amanitin, β‐amanitin, ɤ‐amanitin, amanin, amanullin, phallacidin, phallisacin, phalloin, phallisin, and phalloidin (Scott Chialvo et al., 2020). The second reason is that α‐amanitin is expensive, and therefore, the use of the natural‐toxin mix proved to be cost‐effective for this large‐scale study.

## 2 MATERIALS AND METHODS

### 2.1 Fly isofemale lines

Four species were included in this study: *D. falleni*, *D. recens*, *D. neotestacea*, *and D. tripunctata*. Adult flies were collected by net sweeping on fermented banana baits, tomato baits, and mushroom baits over the summer months of 2017–2019 from two distant locations: Great Smoky Mountain National Park near Gatlinburg, TN (hereafter referred to as GSM) and Little Bay de Noc in Escanaba in The Upper Peninsula of Michigan (hereafter referred to as ESC). These two sites are approximately 1400 km apart. Multiple sites were used for fly collection within each location spanning over 3–5 square kilometers. The species and the sex of the captured flies were identified, and isofemale lines were set up by adding one wild‐caught female with one wild‐caught male from the same species and location and collecting their progeny (David et al., 2005). The established isofemale lines were maintained on a diet of Carolina Biological Formula 4–24 Instant Drosophila Medium supplemented with finely ground, freeze‐dried *Agaricus bisporus* mushrooms (Oregon mushrooms, OR) at a ratio of 33.28:1 w/w, and a dental roll was added to the food vial as a pupation site. The standard conditions for maintenance and experiments were 22°C and a 14 h:10 h (L:D) photoperiod at 60% humidity. The authors note here that the isofemale lines were maintained in the laboratory for at least over a year before the experiments were conducted.

### 2.2 Mycotoxin tolerance assays

Basic food was prepared by mixing 28.3 g freeze‐dried *A. bisporus* mushrooms (Oregon mushrooms, Oregon) with 941.9 g Carolina 4–24 Instant Drosophila Medium and grinding them together into a fine powder. For mycotoxin tolerance assays, clean glass vials were filled with 250 mg of basic food.

The natural‐toxin mix was provided by Dr. Clare Scott‐Chialvo (Scott Chialvo et al., 2020), which contained methanol as eluent. To account for this methanol, one milliliter of 0.56% methanol solution was added to the control vials containing 250 mg of basic food. The mycotoxin vials were prepared by adding 1 ml of the natural‐toxin mix (100 μg/ml of known amatoxins) to the vials containing 250 mg of basic food. Both control and mycotoxin vials were weighed and subjected to vacuum evaporation for 96 h at room temperature to remove methanol from the vials. The loss in weight (in grams) in the vials was replenished with the appropriate amount (in ml) of sterile distilled water. The optimal duration of vacuum evaporation was identified using preliminary studies and 96 h of vacuum evaporation showed survivorship that was comparable to vials without methanol.

Water agar plates were prepared using 15 g Bacto Agar (Sigma Aldrich) in 500 ml of distilled water and adding Tegosept to a final concentration of 0.1% and poured into 30 mm Petri‐plates. These plates snugly fit the plastic bottles that were used to make egg‐laying chambers. Tiny holes were punched into these plastic bottles for aeration. Equal amounts of dry yeast and freeze‐dried mushroom powder were mixed together with autoclaved distilled water to prepare a paste (prepared fresh daily). A drop of this paste was applied to the water agar plate. Recently eclosed males and females of each isofemale line were transferred to egg‐lay chambers and allowed to oviposit at 22°C and a 14 h:10 h (L:D) photoperiod at 60% humidity. The next day, the plates were replaced with fresh plates, and the water agar plates with oviposited eggs were allowed to hatch at 22°C and a 14 h:10 h (L:D) photoperiod at 60% humidity. The hatched first‐instar larvae were used for the experiments. Pilot studies were performed to identify the optimal larval density for each species. As a result, 15 first‐instar larvae were added to each vial in the case of *D. falleni*, *D. recens*, and *D. tripunctata*, whereas 20 first‐instar larvae were added to each vial for *D. neotestacea*. The experiments were conducted in triplicates. Each experiment was conducted on consecutive days to generate three replicates for each of the 10 isofemale lines/location/species for two treatments (control and mycotoxin).

#### 2.2.1 Development time, thorax length measurements, and survival

The vials were checked daily to record the time to pupation, survival to pupation, time to eclosion, and survival to eclosion. The eclosed flies were collected within 24 h by light CO_2_ anesthesia, sexed, and placed laterally to measure the thorax length. The thorax's anterior margin length to the scutellum's posterior tip was measured and recorded as the thorax length. The thorax length of the eclosed flies was measured to the nearest 0.025 mm with an Olympus SZX16 dissection microscope fitted with an Olympus DP72 camera, using the ImageScan software (Hasson et al., 1992).

The eclosed females were used for the fecundity assays, and the eclosed males were used for the longevity assays. The experiments were terminated after ensuring that no new flies had emerged for four consecutive days.

#### 2.2.2 Fecundity assays

Only female flies were used for the fecundity assays. Female flies eclosed from the mycotoxin tolerance assay vials were labeled appropriately and maintained individually in food vials for 3 days as virgins. They were then transferred individually into a fresh food vial with three 3‐day‐old virgin males from the laboratory stocks of the same isofemale line. These parent flies were transferred to a new vial every 3 days. After 15 days, the adult flies were removed. The offspring of the females that survived the full 15 days were counted to provide an estimate of fecundity. We note that this assay cannot be used to evaluate egg‐to‐adult survival (Dyer & Hall, 2019).

#### 2.2.3 Longevity assays

Only male flies were used for the longevity study. Male flies eclosed from the mycotoxin tolerance assay vials were maintained individually in tiny 5‐ml glass vials containing approximately 250 mg of the basic food used to create the mycotoxin tolerance assay vials. The vials were checked every alternate day to record any dead flies, and the remaining flies were transferred to fresh food vials every 2–3 days.

### 2.3 Statistical analyses

All statistical analyses were done using R version 3.6.1 (https://www.r‐project.org/foundation/) and R Studio version 2021.09.2 + 382 (https://www.rstudio.com/). We used the linear mixed model (LMM) and the generalized linear‐mixed effects model (GLMM), implemented in R package ‘lme4’ (Bates et al., 2015), to determine the independent variables that can explain the variation in survival, development time, body size, fecundity, and longevity. We modeled pupal and survival to eclosion using a binomial linear mixed model with the logistic link function. We used the linear mixed model to model development time, fecundity, longevity, and thorax lengths. The development time and thorax lengths were analyzed for each sex separately. To detect whether mycotoxin tolerance shows interspecific variation, we first fitted a GLMM that includes the main effects, the two‐way interactions, and the three‐way interaction of the species, the treatment, and the location as the fixed effects. The likelihood test (LRT) was used to test if the three interaction was significant. The final model includes the main effects and the two‐way interactions of the species, the treatment, and the three‐way interaction only if the three‐way interaction is significant (in other words, the *p*‐value from the LRT for the three‐way interaction is less than 0.05). In all models, the isofemale lines and the replicate vials were included as the random effects. To check the sufficiency of the model, the scatter plots of the deviance residuals against the predicted values were generated, and the dispersion parameter was estimated based on the ratio of the sum of squared deviance residuals and the degrees of freedom of the model if the binomial linear mixed model was used.

To evaluate the effect of toxin treatment, we fitted either a binomial linear mixed or linear mixed model to assess whether the treatment affects the seven gross phenotypes. In all models, the main effect of the treatment was the only fixed effect, and the isofemale lines and the replicate vials were included as the random effects. The analysis was conducted for each of four species and seven gross phenotypes separately. Among seven gross phenotypes, the development time of eclosion and the thorax length of eclosion were analyzed for the males and the females separately. Therefore, 36 *p*‐values were obtained. To account for the multiple testing adjustment, both the *p*‐value adjusted using the Bonferroni correction and the false discovery rate (FDR) calculated with the Benjamini–Hochberg method (Benjamini & Hochberg, 1995) were presented. The FDR < 0.05 was used as a cutoff for significant results.

To identify the extent of tolerance for each isofemale line, the binomial linear mixed model was performed on each isofemale line with the replicate vial as a random effect, and the lines were segregated based on their *p*‐values. High‐tolerance lines were identified as those in which no significant difference in survival between the control and the mycotoxin treatments was observed or where the survival was significantly higher in the mycotoxin treatment. Isofemale lines with significantly low survivorship on the mycotoxin treatment (*p*‐value <.05) were categorized as low‐tolerance lines. For the scope of this study, high tolerance is defined as the ability of an isofemale line to survive in the presence of the natural‐toxin mix (100 μg/ml of known amatoxins).

For intraspecific variation, before model fitting, we pruned the data to exclude isofemale lines where only one data point was observed per treatment. This exclusion allowed us to eliminate data that could not estimate variation within an isofemale line. We assessed whether the main effects: (a) isofemale line, (b) treatment (presence or absence of mycotoxin), (c) location, and (d) interactions between the main effects affect the seven gross phenotypes (survival to pupation, survival to eclosion, development time to pupation and eclosion, thorax length, fecundity, and longevity) in each species. The isofemale lines and the replicate vials were included in the analysis as random effects.

## 3 RESULTS

### 3.1 Interspecific variation

**FIGURE 3:**
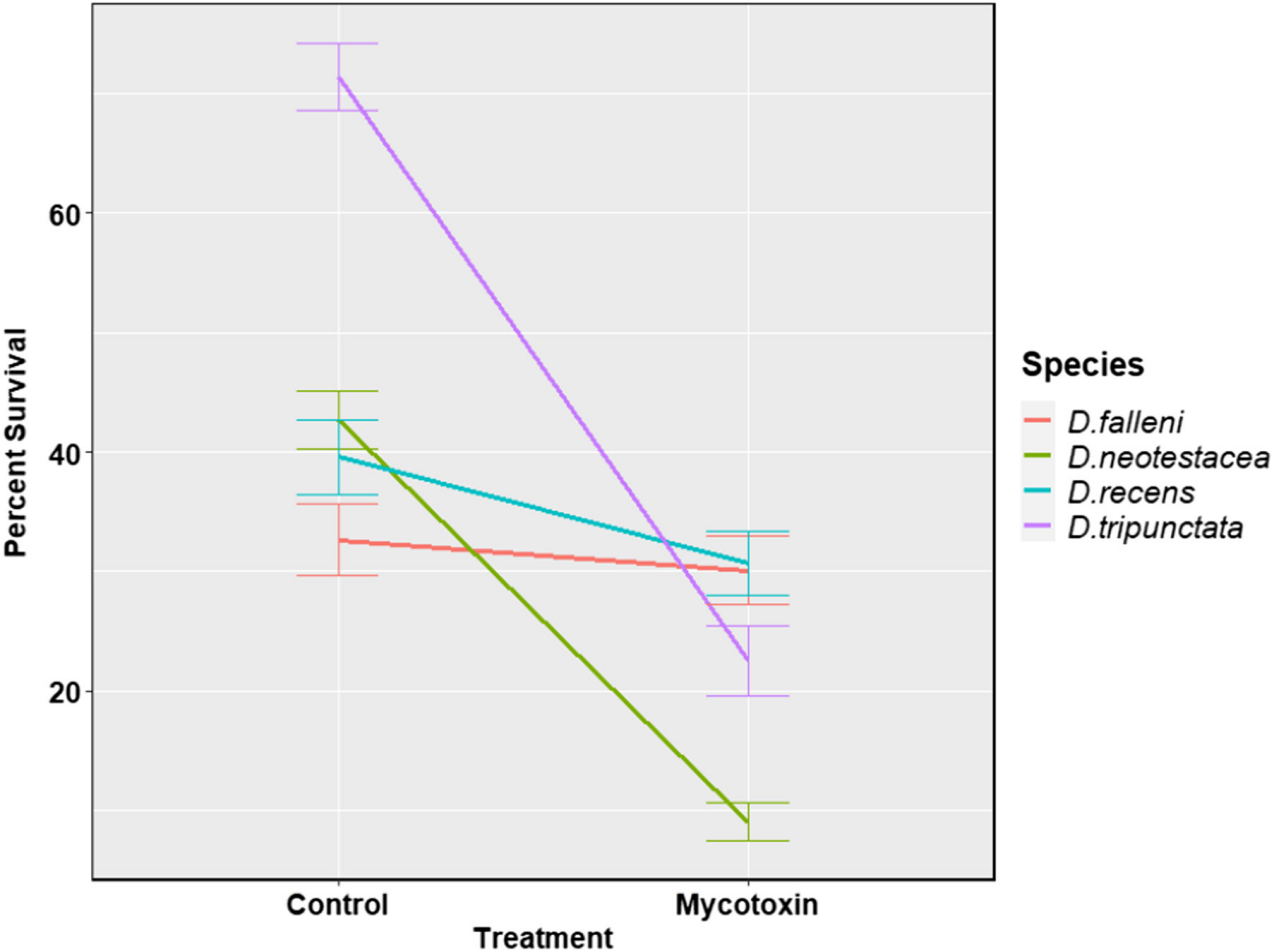
Interspecific variation in mycotoxin tolerance for survival to pupation

**FIGURE 4:**
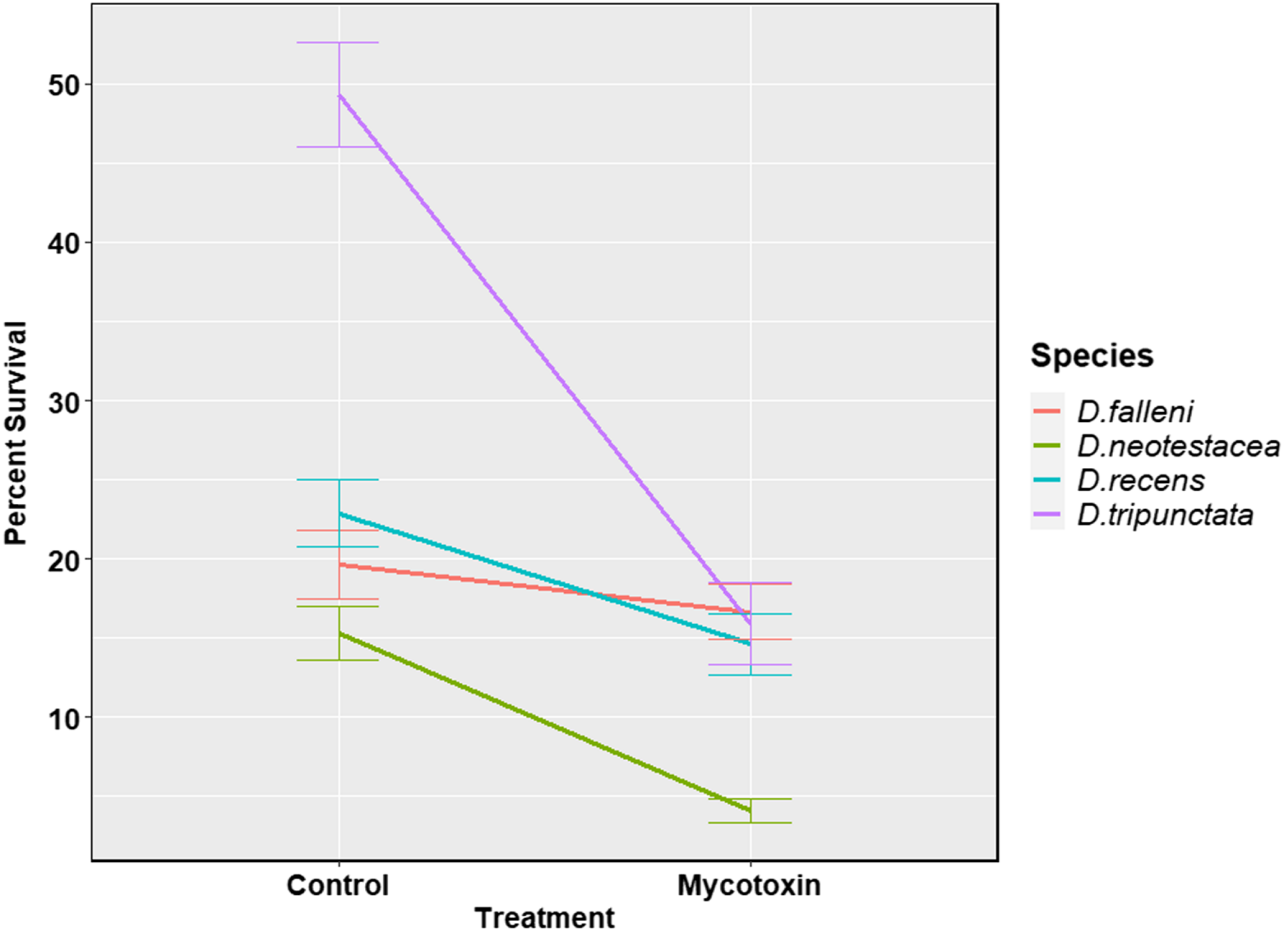
Interspecific variation in mycotoxin tolerance for survival to eclosion

For survival to pupation and survival to eclosion, the *p*‐values for the three‐way interaction were 0.273 and 0.431, respectively. Either of them achieved the significance level of 0.05. Therefore, the models with all the main effects and the two‐way interactions of the species, the treatment, and the location were used. Tables 1 and 2 present the *p*‐values from the LRT for each term in the models for survival to pupation and survival to eclosion, respectively. The least square estimates of estimate and bounds (95% confidence intervals) of the logarithmic of the odds ratio between each pair of species stratified by the treatment are presented in Tables S1 and S2, respectively. We observed a significant interspecific variation in mycotoxin tolerance for survival to pupation (*p*‐value <.001, Table 1) and survival to eclosion (*p*‐value <.001, Table 2) among the four species: *D. falleni*, *D. recens*, *D. neotestacea*, and *D. tripunctata*. As depicted in Figures 3 and 4, pupal and survival to eclosion was unaffected in *D. falleni*, slightly affected in *D. recens*, and significantly reduced in *D. neotestacea* and *D. tripunctata*.

**TABLE 1:** GLMM analysis of interspecific variation in mycotoxin tolerance for survival to pupation

Effect	Chi‐square statistic	Degrees of freedom	p‐value
Species	44.704	1	1.07 × 10^−9^
Treatment	0.007	3	.935
Location	0.000	1	.983
Species:Treatment	320.973	1	2.87 × 10^−69^
Species:Location	7.557	3	0.056
Treatment:Location	4.914	3	.027

**TABLE 2:** GLMM analysis of interspecific variation in mycotoxin tolerance for survival to eclosion

Effect	Chi‐square statistic	Degrees of freedom	p‐value
Species	54.634	1	8.22 × 10^−12^
Treatment	0.1339	3	.714
Location	2.149	1	.143
Species:Treatment	108.281	1	2.57 × 10^−23^
Species:Location	3.561	3	.313
Treatment:Location	5.453	3	.019

**FIGURE 5:**
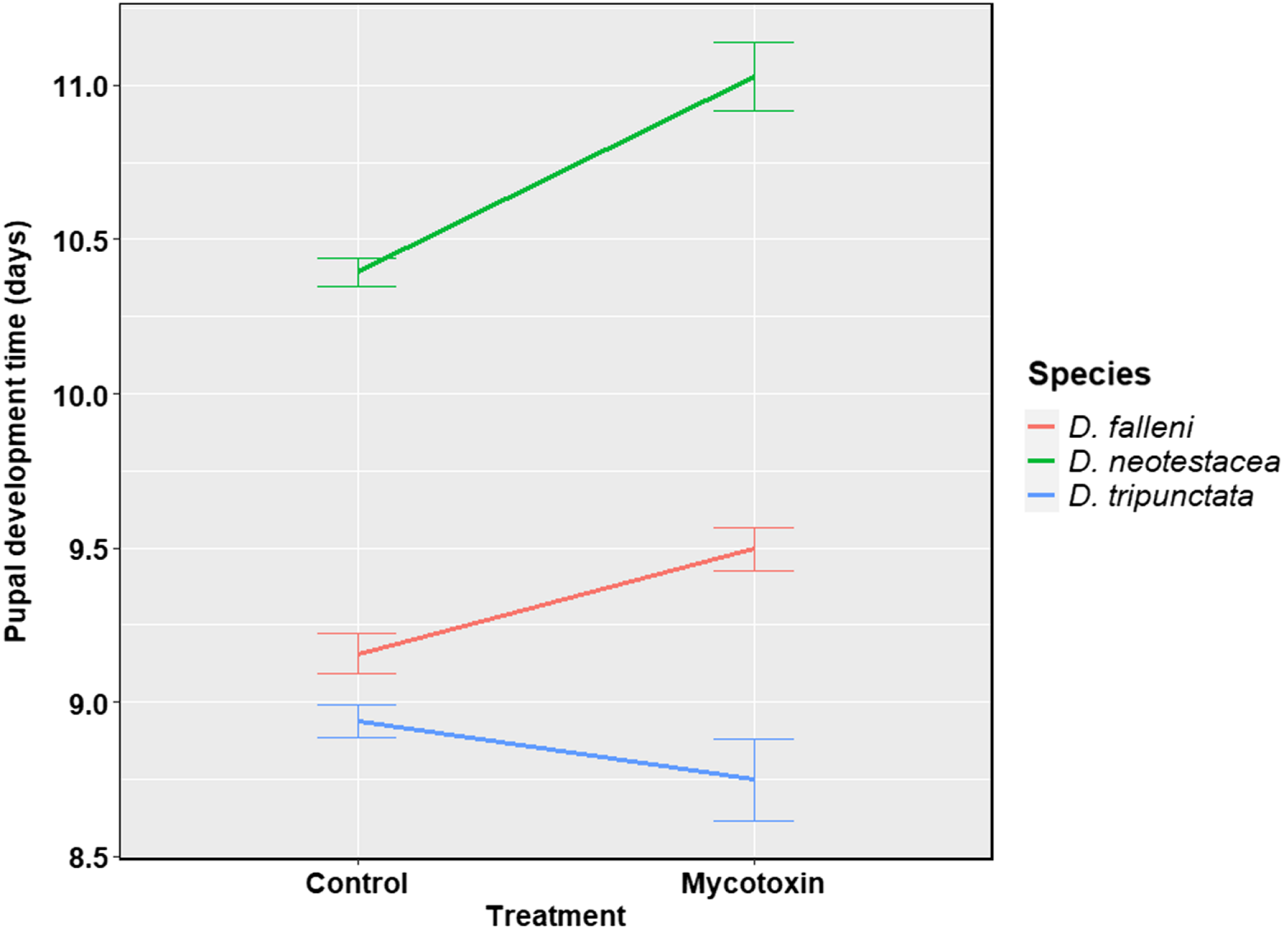
Effect of mycotoxin treatment on pupal development time (days)

**FIGURE 6:**
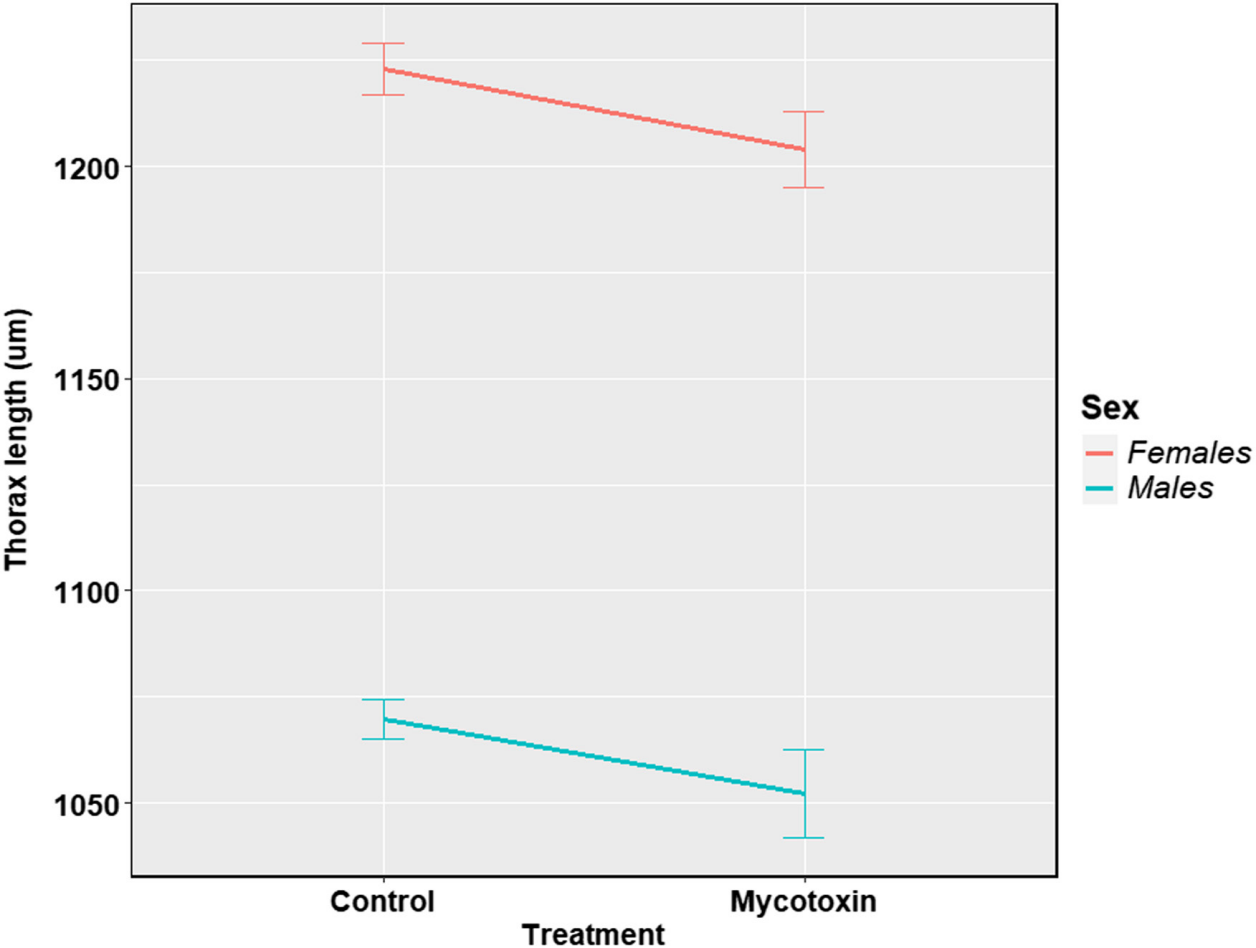
Effect of mycotoxin treatment on thorax lengths of *D. tripunctata* males and females

The significant treatment effects are presented in Table 3. The results for all four species and seven gross phenotypes can be found in Table S3. Interestingly, the effect of mycotoxin treatment followed a similar trend (Table 3). For example, only pupal development time was significantly delayed in isofemale lines of *D*. *falleni* (FDR = 0.001 and the adjusted *p*‐value <.05). Survival to pupation and survival to eclosion were significantly reduced in *D. recens* isofemale lines (all corresponding FDRs and the adjusted *p*‐values <.001). Mycotoxin treatment significantly affected four phenotypes in *D. neotestacea*: survival to pupation (FDR < 0.001 and the adjusted *p*‐value <.001), survival to eclosion (FDR < 0.001 and the adjusted *p*‐value <.001), pupal development time (FDR < 0.001 and the adjusted *p*‐value <.001), and development time of eclosed females (FDR < 0.01 and the adjusted *p*‐value <.05). Mycotoxin tolerance affected six phenotypes in *D. tripunctata*, survival to pupation (FDR < 0.001 and the adjusted *p*‐value <.001), survival to eclosion (FDR < 0.001 and the adjusted *p*‐value <.001), pupal development time (FDR < 0.05 and the adjusted *p*‐value = .624), development time of eclosed males (FDR < 0.01 and the adjusted *p*‐value = .115), and thorax lengths of males (FDR < 0.001 and the adjusted *p*‐value <.05) and females (FDR < 0.01 and the adjusted *p*‐value = .055). It is intriguing that while mycotoxin treatment delayed pupal development in *D. falleni* and *D. neotestacea*, the development time was reduced due to mycotoxin treatment in *D. tripunctata* (Figure 5). Peculiarly, *D. tripunctata* was also the only species out of the four where the mycotoxin treatment reduced the thorax lengths of the eclosed males and females significantly (Figure 6). It would be interesting to know whether *D. tripunctata* displays a trade‐off between pupal development time and body size.

**TABLE 3:** Table depicting traits in all four species showing a significant effect of the mycotoxin treatment

Species	Trait	*p*‐value	Bonferroni adj. *p* value	FDR (BH)	Mean ± SD (*N*)		Estimate (SE)	95% CI
					Control	Mycotoxin		
*D. falleni*	Pupal development time (days)	2.02 × 10^−4^	.007	0.001	9.16 ± 1.11 (289)	9.5 ± 1.12 (264)	−0.305 (0.082)	(−0.47, −0.14)
*D. recens*	Pupal survival (number)	1.20 × 10^−5^	4.33 × 10^−4^	6.19 × 10^−5^	5.94 ± 3.67 (374)	4.63 ± 3.15 (292)	0.458 (0.105)	(0.25, 0.66)
*D. recens*	Fly survival (number)	2.14 × 10^−6^	7.71 × 10^−5^	1.29 × 10^−5^	3.37 ± 2.49 (215)	2.19 ± 2.31 (138)	0.583 (0.123)	(0.34, 0.82)
*D. neotestecea*	Pupal survival (number)	1.09 × 10^−69^	<2.00 × 10^−16^	<2.00 × 10^−16^	8.53 ± 3.75 (512)	1.82 ± 2.43 (109)	2.135 (0.121)	(1.90, 2.37)
*D. neotestecea*	Fly survival (number)	.000	<2.00 × 10^−16^	<2.00 × 10^−16^	3.05 ± 2.61 (183)	0.8 ± 1.13 (48)	1.496 (0.002)	(1.49, 1.50)
*D. neotestecea*	Pupal development time (days)	1.16 × 10^−12^	4.16 × 10^−11^	8.33 × 10^−12^	10.4 ± 1.03 (512)	11 ± 1.16 (109)	−0.625 (0.088)	(−0.80, −0.45)
*D. neotestecea*	Development time of eclosed females (days)	5.00 × 10^−4^	.021	0.002	17.7 ± 1.69 (92)	17.9 ± 1.7 (29)	−1.200 (0.359)	(−1.91, −0.49)
*D. tripunctata*	Pupal survival (number)	1.88 × 10^−87^	<2.00 × 10^−16^	<2.00 × 10^−16^	10.7 ± 3.26 (643)	3.38 ± 3.41 (203)	2.437 (0.123)	(2.20, 2.68)
*D. tripunctata*	Fly survival (number)	1.42 × 10^−51^	<2.00 × 10^−16^	<2.00 × 10^−16^	7.4 ± 3.8 (444)	2.38 ± 3.03 (143)	1.891 (0.125)	(1.65, 2.14)
*D. tripunctata*	Pupal development time (days)	.017	.624	0.048	8.91 ± 1.3 (643)	8.87 ± 1.46 (203)	−0.207 (0.087)	(−0.38, −0.04)
*D. tripunctata*	Thorax length of eclosed males (μm)	.003	.115	0.010	1070 ± 56.5 (146)	1052 ± 64.8 (39)	27.516 (9.452)	(8.86, 46.17)
*D. tripunctata*	Development time of eclosed males (days)	3.62 × 10^−4^	.013	0.001	14.6 ± 1.47 (146)	15 ± 1.45 (39)	−0.591 (0.166)	(−0.92, −0.26)
*D. tripunctata*	Thorax length of eclosed females (μm)	.002	.055	0.005	1223 ± 78.7 (170)	1204 ± 74.6 (71)	33.258 (10.642)	(12.29, 54.23)

The number of high‐tolerance isofemale lines in each species is presented in Table S4. When the mycotoxin tolerance was evaluated for each isofemale line in *D. falleni*, only two isofemale lines of 20 showed reduced survival on mycotoxin treatment. However, contrary to *D. falleni*, most of the isofemale lines of *D. neotestacea* and *D. tripunctata* showed low tolerance (12/20 and 15/20, respectively). *Drosophila recens* had intermediate tolerance with 7/20 isofemale lines showing low tolerance to mycotoxin treatment.

### 3.2 Intraspecific genetic variation

**FIGURE 7:**
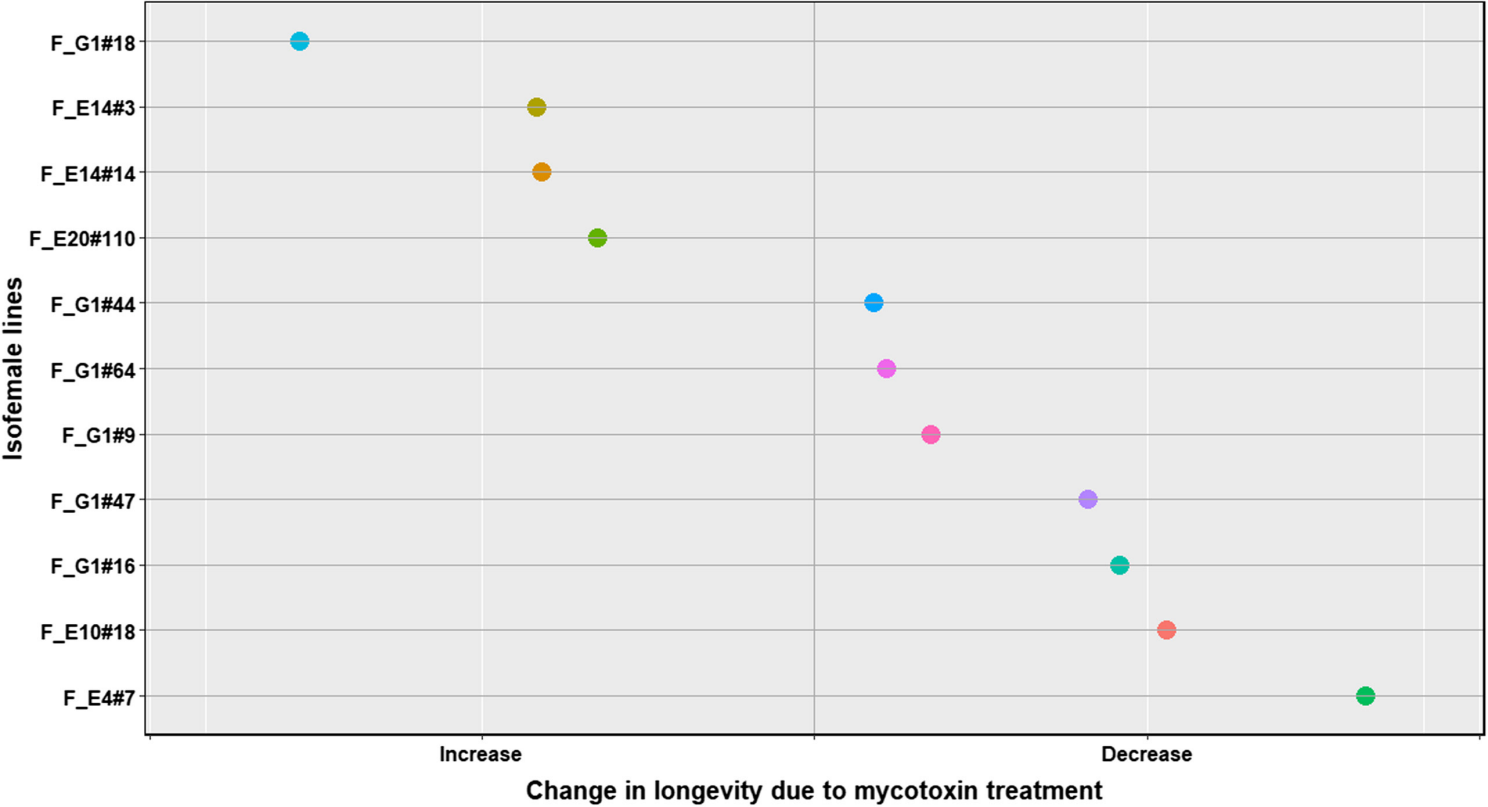
Intraspecific variation in mycotoxin tolerance for longevity in *D. falleni*

**FIGURE 8:**
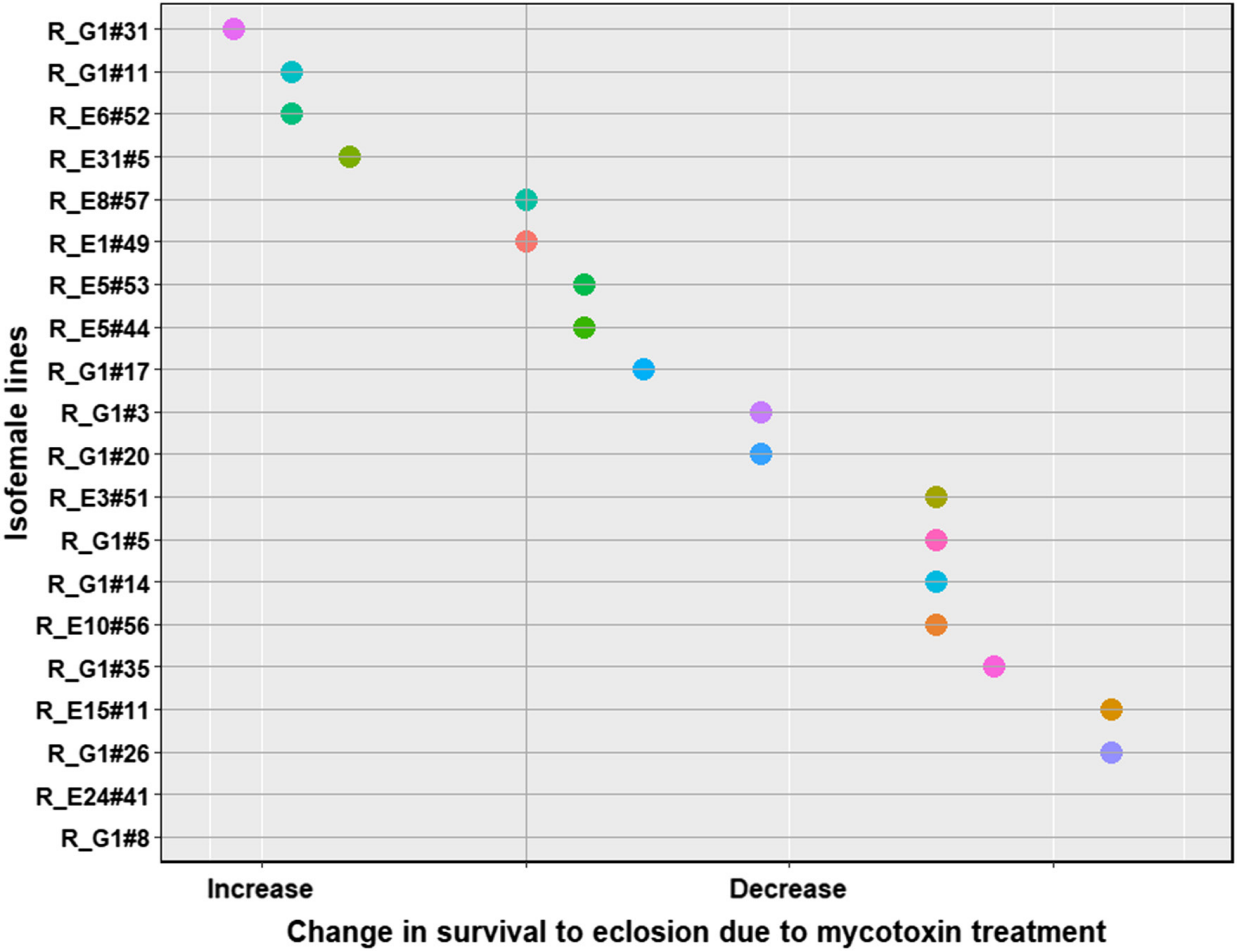
Intraspecific variation in mycotoxin tolerance for survival to eclosion in *D. recens*

**FIGURE 9:**
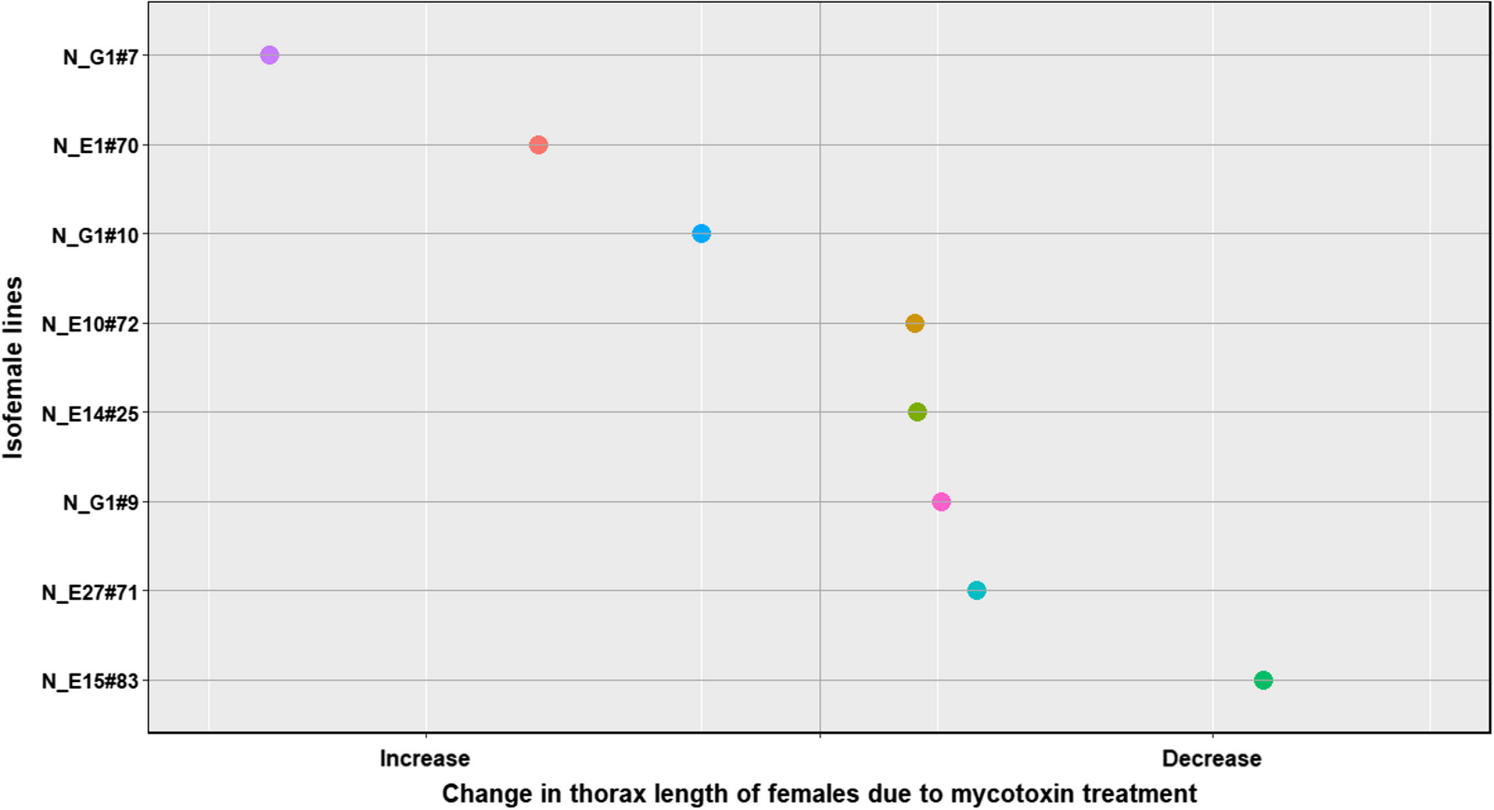
Intraspecific variation in mycotoxin tolerance for thorax length in *D. neotestacea* females

**FIGURE 10:**
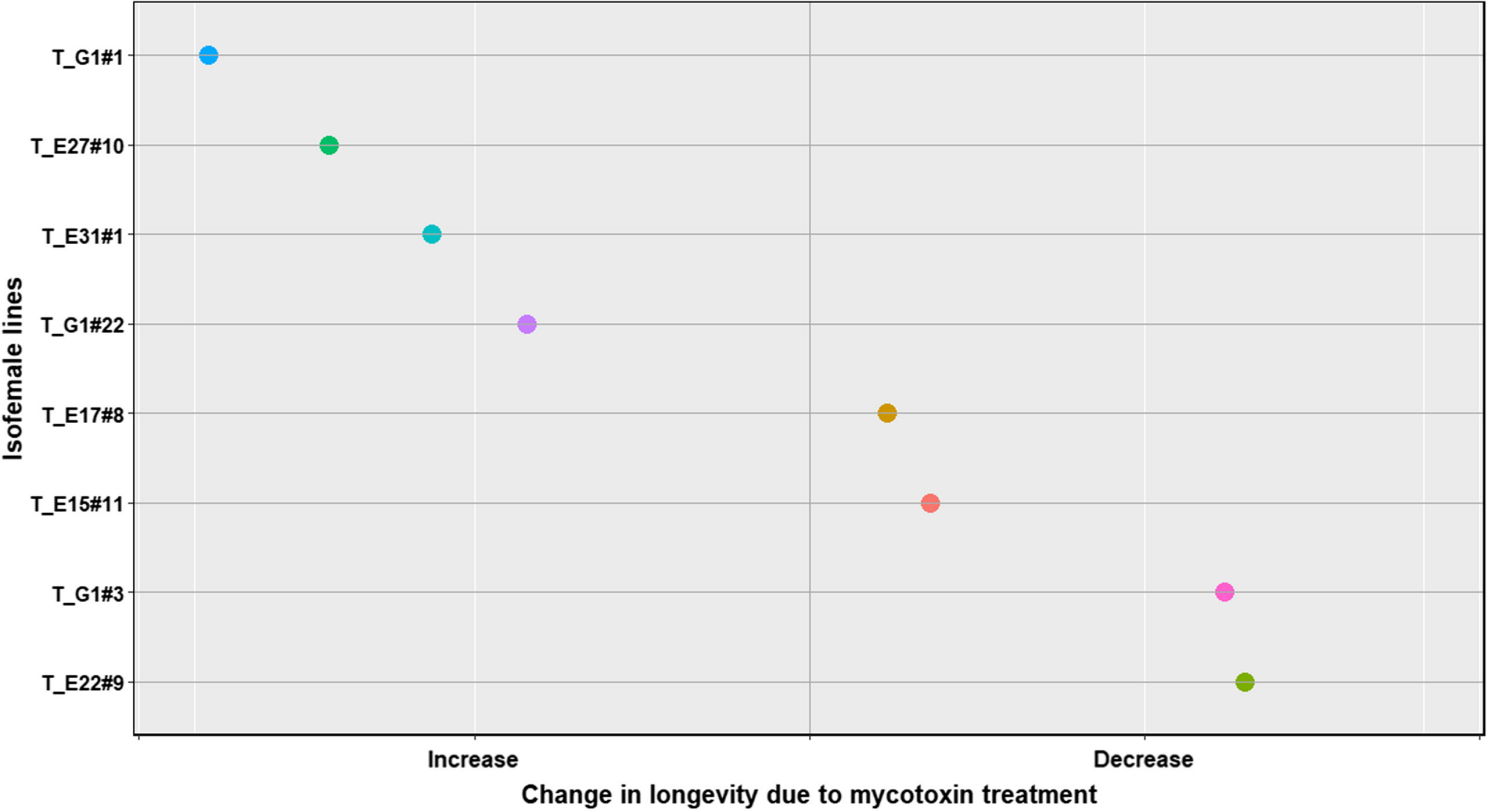
Intraspecific variation in mycotoxin tolerance for longevity in *D. tripunctata*

In all the four species, the traits that were significantly affected by the interaction between treatment and the isofemale line are tabulated in Table 4. All four species (*D. falleni*, *D. recens*, *D. neotestacea*, and *D. tripunctata*) showed intraspecific genetic variation in mycotoxin tolerance for survival to pupation and pupal development time. Additionally, each species showed intraspecific genetic variation in other traits as well (Table 4, Figures 7, 8, 9, 10). Results of the statistical analysis for intraspecific genetic variation in mycotoxin tolerance for all species and traits can be found in Tables S5–S8. The estimates and bounds for each species and trait can be found in Tables S9–S44.

**TABLE 4:** The traits that show the significant effect of interaction between treatment and Isofemale line (false discovery rate < 0.05) and the corresponding chi‐square statistic and its degrees of freedom, the *p*‐value without the adjustment, the *p*‐value after the Bonferroni correction, and the false discovery rate (FDR) calculated based on the Benjamini and Hochberg's method

Species	Traits	Chi‐square statistic	Degrees of freedom	*p*‐value (original)	*p*‐value (Bonferroni)	FDR (BH)
*D. falleni*	Pupal survival	60.34	18	1.80 × 10^−6^	8.10 × 10^−05^	4.05 × 10^−05^
	Pupal development time	111.42	18	1.71 × 10^−15^	7.70 × 10^−14^	7.70 × 10^−14^
	Fly survival	42.97	18	8.08 × 10^−4^	.036	0.009
	Thorax length (males)	22.26	9	.008	.364	0.045
	Longevity	22.44	9	.007	.342	0.045
*D. recens*	Pupal survival	52.76	18	2.86 × 10^−05^	0.00128	6.42 × 10^−04^
	Pupal development time	93.96	18	2.79 × 10^−12^	1.26 × 10–^10^	1.26 × 10^−10^
	Fly survival	43.12	18	7.69 × 10^−04^	.034	0.086
*D. neotestacea*	Pupal survival	64.05	17	2.22 × 10^−07^	8.22 × 10^−06^	4.11 × 10^−06^
	Pupal development time	95.45	16	2.45 × 10^−13^	9.06 × 10^−12^	9.06 × 10^−12^
	Development Time (males)	12.50	1	4.06 × 10^−04^	1.50 × 10^−02^	0.003
	Thorax length (females)	23.18	6	7.39 × 10^−04^	.027	0.004
*D. tripunctata*	Pupal survival	60.27	17	9.49 × 10^−07^	4.27 × 10^−05^	1.07 × 10^−05^
	Pupal development time	124.68	17	1.97 × 10^−18^	<2 × 10^−16^	<2 × 10^−16^
	Fly survival	49.99	16	2.30 × 10^−05^	.001	1.48 × 10^−04^
	Development time (females)	51.49	11	3.37 × 10^−07^	1.52 × 10^−05^	5.06 × 10^−06^
	Thorax length (females)	30.56	11	.001	.058	0.006
	Longevity	20.45	6	.002	.103	0.009

### 3.3 Geographical variation

The location and treatment interaction significantly affected survival to pupation (*p*‐value <.05) and survival to eclosion (*p*‐value <.05) (Tables 1 and 2). Tables S45 and S46 include the estimates, bounds, and effect sizes. Generally, isofemale lines from the GSM location showed a poorer survival due to mycotoxin treatment as compared to the ESC location.

**FIGURE 11:**
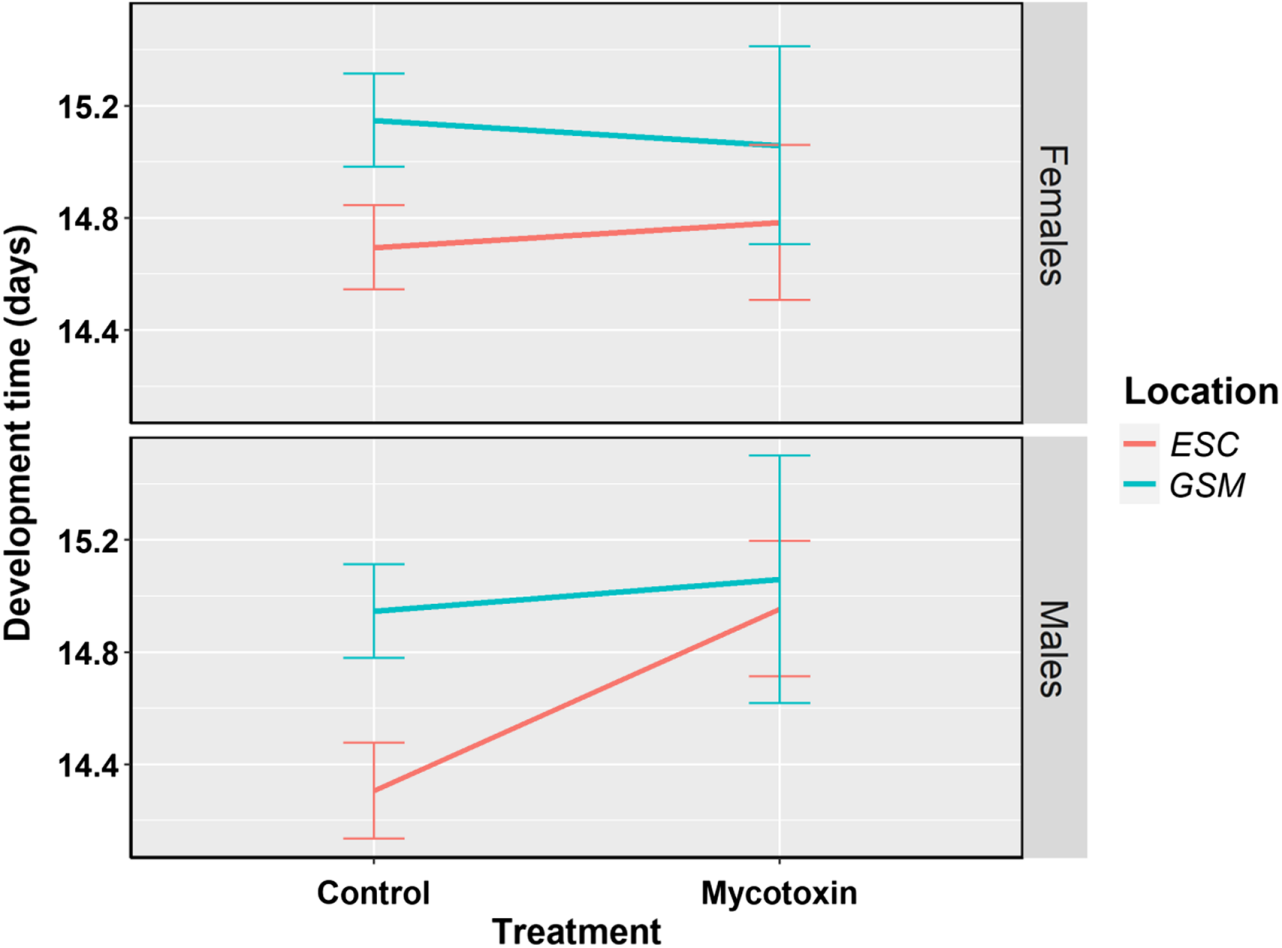
Geographical variation in mycotoxin tolerance for development time in *D. tripunctata*

While performing statistical analyses for intraspecific genetic variation, we also observed that the location and treatment interaction significantly affected the development time in *D. tripunctata*. Males and females of *D. tripunctata* isofemale lines from ESC showed a significant increase in development time as compared to their GSM counterparts (false discovery rate (FDR) < 0.05, each; Figure 11, Table 4, and Table S8).

## 4 DISCUSSION

This study provides three key findings. Firstly, it demonstrates significant interspecific variation in mycotoxin tolerance. Secondly, it shows intraspecific genetic variation for mycotoxin tolerance in each of the four species considered. Thirdly, this study also reveals geographical variation in mycotoxin tolerance between the two locations: Escanaba and the Great Smoky Mountains.

Within the *immigrans*‐*tripunctata* radiation, mycophagy and mycotoxin tolerance have been well established (Bates et al., 2015; Jaenike, 1983; Scott Chialvo & Werner, 2018; Stump et al., 2011; Subramanian & Rup Sarkar, 2015). Therefore, four mycophagous species representing four major clades of the *immigrans*‐*tripunctata* radiation were used in this study to understand the evolution of mycotoxin tolerance in different species. We observed significant interspecific variation in mycotoxin tolerance in four species (*D. falleni*, *D. recens*, *D. tripunctata*, and *D. neotestacea*). *Drosophila falleni* was the most tolerant, followed by *D. recens*, *D. neotestacea*, and *D. tripunctata*, in that order.

Within the *quinaria* species group, *D. falleni* and *D. recens* split ~20 million years ago (Mya) (Izumitani et al., 2016). In our study, *D. falleni* appears to be more mycotoxin‐tolerant than *D. recens*. A comparison between *D. falleni* and *D. recens* has been made previously (Jaenike, 1985), where it was observed that *D. falleni* larvae could not survive to adulthood at α‐amanitin concentrations above 500 μg/ml of food, whereas *D. recens* survived at concentrations up to 1000 μg/ml of food. Furthermore, Stump et al. (2011) observed that *D. falleni* was mycotoxin‐tolerant but showed a statistically significant drop in survival on food containing 50 μg/ml of α‐amanitin. We, on the contrary, have observed *D. falleni* isofemale lines to be highly tolerant. A likely explanation for this discrepancy could be that the previous studies were performed on a single isofemale line. In contrast, we have tested 20 isofemale lines per species, which provided a better dataset to estimate interspecific variation.

The majority of isofemale lines of *D. neotestacea* (12/20) and *D. tripunctata* (16/20) (representative species of the *tripunctata* and *testacea* species groups, respectively) showed low mycotoxin tolerance. Furthermore, these species showed reduced overall survival in the presence of the natural‐toxin mix. Our observations suggest that species from the *quinaria* species group (*D. falleni* and *D. recens*) have retained the mycotoxin tolerance trait better than species from the *testacea* and *tripunctata* species groups. It is worth mentioning that the *tripunctata* and the *testacea* species groups have diverged from the *quinaria* species group ~27 Mya (Izumitani et al., 2016). We suggest that different selective pressures that have brought about the divergent evolution and speciation among these four species have also affected mycotoxin tolerance. It would be interesting to identify the genetic and genomic changes that may have altered the extent of mycotoxin tolerance among these species.

We used the isofemale line technique to investigate the intraspecific genetic variation of mushroom toxin tolerance (Hoffmann & Parsons, 1988). This technique is based on a simple concept that when isofemale lines from wild‐collected females are established, and their progeny is maintained under similar laboratory conditions, the variation observed among the isofemale lines is primarily genetic. Phenotypic variation for a trait among these genetically distinct isofemale lines would indicate intraspecific genetic variation attributed mainly to segregating alleles at multiple loci (Mackay, 2010). Our study found phenotypic variation in mycotoxin tolerance among isofemale lines in all four species, providing evidence for intraspecific genetic variation for mycotoxin tolerance. Intraspecific genetic variation in mycotoxin tolerance has been reported previously in *D*. *tripunctata* (Jaenike, 1989). Our results confirm their findings and expand the dataset to include three additional species (*D. falleni*, *D. recens*, and *D. neotestacea*). This study provides the groundwork for further studies to calculate the heritability and identify the genetic architecture of the mycotoxin tolerance trait.

Geographical variation and the evolutionary forces are of particular interest to evolutionary biologists. Geographical variation has been reported in a wide range of phenotypes, from acoustic signals in animals (Zhang et al., 2018) to the chemical composition of phenolics in plants (Liu et al., 2018). In essence, if a trait shows differences between populations from different geographical locations, the trait is considered to demonstrate geographical variation. In our study, we found geographical variation in mycotoxin tolerance on two accounts. First, the isofemale lines from GSM appeared to be more vulnerable to mycotoxin treatment compared to isofemale lines from ESC. Second, the *D. tripunctata* isofemale lines from ESC showed a significant increase in the development time of males and females compared to their GSM counterparts.

The two locations (ESC and GSM) used in our study have distinct abiotic factors (Table 5), potentially affecting many biotic factors. Although we cannot pinpoint what factor(s) may be influencing the mycotoxin tolerance trait, we can safely state that certain factors at each location act as strong selective forces and that adaptation to the local abiotic and biotic conditions shape the genome of a species and in the process, affect the mycotoxin tolerance trait. Further studies are required to identify the specific environmental factors that play a critical role in the evolution of the mycotoxin tolerance trait.

**TABLE 5:** Table depicting the environmental conditions at the two locations: GSM and ESC

	GSM	ESC
Coordinates	35.6532°N, 83.5070°W	45.7452°N, 87.0646°W
Average Rainfall (inches)	55 (Valleys); 85 (Peaks)	29.4
Precipitation (days)	123	121.4
Daylight during the coldest month (January)	10 ½ h	9 h
Daylight during the coldest month (January)	14 ½ h	15 ½ h
Temperature during the coldest month (January)°C	−7 (low)/3 (high)	−12 (low)/−3 (high)
Temperature during the warmest month (July)°C	9 (low)/30 (high)	13 (low)/24 (high)

All aforementioned conclusions are based on the results from the linear mixed model or the binomial linear mixed effects model with the logistic link function implemented in R package ‘lme4’ (Bates et al., 2015). In all models, the replicate vials and/or the isofemale lines were included as the random effects. It is well known that the binomial linear mixed model may not be sufficient due to overdispersion. Among 19 estimated overdispersion parameters, 16 of them were between 0.93 and 1.52, and only three of them were greater than 1.60 (1.61, 1.86, 2.02, see Figure S34), indicating that the overdispersion was not a serious problem. The scatter plots of the deviance residuals and the predicted values (see Figures S1–S33) show that most of the model fittings were adequate.

In conclusion, our study identifies interspecific and intraspecific variation in mycotoxin tolerance and demonstrates geographical variation in mycotoxin tolerance.

## AUTHOR CONTRIBUTIONS

**Prajakta P. Kokate:** Conceptualization (equal); data curation (lead); formal analysis (lead); investigation (lead); methodology (lead); validation (lead); visualization (lead); writing – original draft (lead); writing – review and editing (supporting). **Morgan Smith:** Investigation (supporting); methodology (supporting); writing – review and editing (supporting). **Lucinda Hall:** Investigation (supporting); methodology (supporting); writing – review and editing (supporting). **Kui Zhang:** Formal analysis (supporting); software (equal); validation (supporting); visualization (supporting); writing – review and editing (supporting). **Thomas Werner:** Conceptualization (equal); data curation (supporting); formal analysis (supporting); funding acquisition (lead); investigation (supporting); methodology (equal); project administration (lead); resources (equal); supervision (lead); validation (supporting); visualization (supporting); writing – original draft (supporting); writing – review and editing (lead).

## CONFLICT OF INTEREST

There are no competing interests.

## Supporting information


Appendix S1

## Data Availability

Phenotype data and the code for running analyses are available in Dryad, https://doi.org/10.5061/dryad.2ngf1vhr7.
