# Supplementary material for: Inter‐ and intraspecific variation in mycotoxin tolerance: A study of four Drosophila species
Source: Ecol Evol. 2022 Jul 24;12(7):e9126. doi: 10.1002/ece3.9126 (PMC9309036; doi:10.1002/ece3.9126)
Supplement: Supplementary file 1 — Appendix S1 [file ECE3-12-e9126-s001.zip › ECE3_9126_Supplement.pdf]

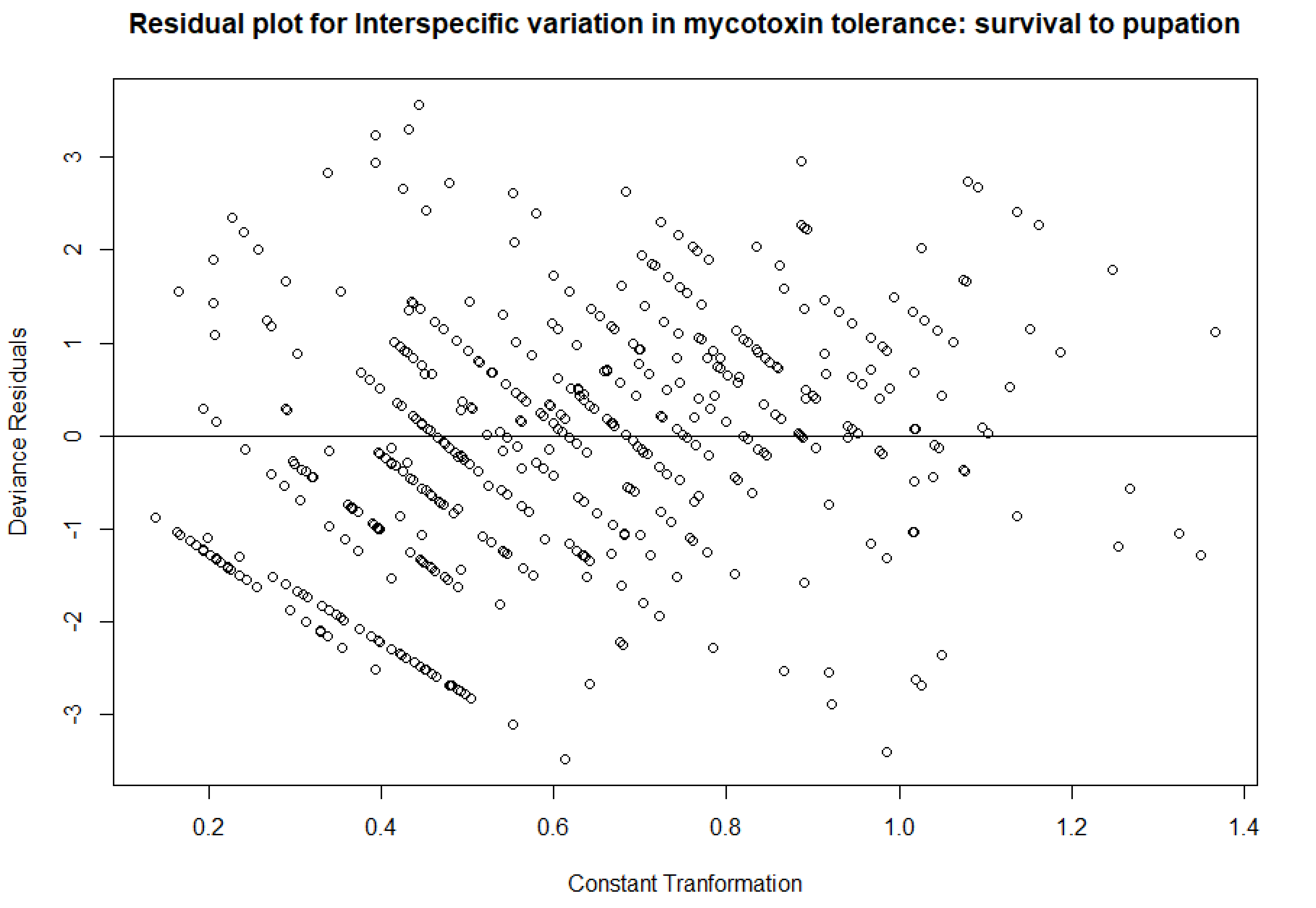
Residual plot for Interspecific variation in mycotoxin tolerance: survival to pupation
Deviance Residuals
3
2
1
0
-1
-2
-3
0.2
0.4
0.6
0.8
1.0
1.2
1.4
Constant Tranformation

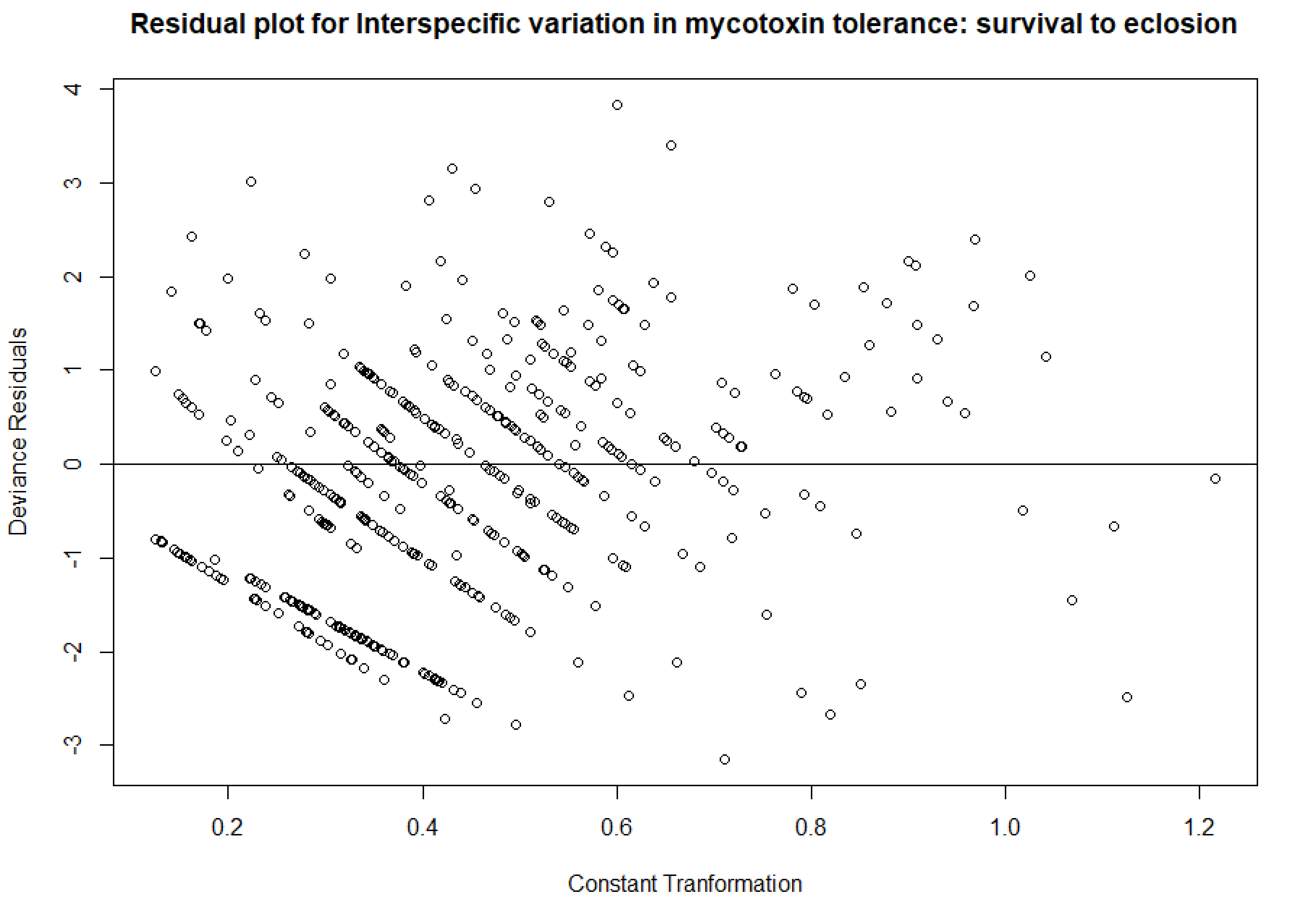
Residual plot for Interspecific variation in mycotoxin tolerance: survival to eclosion
Deviance Residuals
-3
-2
-1
0
1
2
3
4
0.2
0.4
0.6
0.8
1.0
1.2
Constant Tranformation

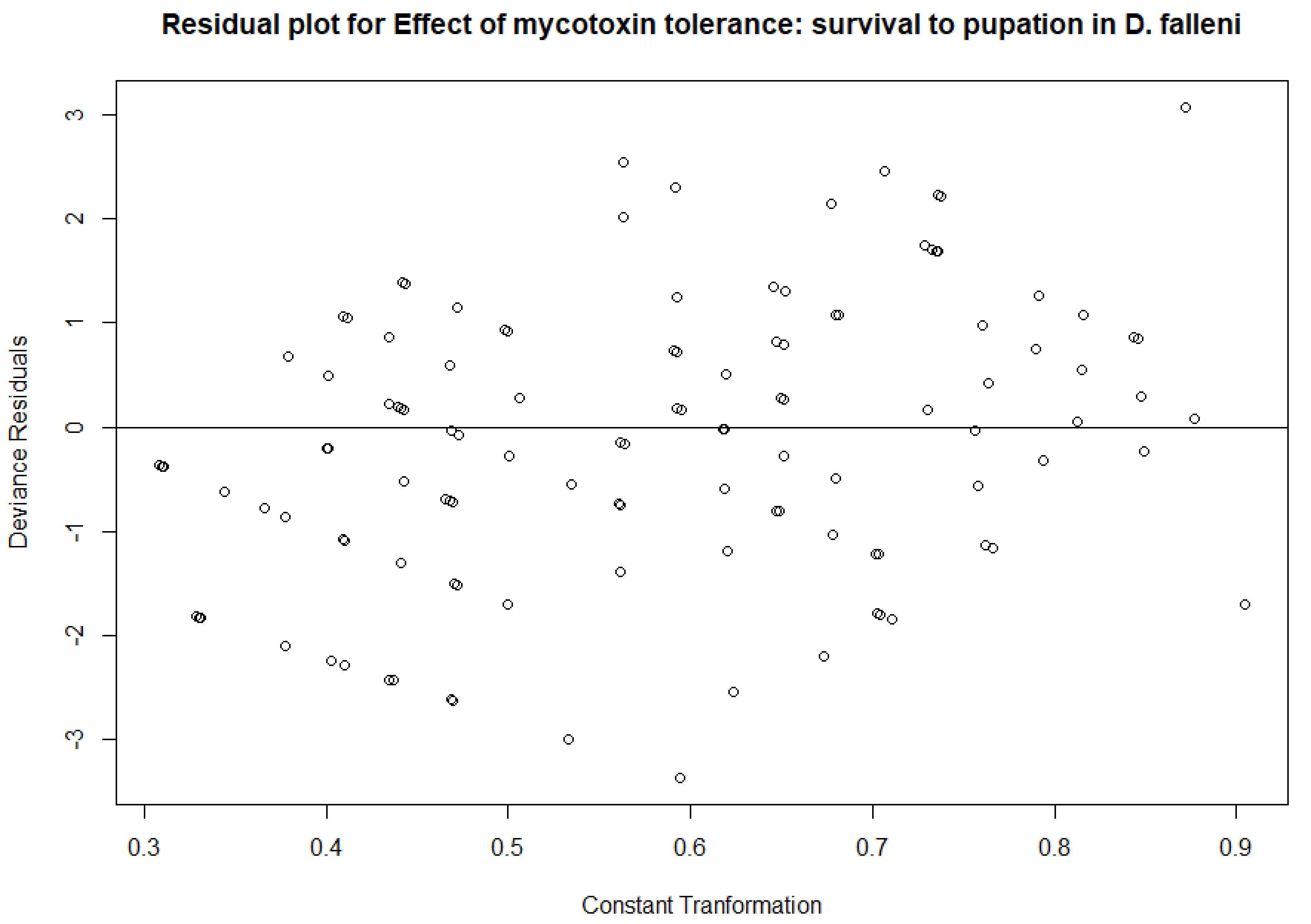
Residual plot for Effect of mycotoxin tolerance: survival to pupation in D. falleni
Deviance Residuals
Constant Tranformation
3
2
1
0
-1
-2
-3
0.3
0.4
0.5
0.6
0.7
0.8
0.9

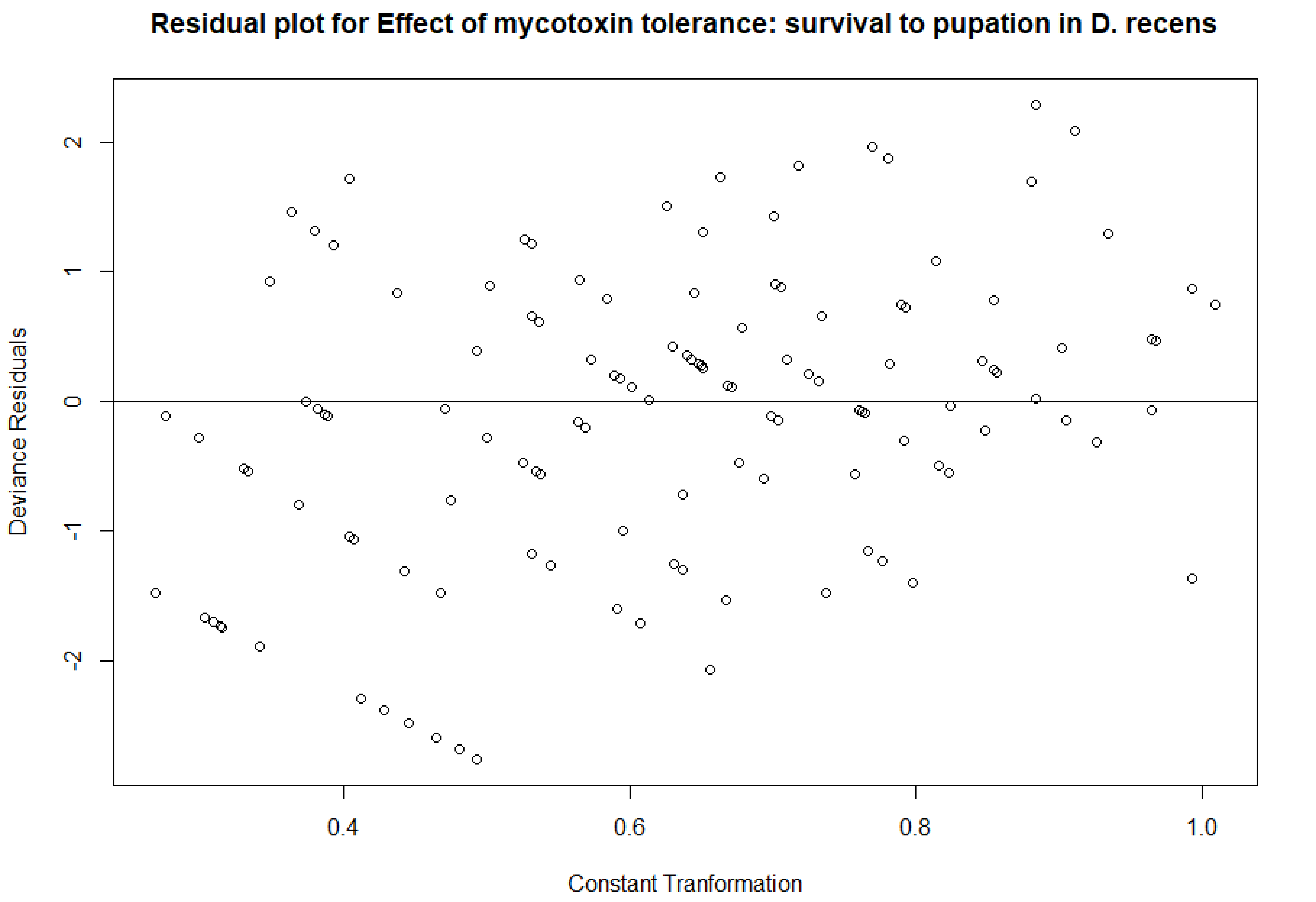

Residual plot for Effect of mycotoxin tolerance: survival to pupation in D. recens
Deviance Residuals
2
1
0
-1
-2
0.4
0.6
0.8
1.0
Constant Tranformation

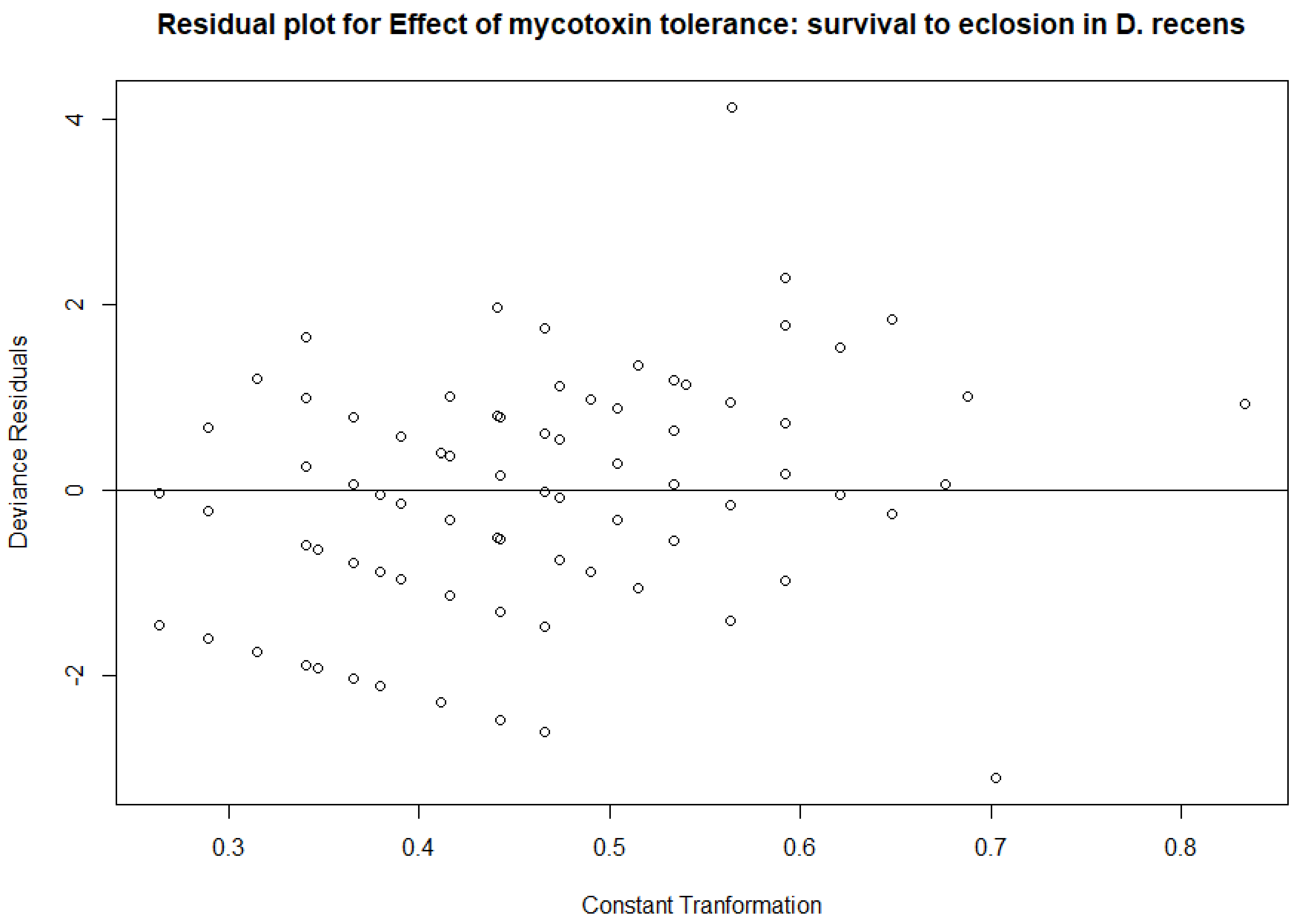

Residual plot for Effect of mycotoxin tolerance: survival to eclosion in D. recens
Deviance Residuals
-2
0
2
4
0.3
0.4
0.5
0.6
0.7
0.8
Constant Tranformation

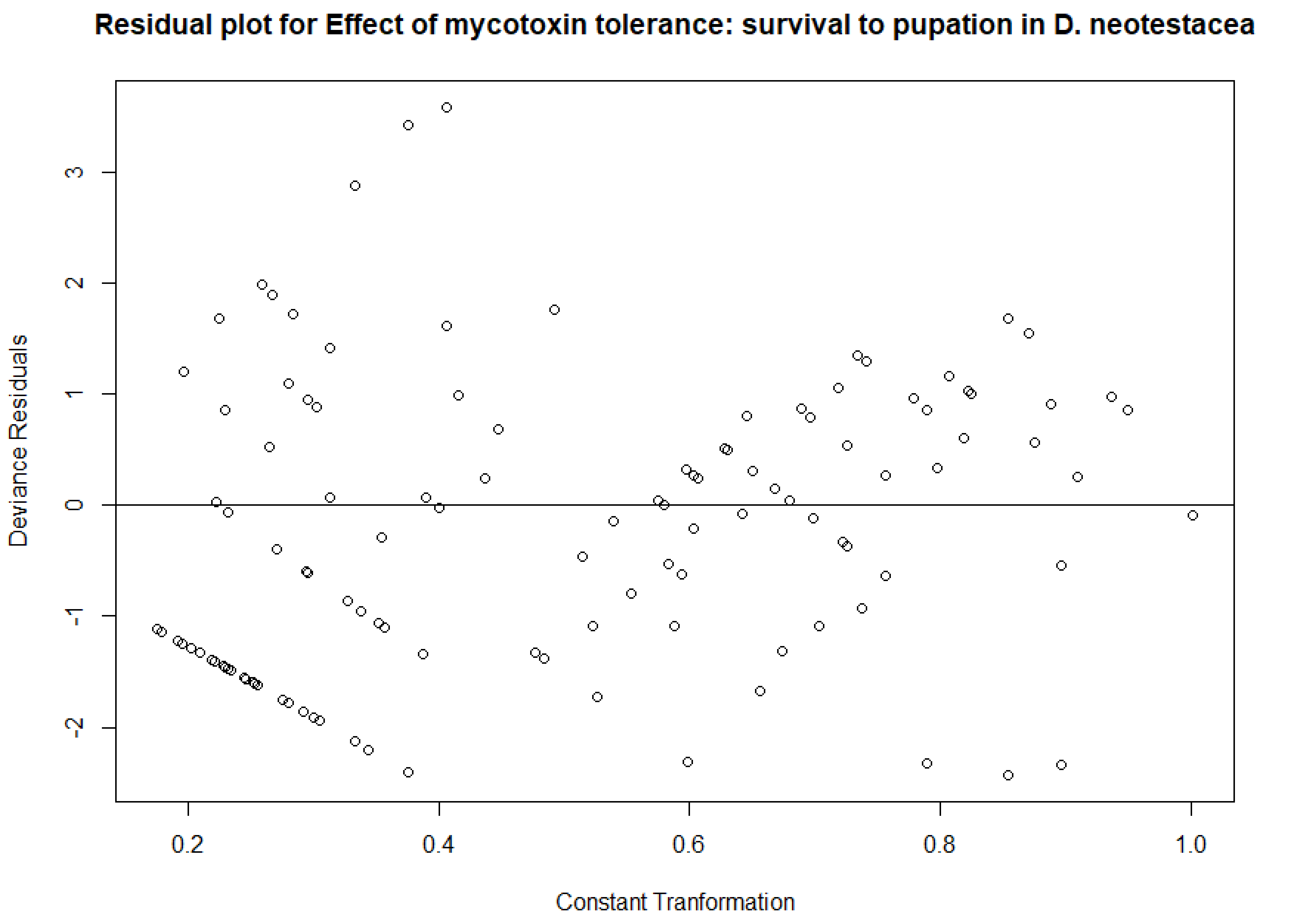
Residual plot for Effect of mycotoxin tolerance: survival to pupation in D. neotestacea
Deviance Residuals
Constant Tranformation
3
2
1
0
-1
-2
0.2
0.4
0.6
0.8
1.0

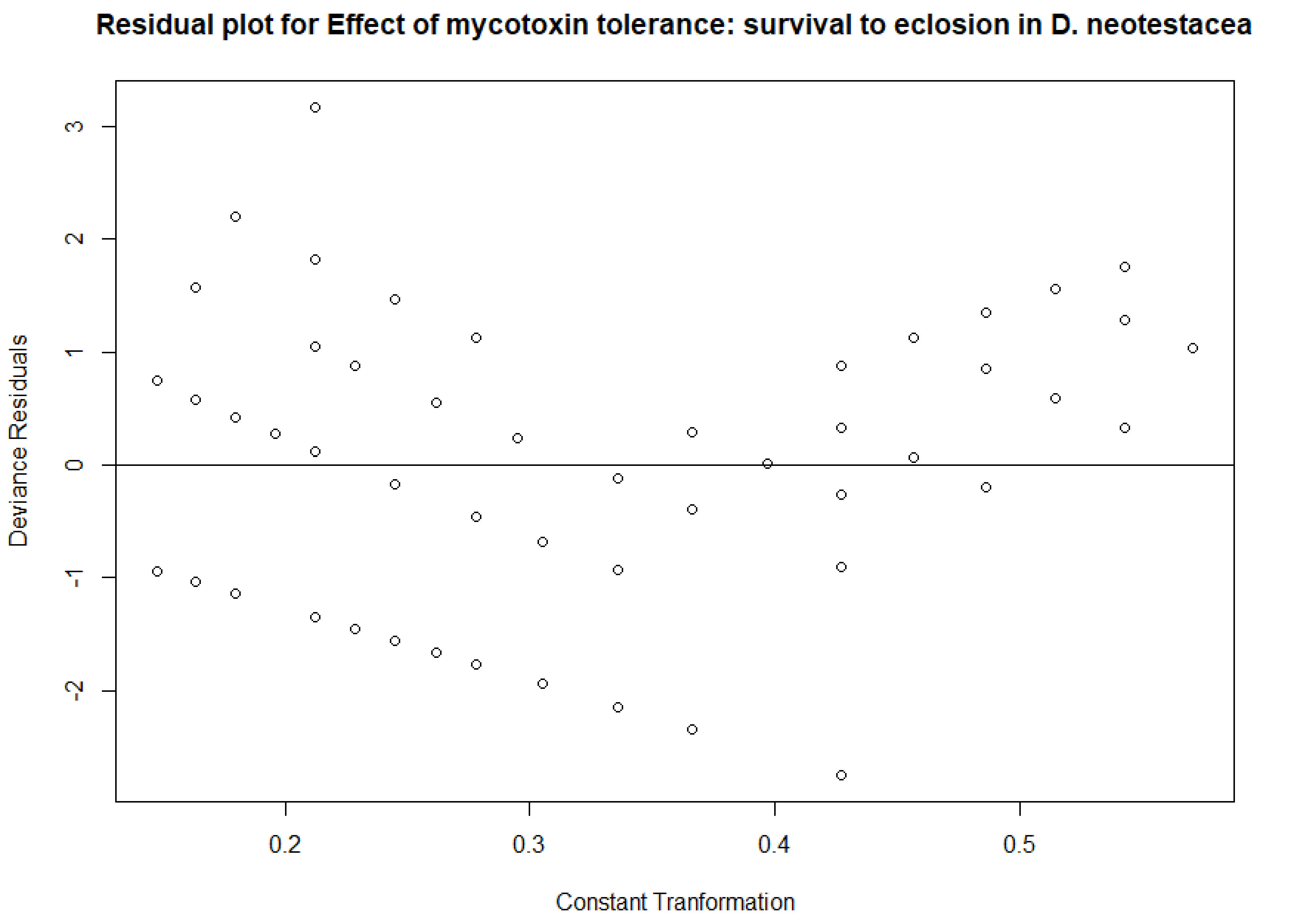
Residual plot for Effect of mycotoxin tolerance: survival to eclosion in D. neotestacea
Deviance Residuals
-2
-1
0
1
2
3
0.2
0.3
0.4
0.5
Constant Tranformation

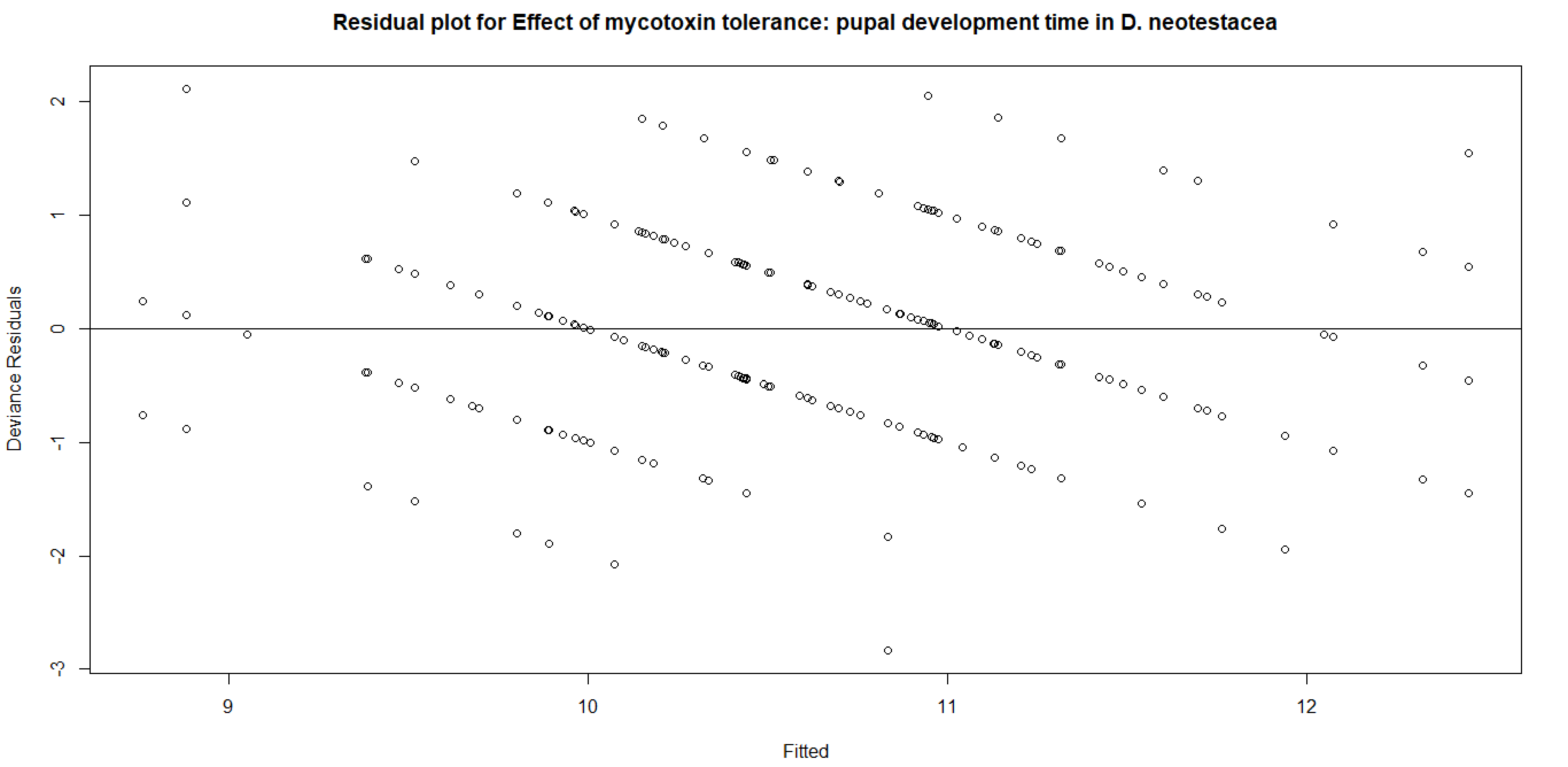
Residual plot for Effect of mycotoxin tolerance: pupal development time in D. neotestacea
Deviance Residuals
Fitted
2
1
0
-1
-2
-3
9
10
11
12

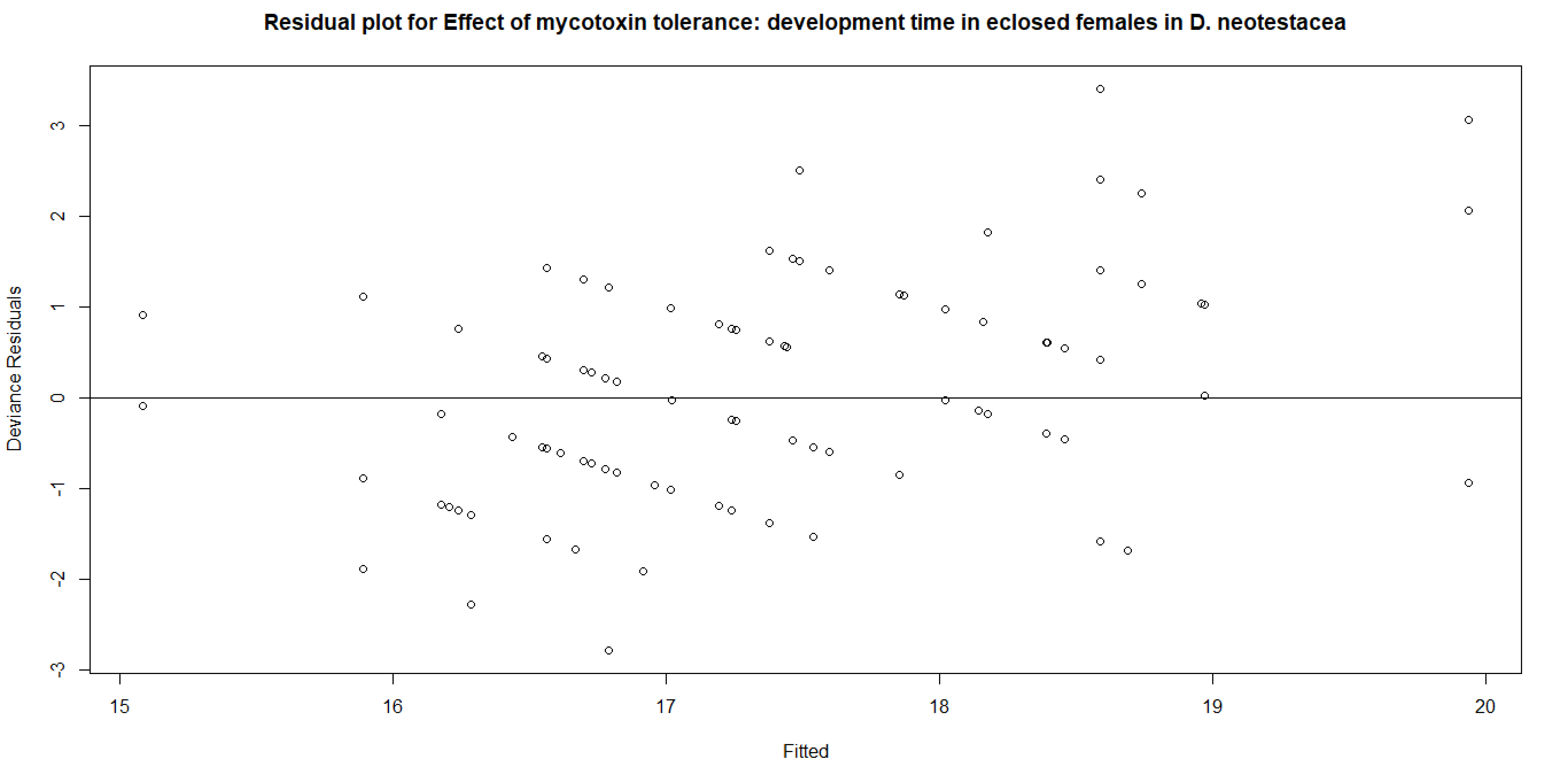
Residual plot for Effect of mycotoxin tolerance: development time in eclosed females in D. neotestacea
Deviance Residuals
Fitted
-3
-2
-1
0
1
2
3
15
16
17
18
19
20

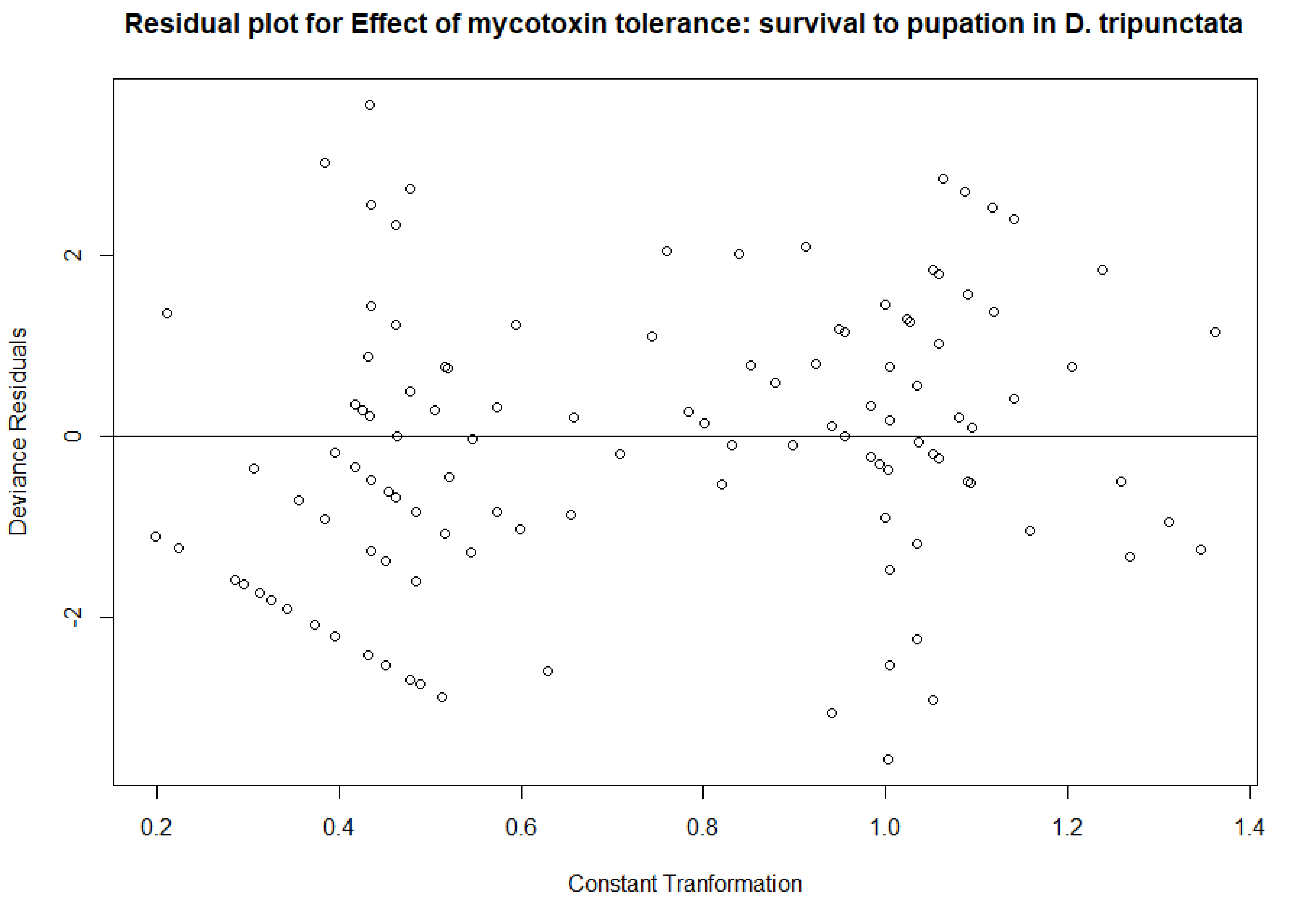

Residual plot for Effect of mycotoxin tolerance: survival to pupation in D. tripunctata
Deviance Residuals
Constant Tranformation
2
0
-2
0.2
0.4
0.6
0.8
1.0
1.2
1.4

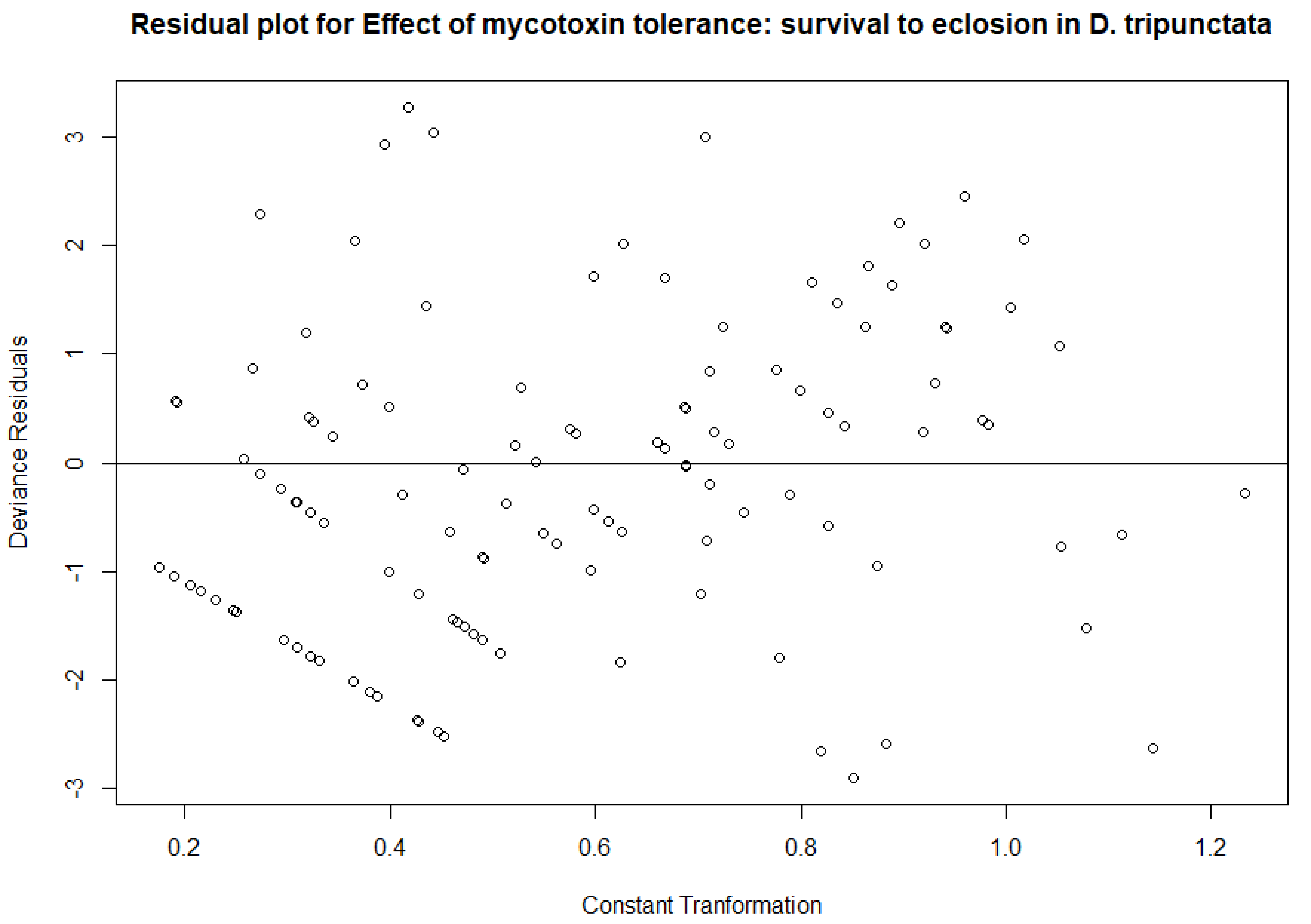

Residual plot for Effect of mycotoxin tolerance: survival to eclosion in D. tripunctata
Deviance Residuals
Constant Tranformation
3
2
1
0
-1
-2
-3
0.2
0.4
0.6
0.8
1.0
1.2

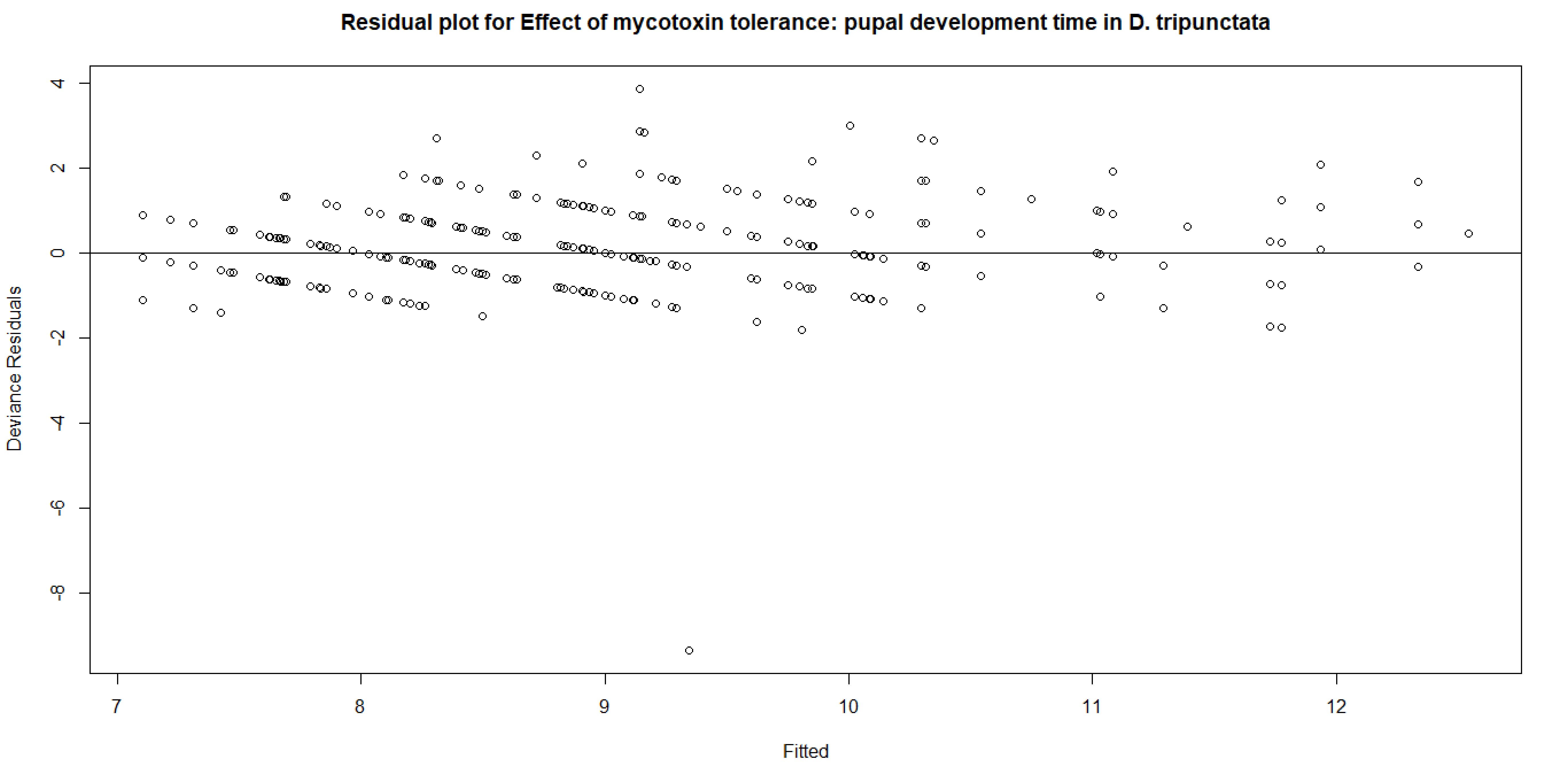
Residual plot for Effect of mycotoxin tolerance: pupal development time in D. tripunctata
Deviance Residuals
4
2
0
-2
-4
-6
-8
7
8
9
10
11
12
Fitted

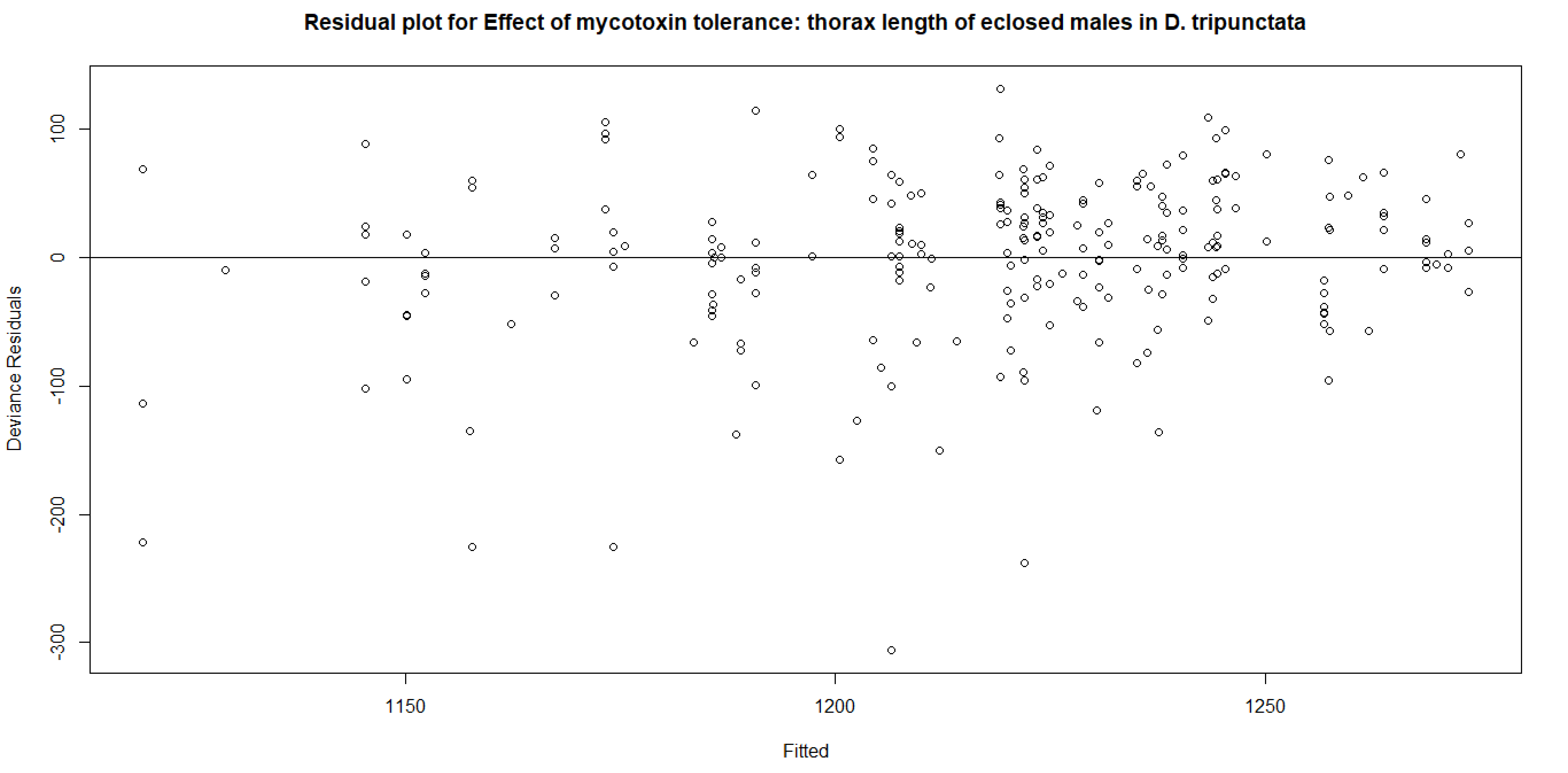
Residual plot for Effect of mycotoxin tolerance: thorax length of eclosed males in D. tripunctata
Deviance Residuals
100
0
-100
-200
-300
1150
1200
1250
Fitted

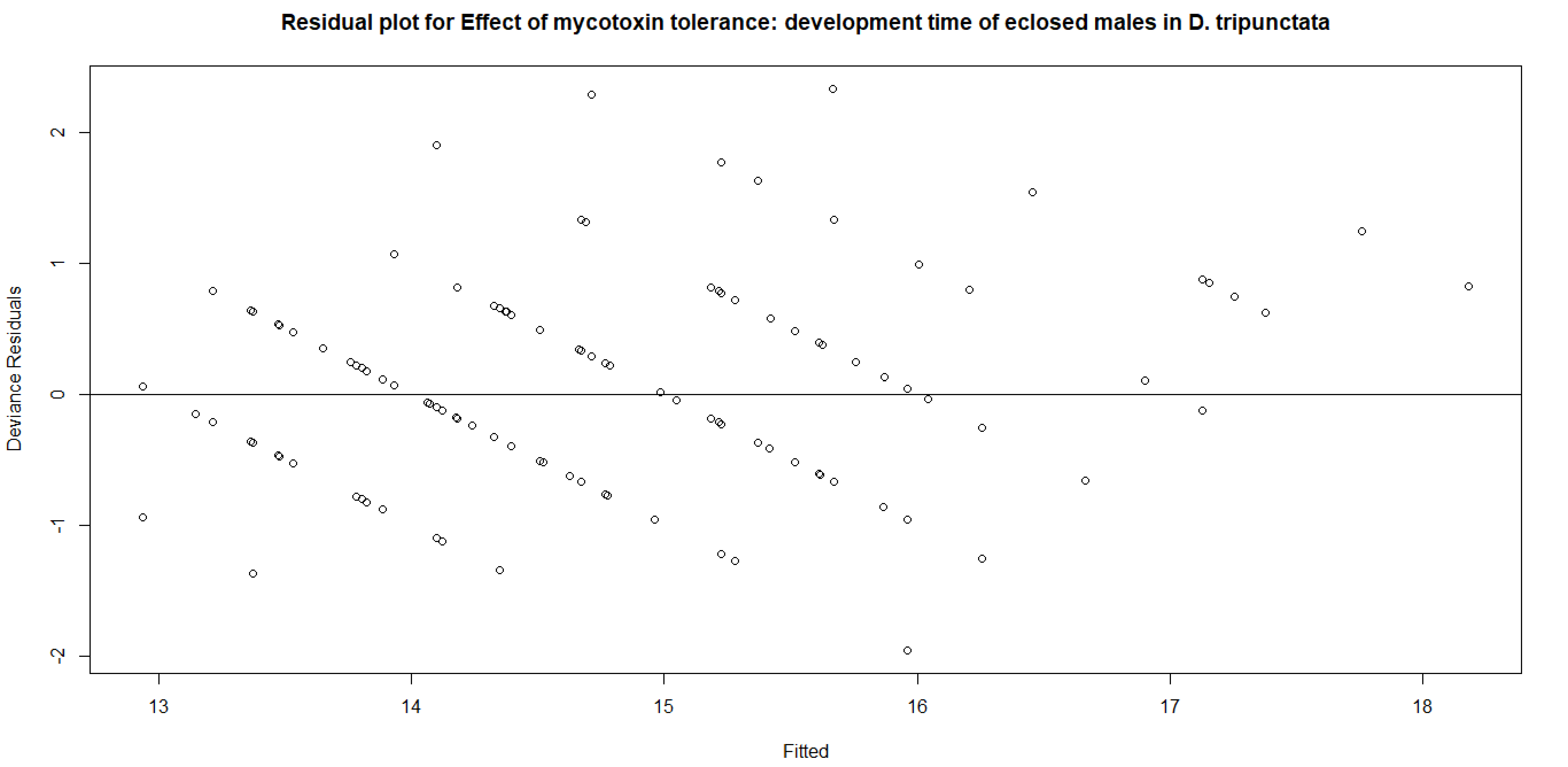

Residual plot for Effect of mycotoxin tolerance: development time of eclosed males in D. tripunctata
Deviance Residuals
2
1
0
-1
-2
13
14
15
16
17
18
Fitted

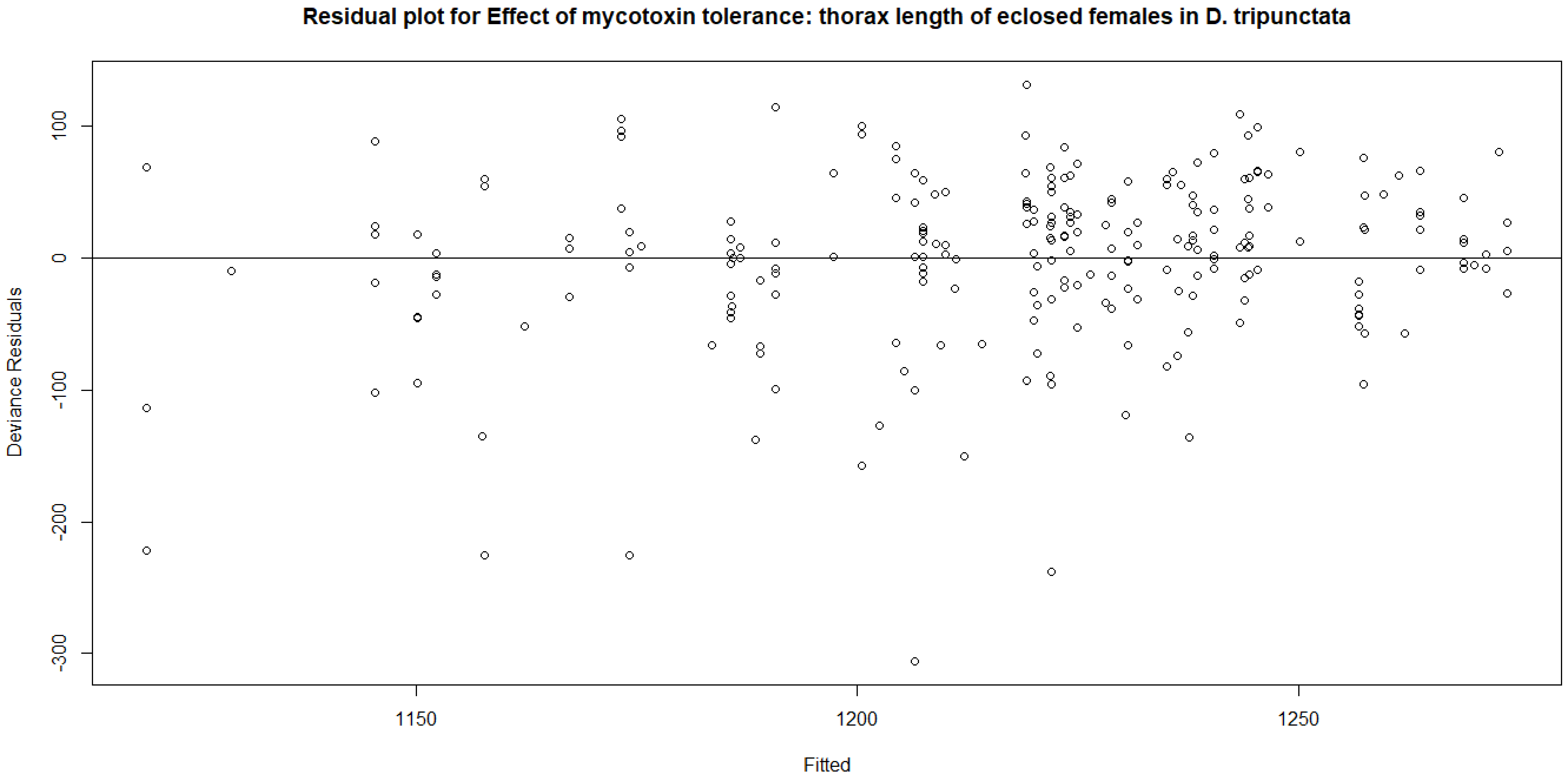
Residual plot for Effect of mycotoxin tolerance: thorax length of eclosed females in D. tripunctata
Deviance Residuals
100
0
-100
-200
-300
1150
1200
1250
Fitted

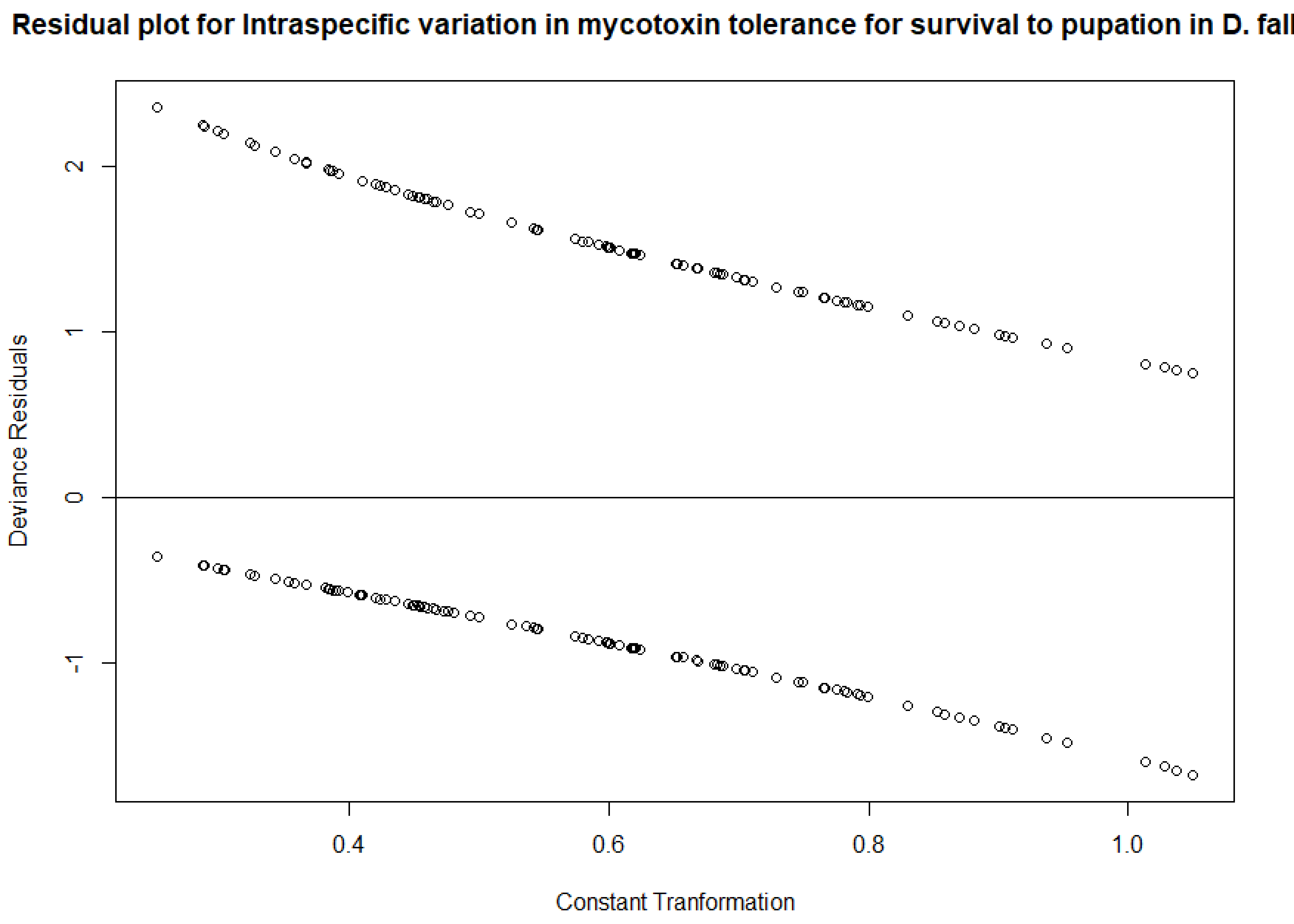

Residual plot for Intraspecific variation in mycotoxin tolerance for survival to pupation in D. fall
Deviance Residuals
2
1
0
-1
0.4
0.6
0.8
1.0
Constant Tranformation

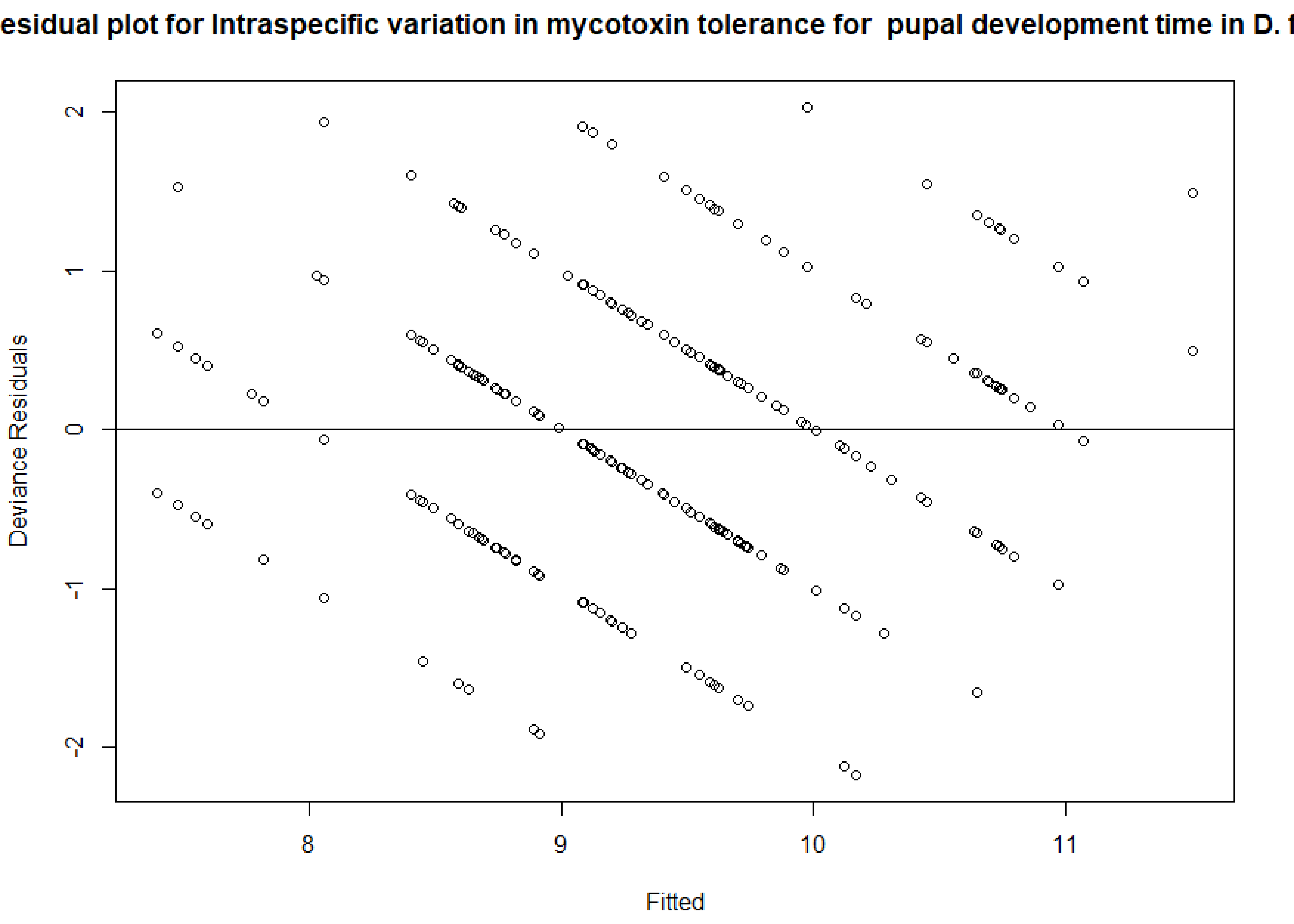
esidual plot for Intraspecific variation in mycotoxin tolerance for pupal development time in D. f
Deviance Residuals
-2
-1
0
1
2
Fitted
8
9
10
11

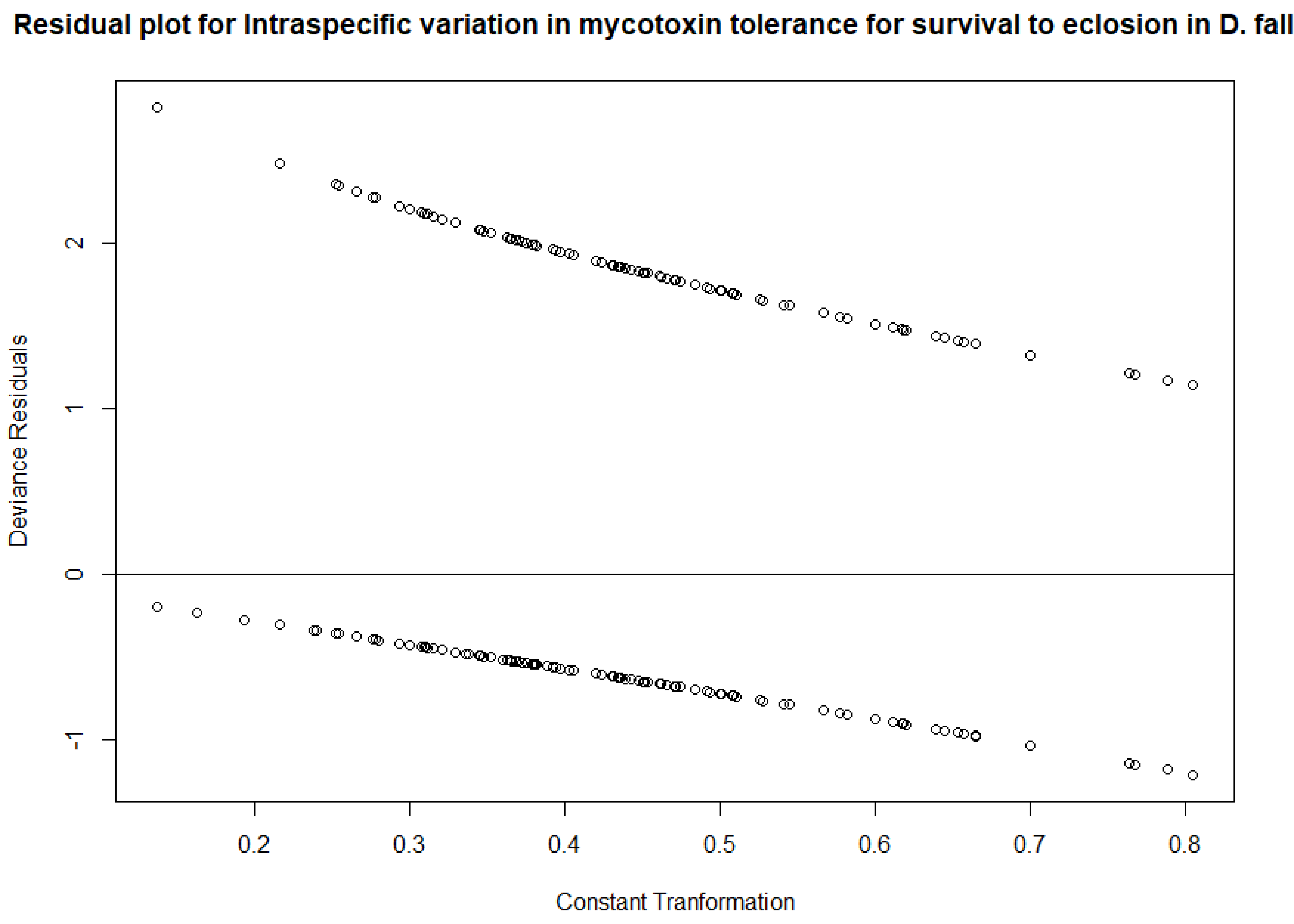
Residual plot for Intraspecific variation in mycotoxin tolerance for survival to eclosion in D. fall
Deviance Residuals
2
1
0
-1
0.2
0.3
0.4
0.5
0.6
0.7
0.8
Constant Tranformation

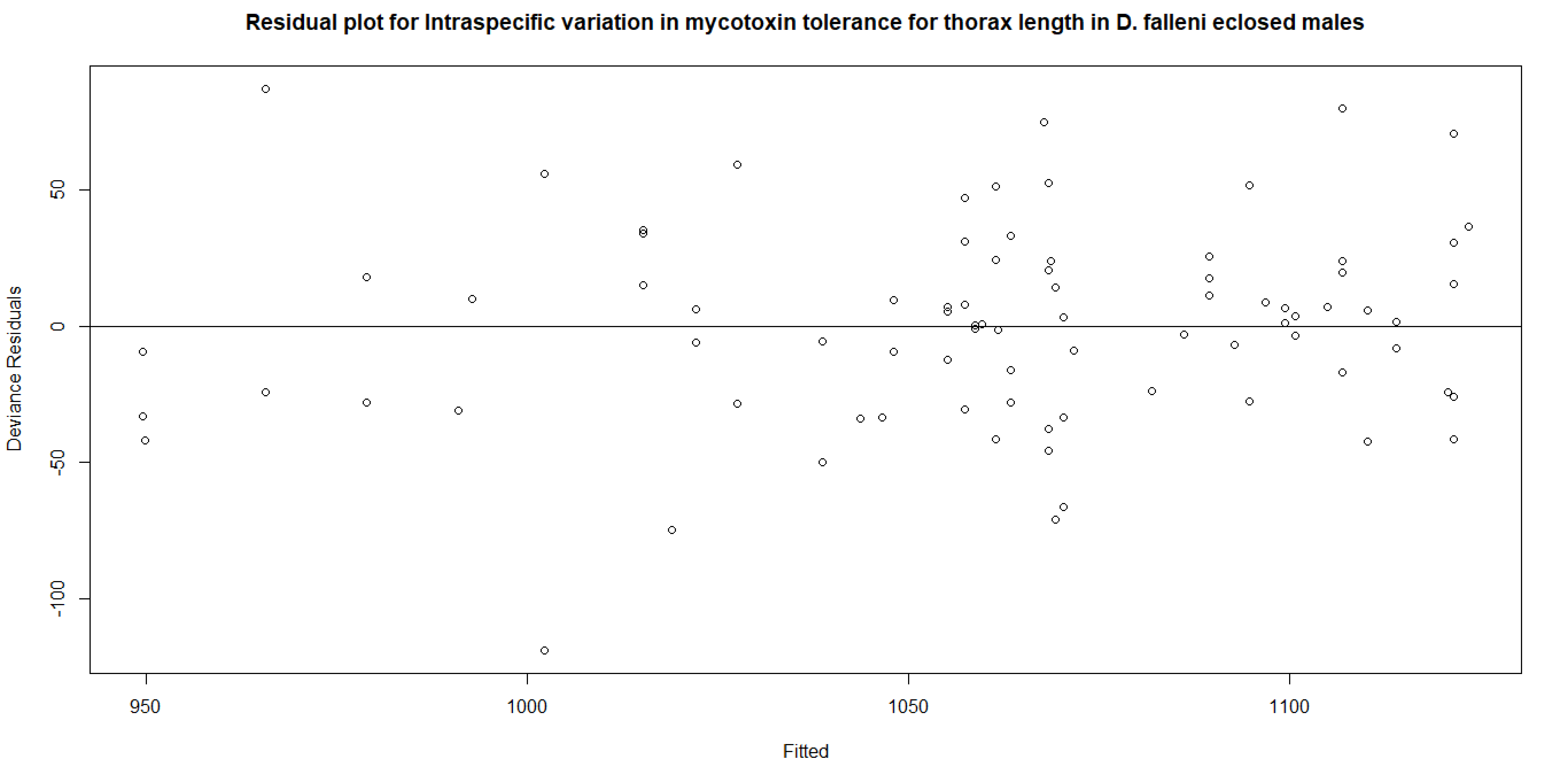

Residual plot for Intraspecific variation in mycotoxin tolerance for thorax length in D. falleni eclosed males
Deviance Residuals
50
0
-50
-100
950
1000
1050
1100
Fitted

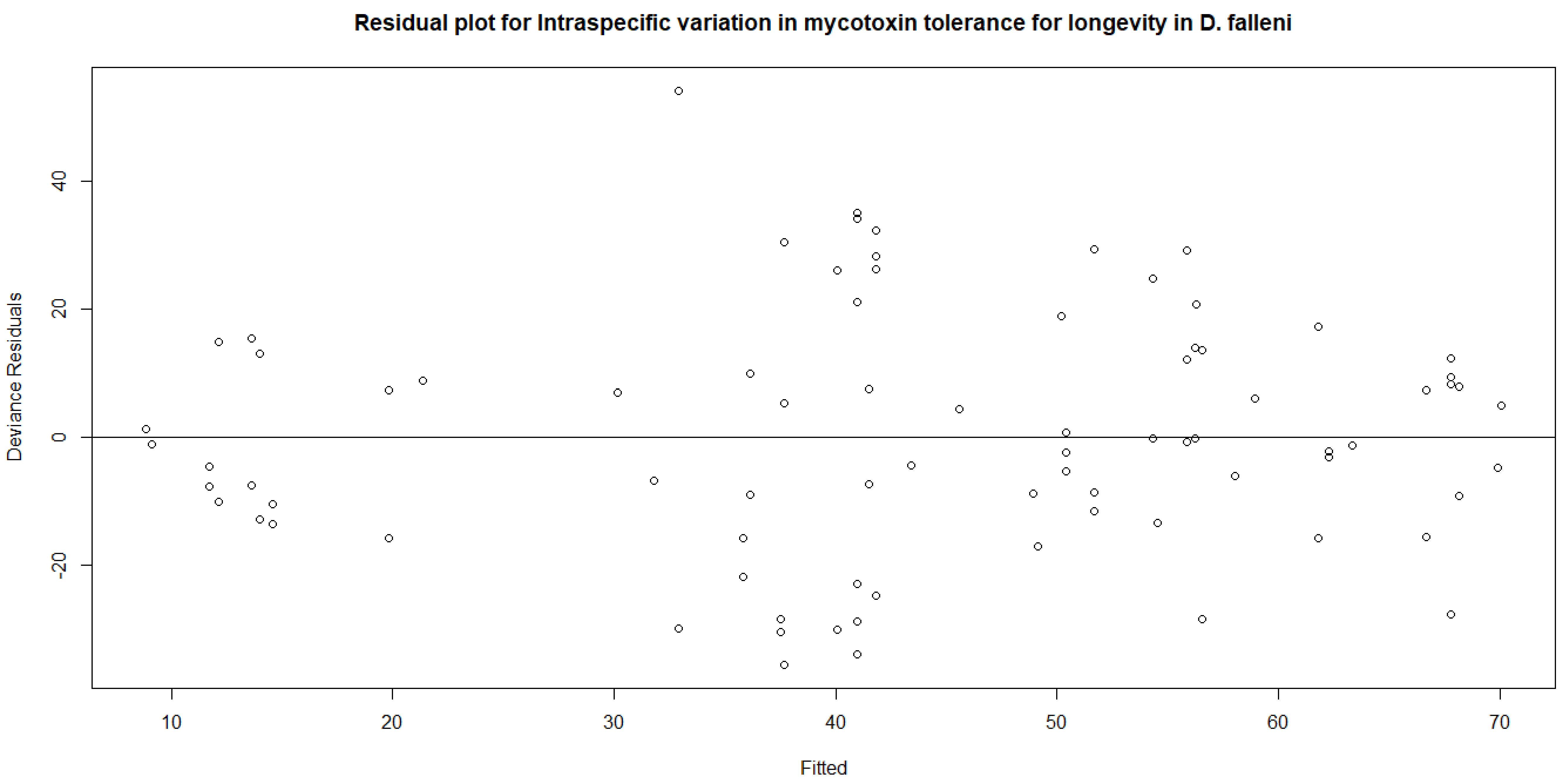
Residual plot for Intraspecific variation in mycotoxin tolerance for longevity in D. falleni
Deviance Residuals
Fitted
-20
0
20
40
10
20
30
40
50
60
70

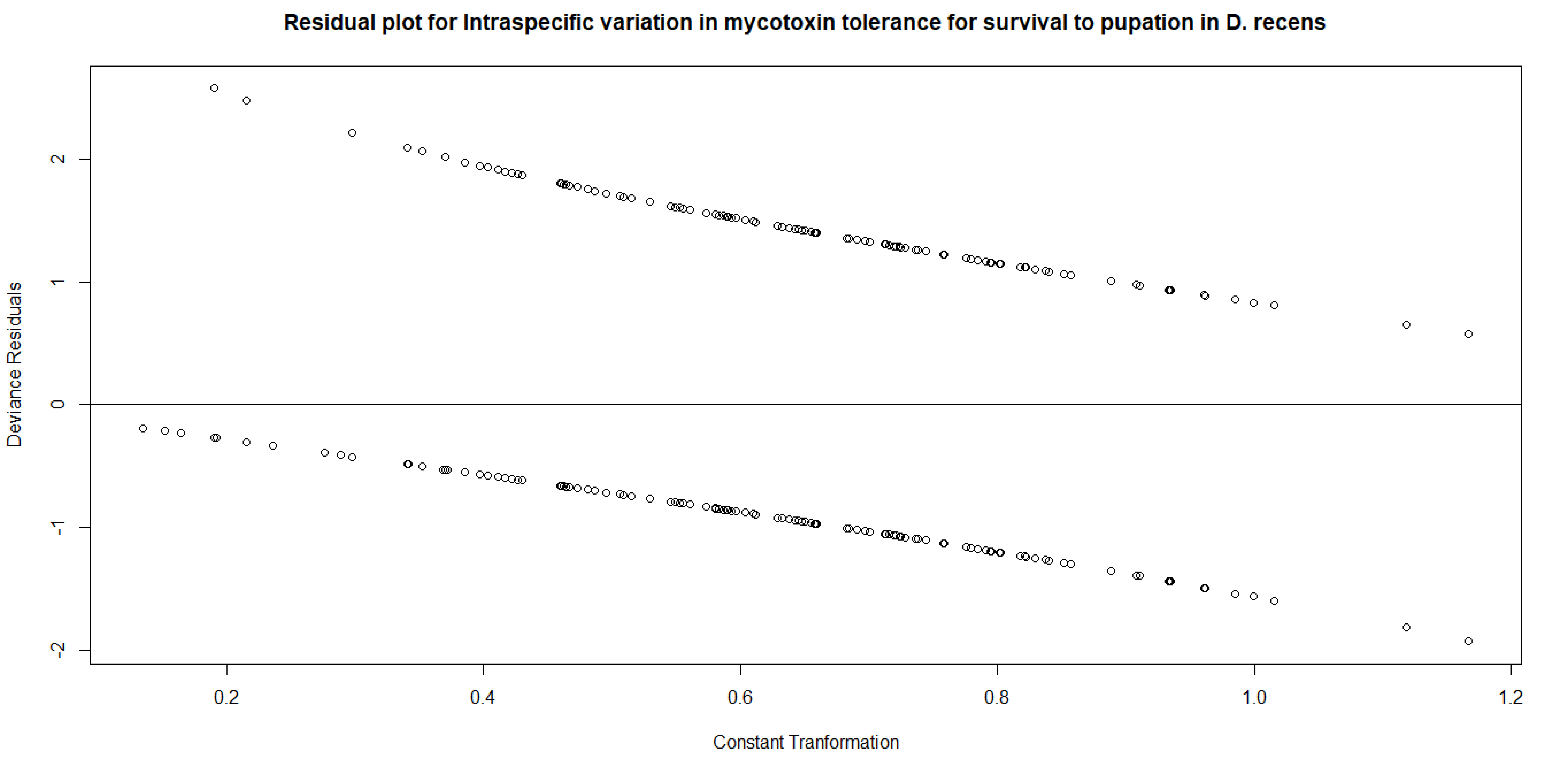

Residual plot for Intraspecific variation in mycotoxin tolerance for survival to pupation in D. recens
Deviance Residuals
Constant Tranformation
2
1
0
-1
-2
0.2
0.4
0.6
0.8
1.0
1.2

Residual plot for Intraspecific variation in mycotoxin tolerance for pupal development time in D. recens

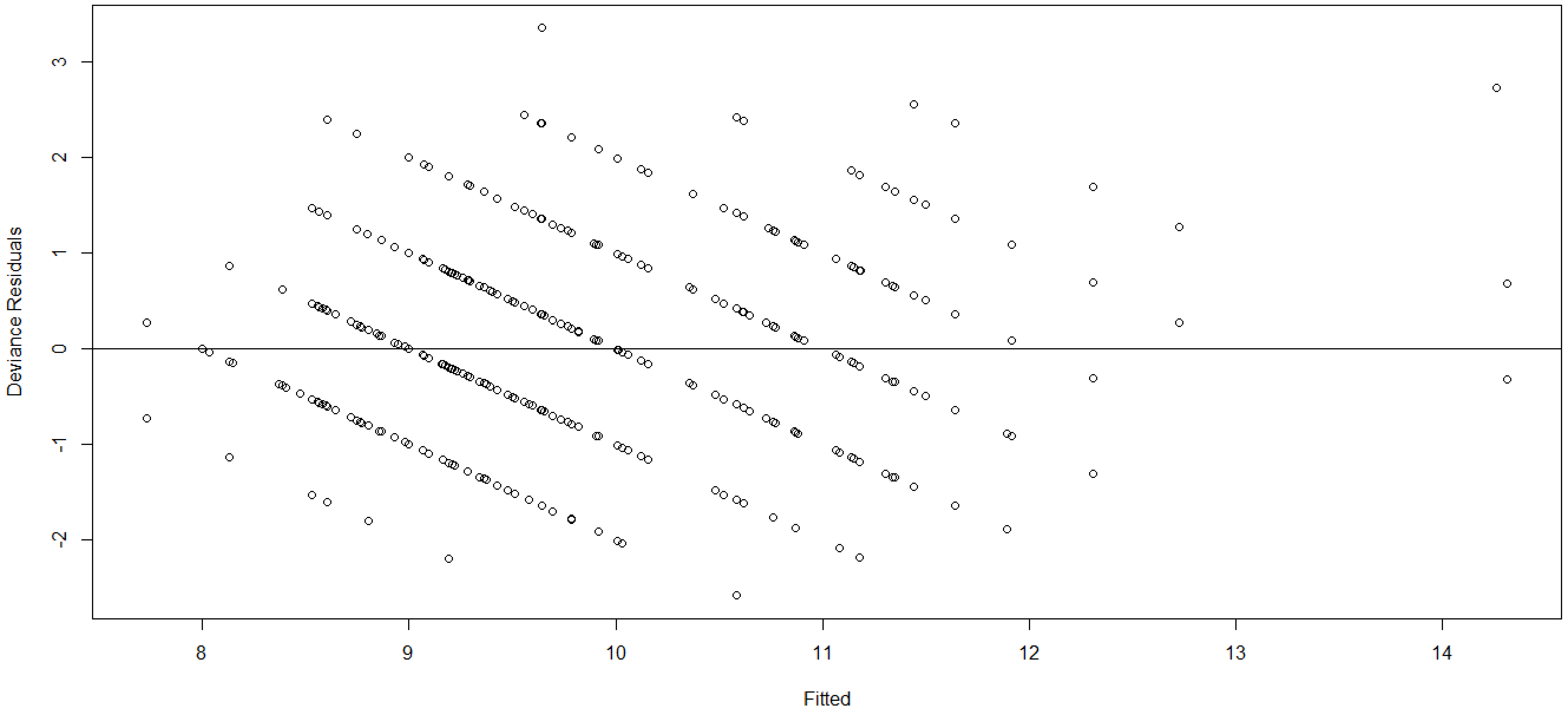

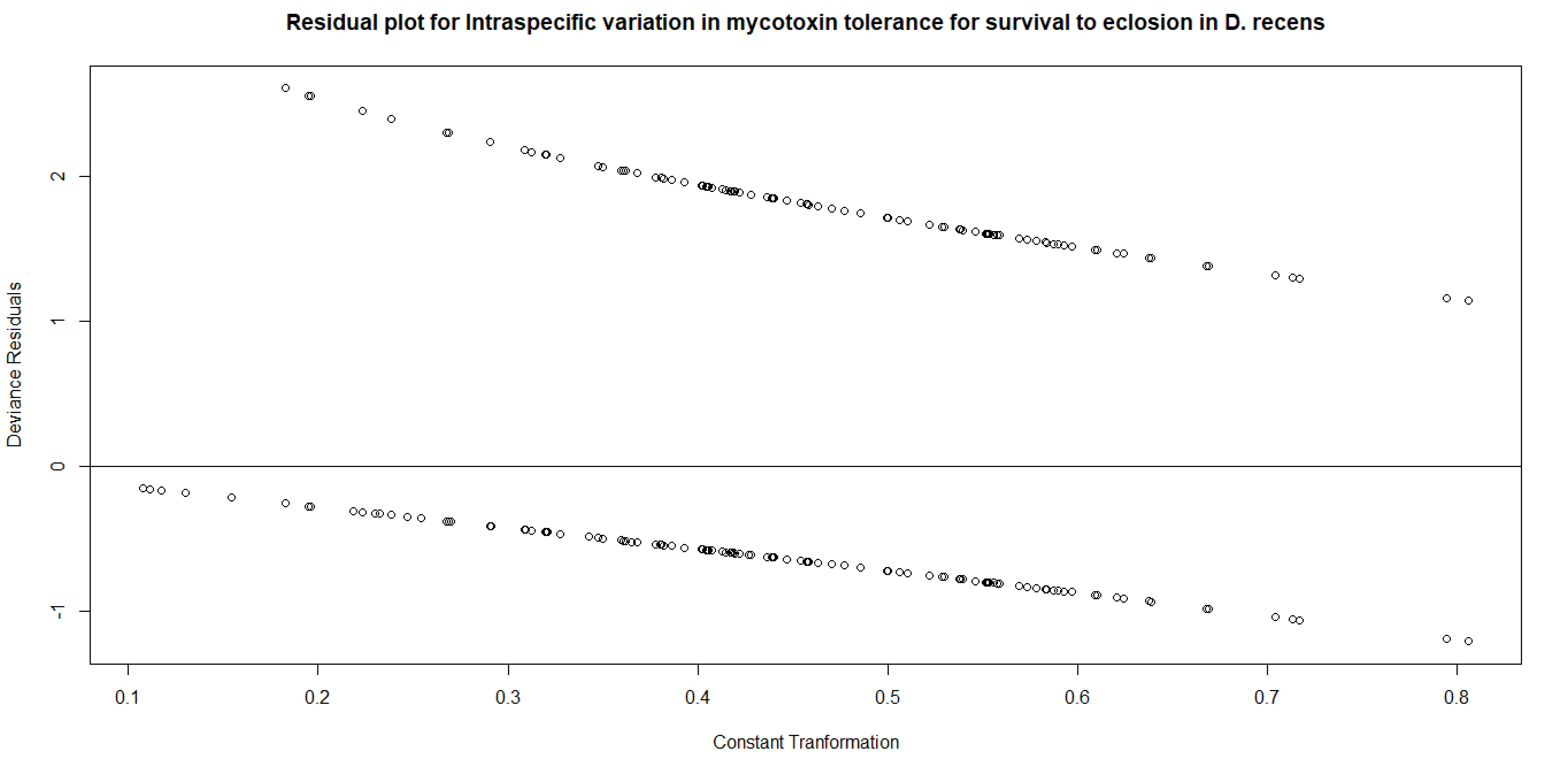
Residual plot for Intraspecific variation in mycotoxin tolerance for survival to eclosion in D. recens
Deviance Residuals
Constant Tranformation
-1
0
1
2
0.1
0.2
0.3
0.4
0.5
0.6
0.7
0.8

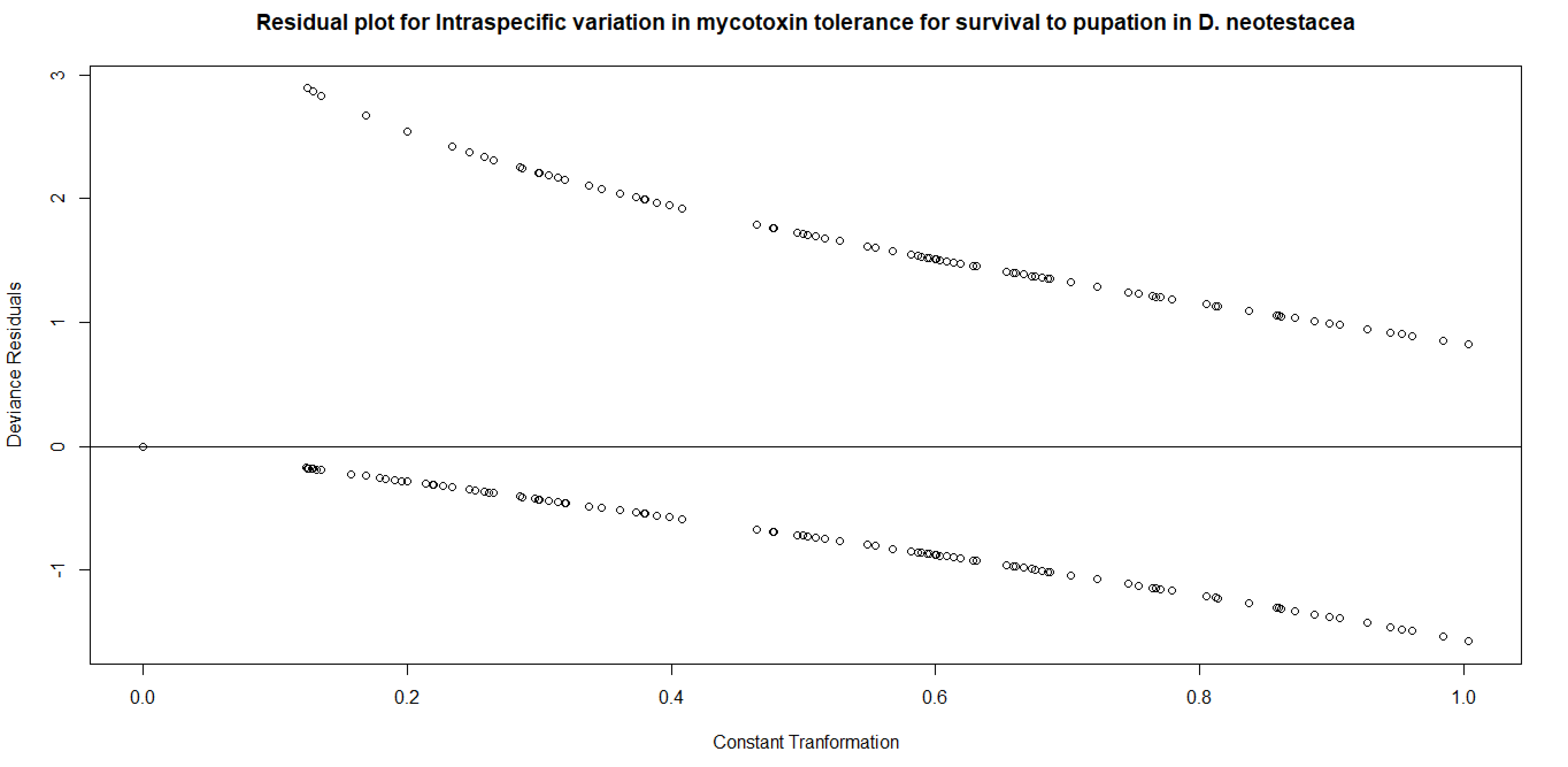
Residual plot for Intraspecific variation in mycotoxin tolerance for survival to pupation in D. neotestacea
Deviance Residuals
Constant Tranformation
3
2
1
0
-1
0.0
0.2
0.4
0.6
0.8
1.0

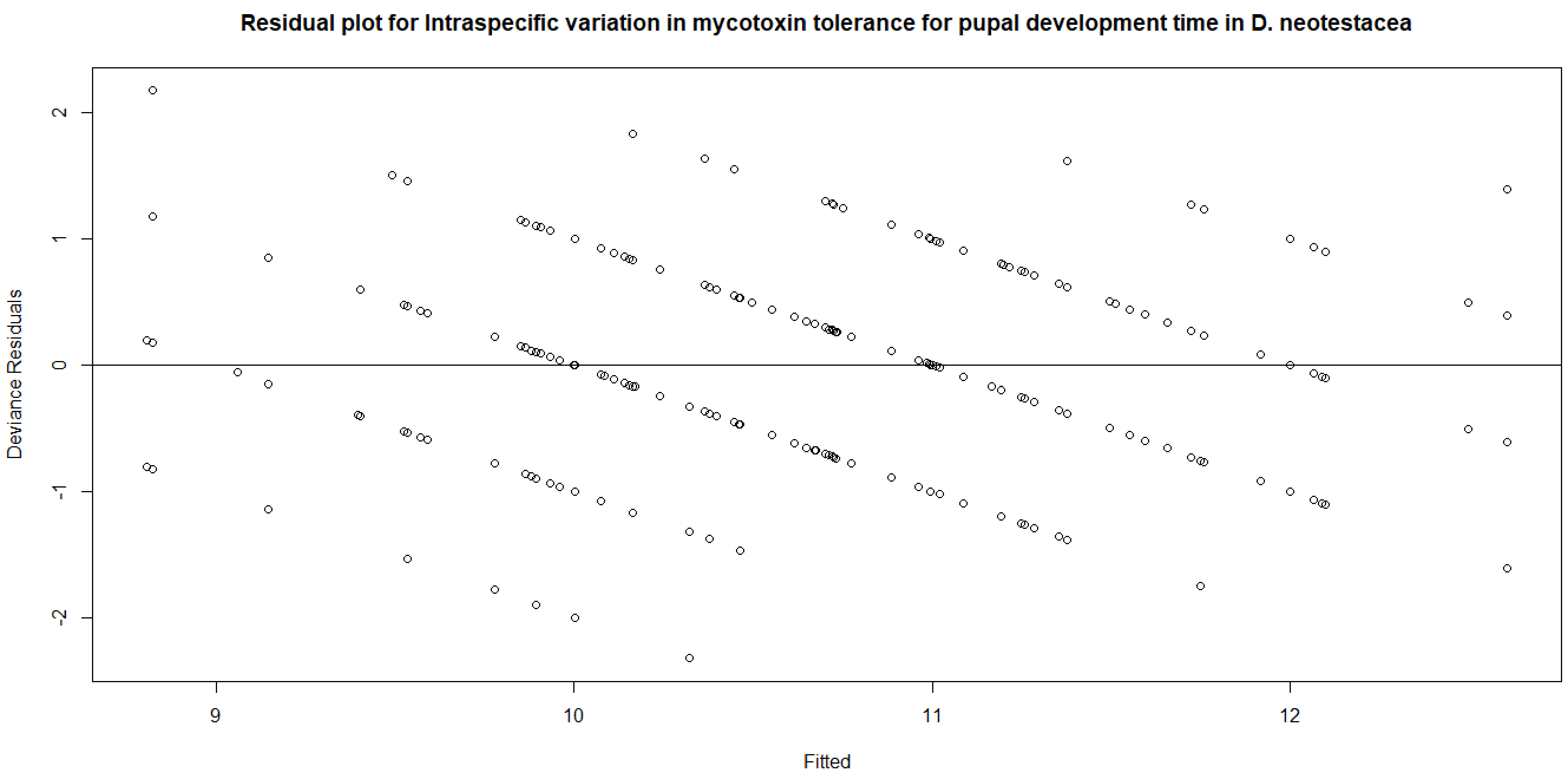
Residual plot for Intraspecific variation in mycotoxin tolerance for pupal development time in D. neotestacea
Deviance Residuals
2
1
0
-1
-2
9
10
11
12
Fitted

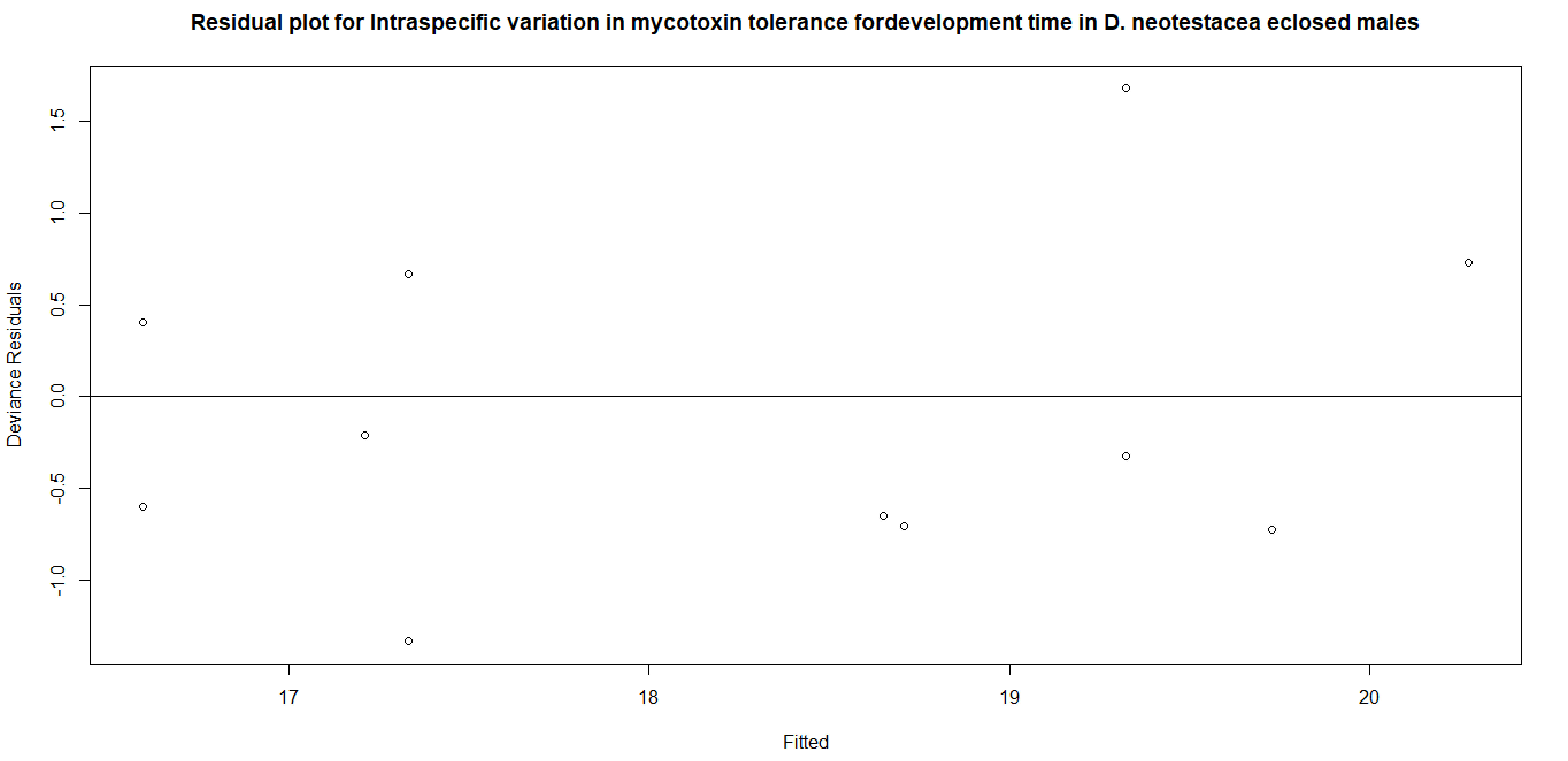
Residual plot for Intraspecific variation in mycotoxin tolerance fordevelopment time in D. neotestacea eclosed males
Deviance Residuals
-1.0
-0.5
0.0
0.5
1.0
1.5
17
18
19
20
Fitted

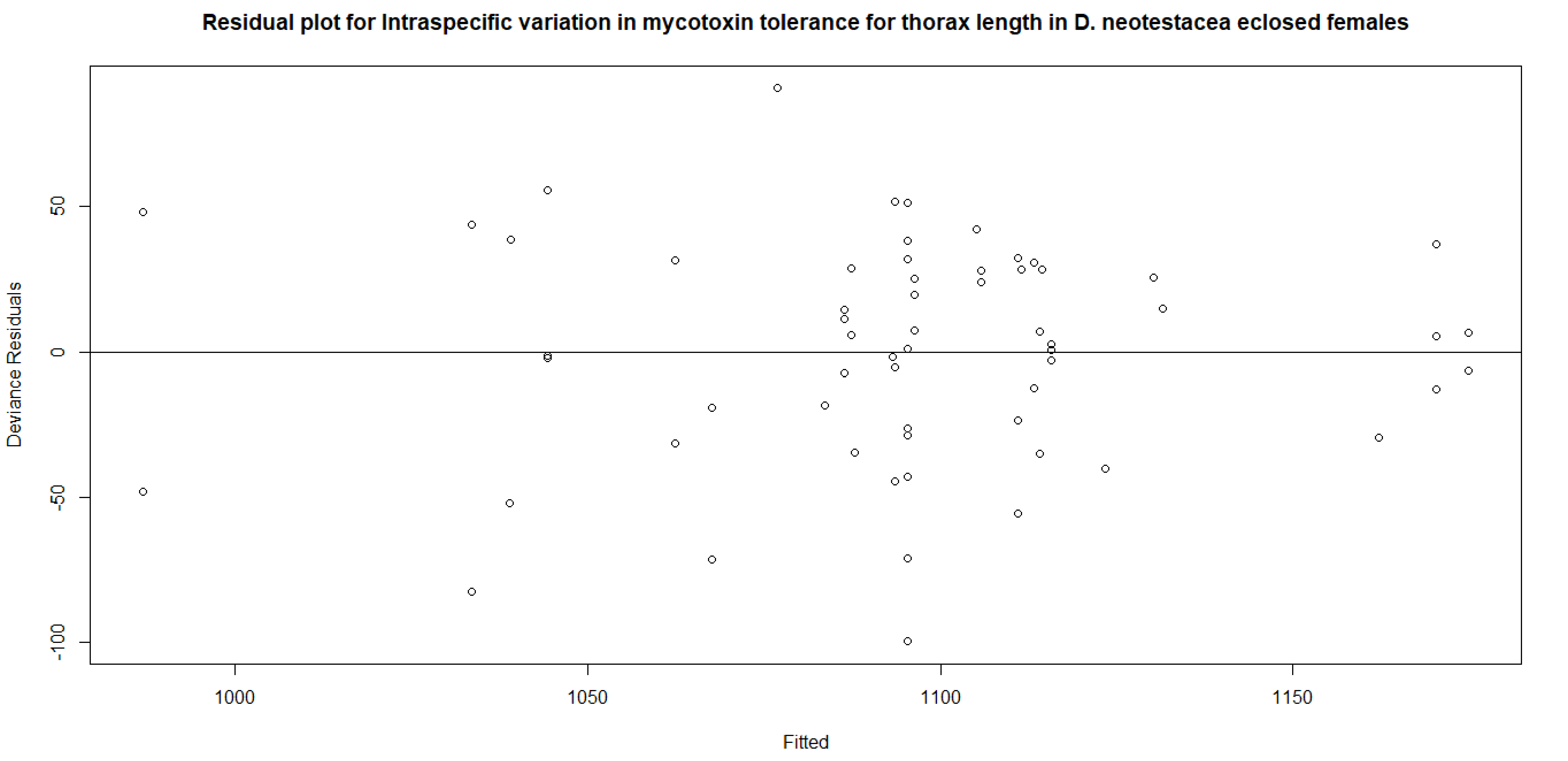
Residual plot for Intraspecific variation in mycotoxin tolerance for thorax length in D. neotestacea eclosed females
Deviance Residuals
50
0
-50
-100
1000
1050
1100
1150
Fitted

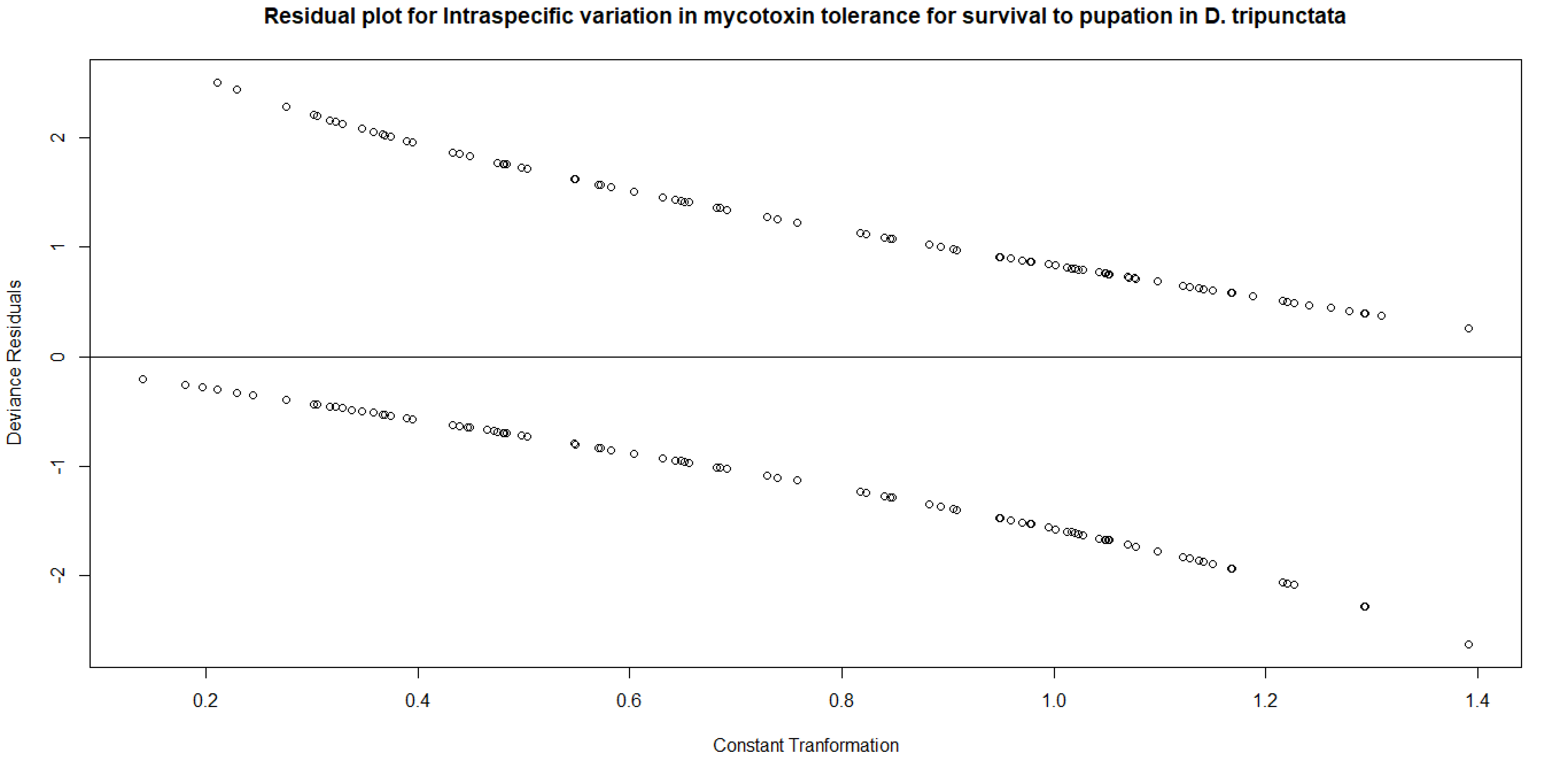
Residual plot for Intraspecific variation in mycotoxin tolerance for survival to pupation in D. tripunctata
Deviance Residuals
2
1
0
-1
-2
0.2
0.4
0.6
0.8
1.0
1.2
1.4
Constant Tranformation

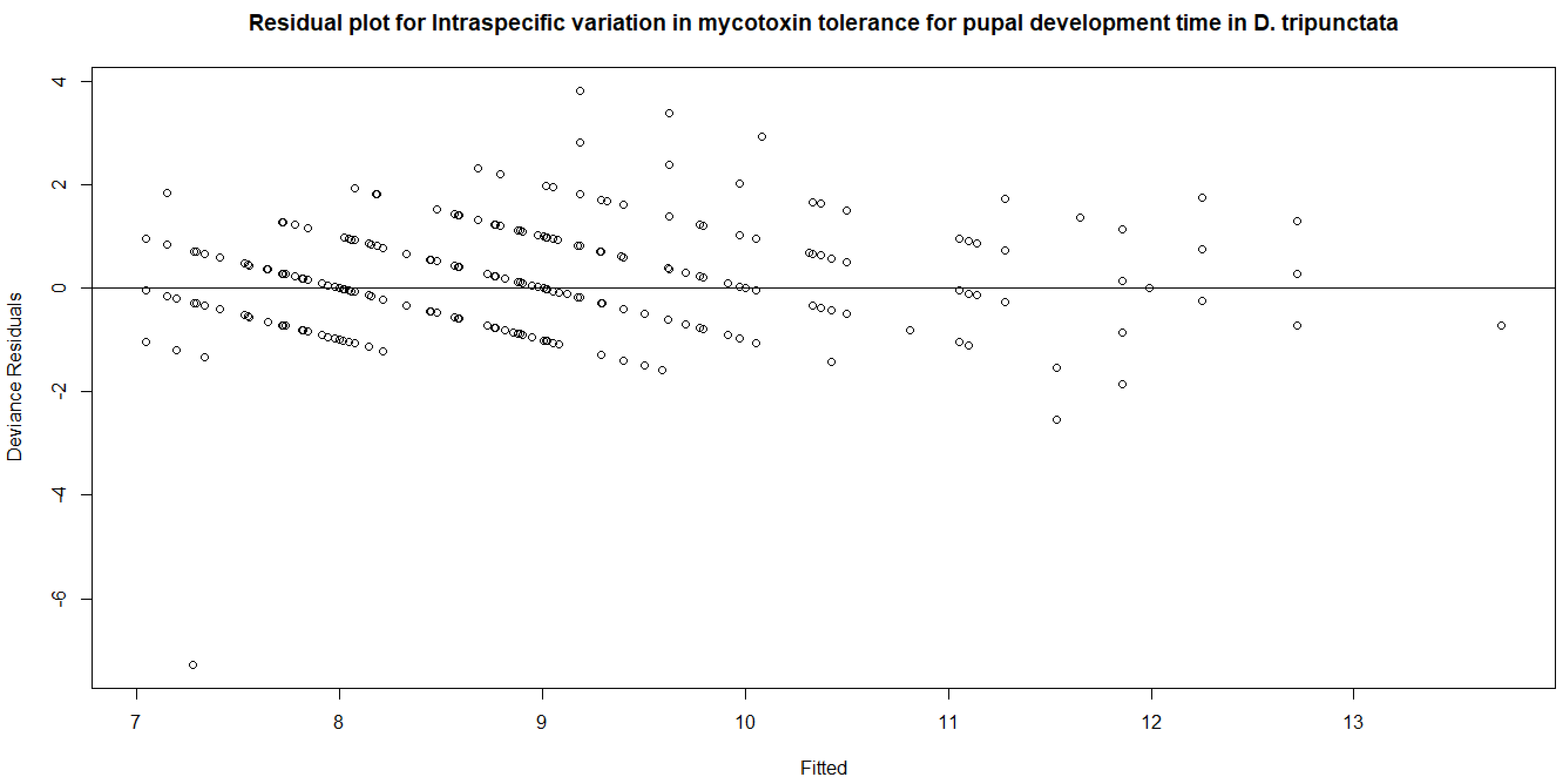
Residual plot for Intraspecific variation in mycotoxin tolerance for pupal development time in D. tripunctata
Deviance Residuals
Fitted
4
2
0
-2
-4
-6
7
8
9
10
11
12
13

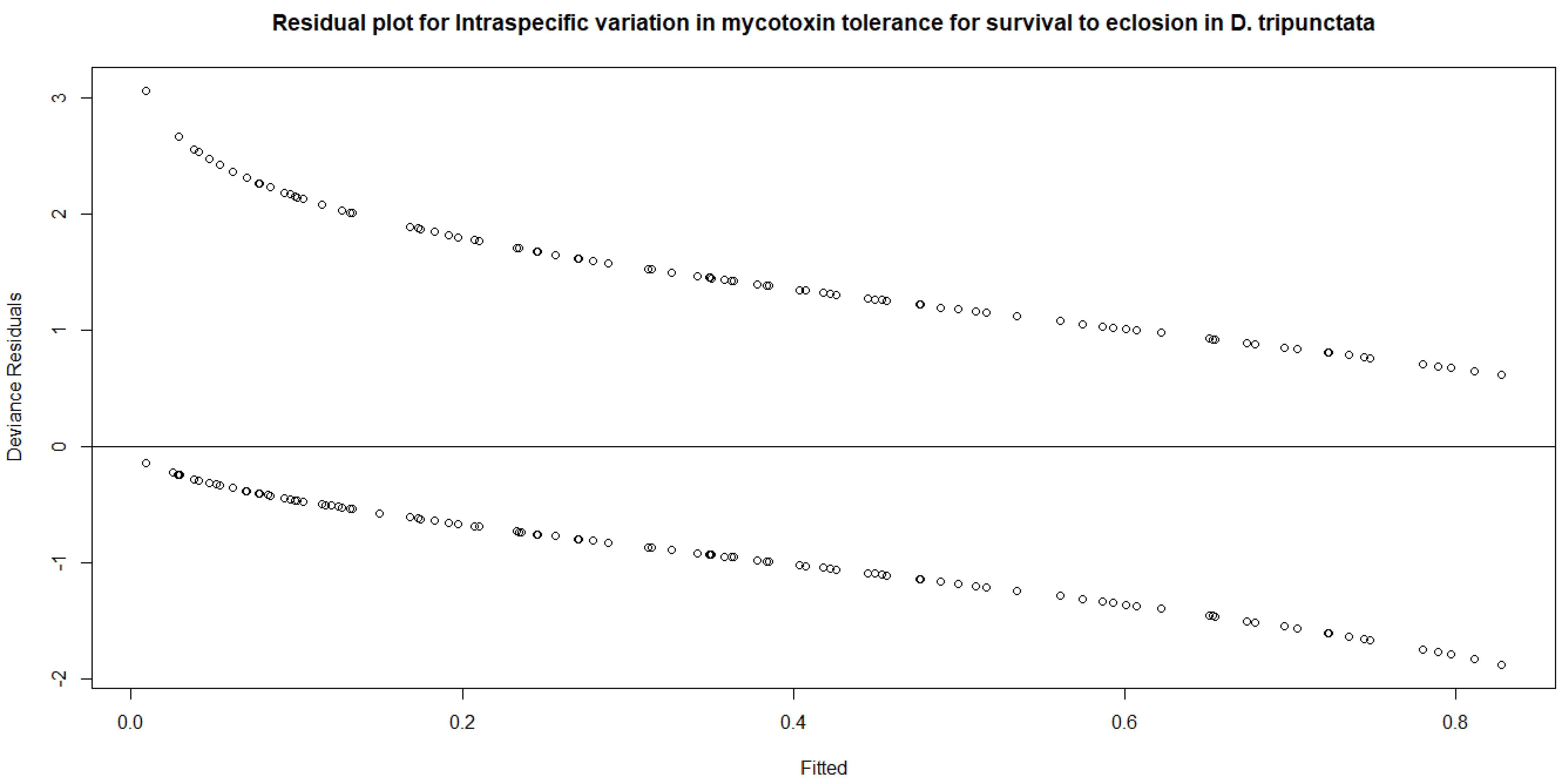
Residual plot for Intraspecific variation in mycotoxin tolerance for survival to eclosion in D. tripunctata
Deviance Residuals
Fitted
3
2
1
0
-1
-2
0.0
0.2
0.4
0.6
0.8

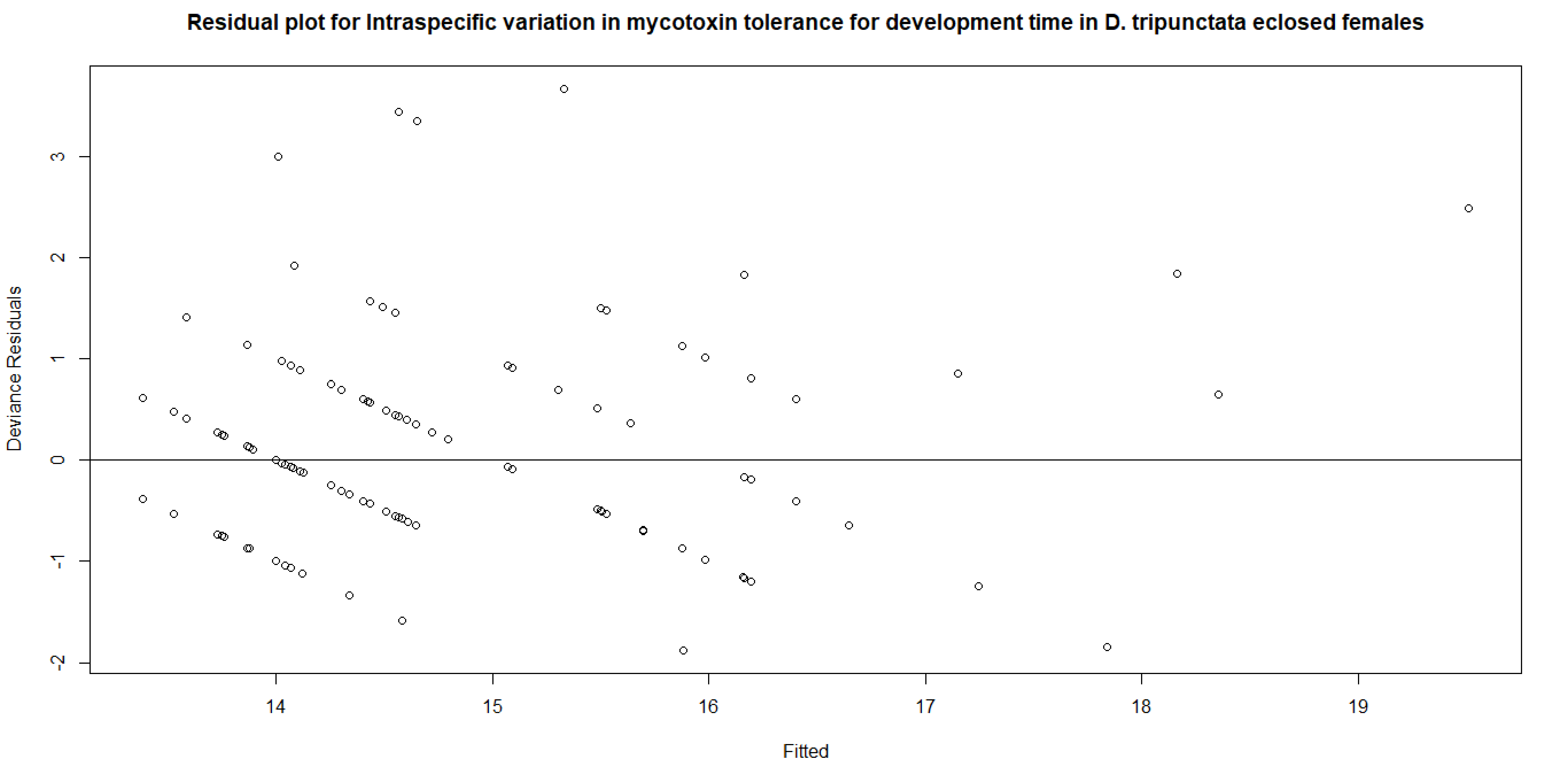

Residual plot for Intraspecific variation in mycotoxin tolerance for development time in D. tripunctata eclosed females
Deviance Residuals
Fitted
-2
-1
0
1
2
3
14
15
16
17
18
19

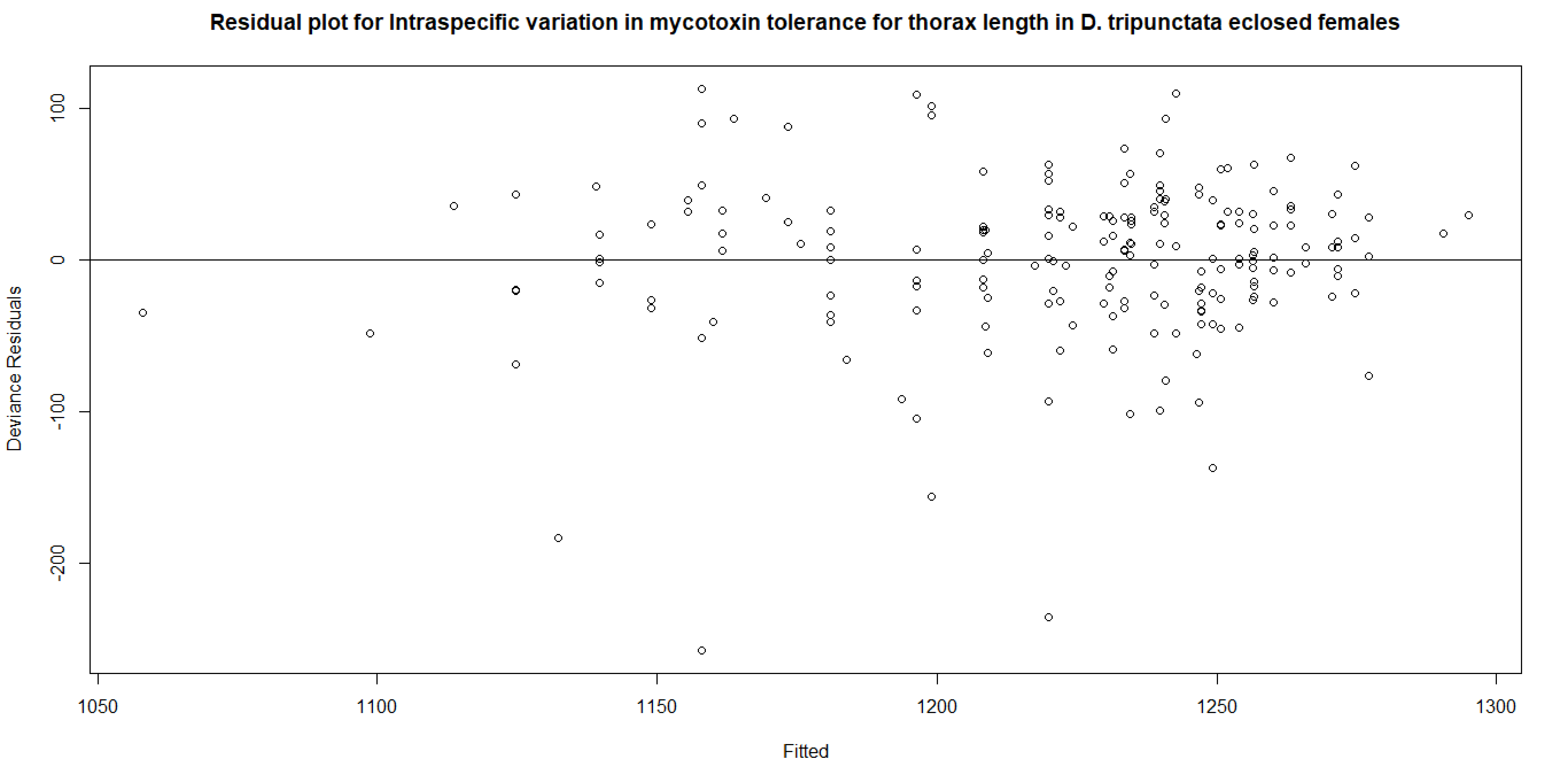
Residual plot for Intraspecific variation in mycotoxin tolerance for thorax length in D. tripunctata eclosed females
Deviance Residuals
100
0
-100
-200
1050
1100
1150
1200
1250
1300
Fitted

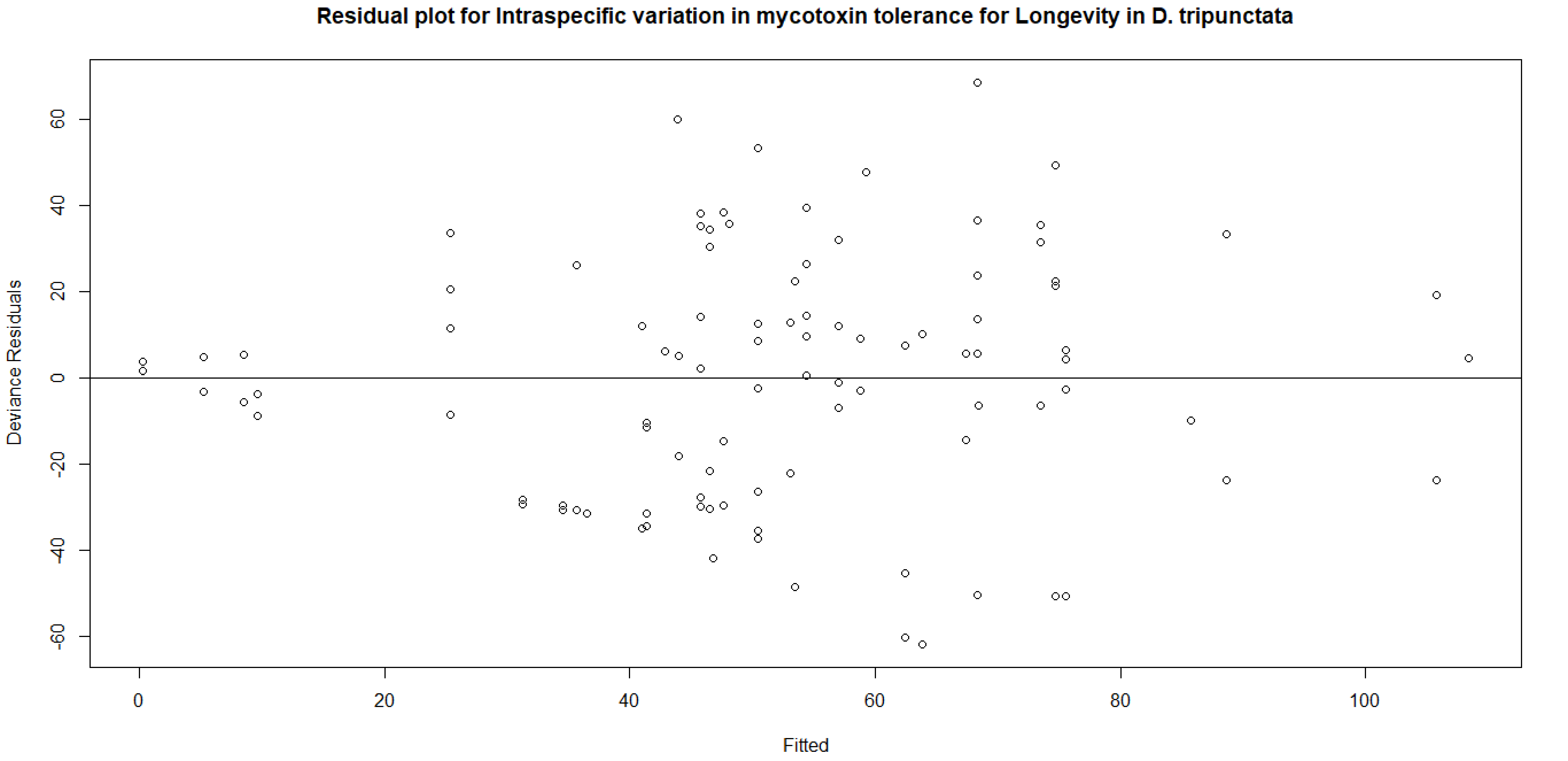

Residual plot for Intraspecific variation in mycotoxin tolerance for Longevity in D. tripunctata
Deviance Residuals
Fitted
60
40
20
0
-20
-40
-60
0
20
40
60
80
100

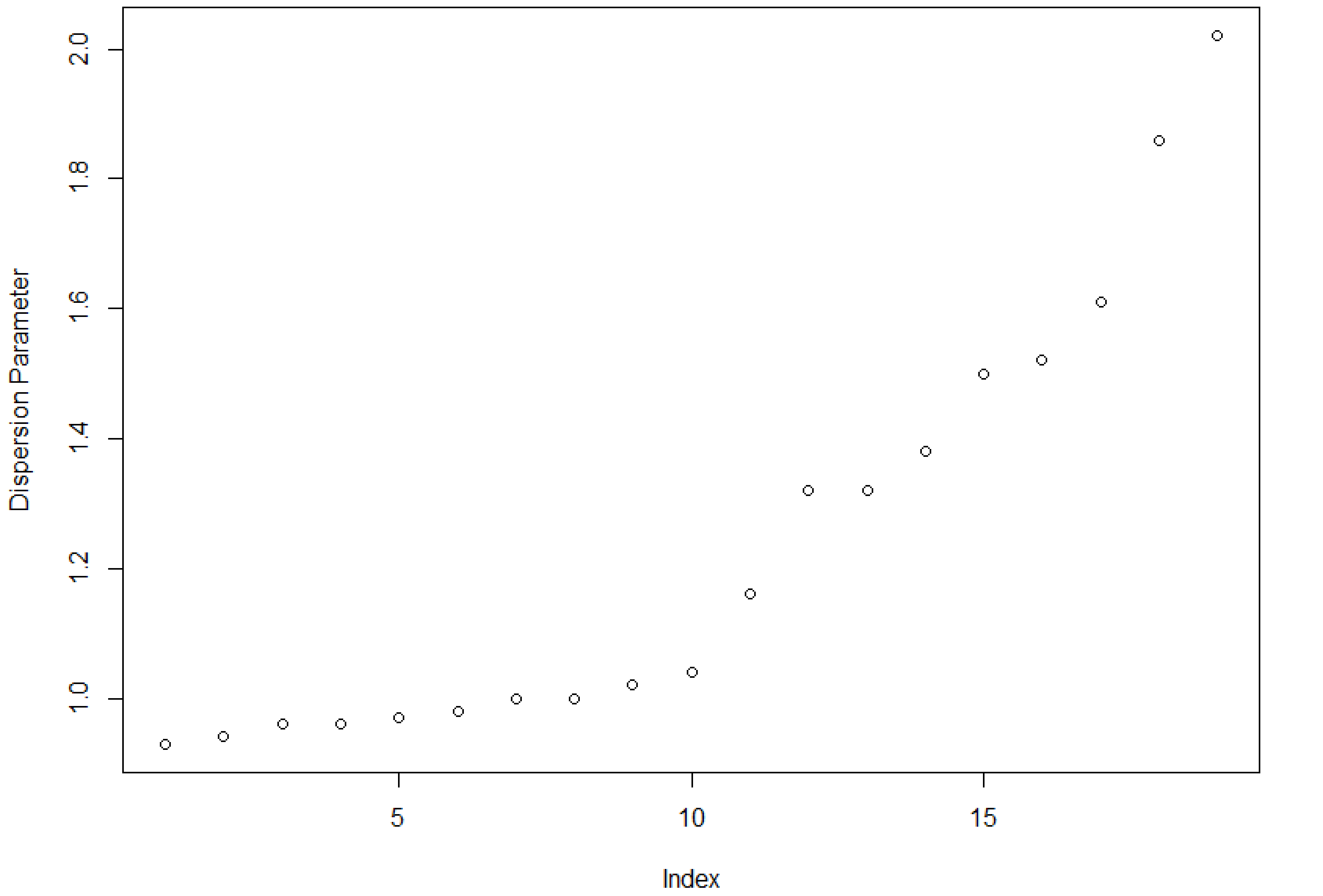

Dispersion Parameter
2.0
1.8
1.6
1.4
1.2
1.0
5
10
15
Index

Table S1

	Contrast	Treatment	Estimate (SE)	95% CI
1	D.falleni - D	Control	-0.496 (0.190)	(-0.983, -0.009)
2	D.falleni - D	Control	-0.309 (0.192)	(-0.804, 0.185)
3	D.falleni - D	Control	-1.869 (0.196)	(-2.374, -1.365)
4	D.neotesta	Control	0.187 (0.187)	(-0.294, 0.668)
5	D.neotesta	Control	-1.373 (0.191)	(-1.864, -0.882)
6	D.recens - I	Control	-1.560 (0.194)	(-2.059, -1.061)
7	D.falleni - D	Mycotoxin	1.576 (0.209)	(1.038, 2.114)
8	D.falleni - D	Mycotoxin	0.019 (0.195)	(-0.483, 0.520)
9	D.falleni - D	Mycotoxin	0.428 (0.200)	(-0.085, 0.940)
10	D.neotesta	Mycotoxin	-1.558 (0.208)	(-2.093, -1.023)
11	D.neotesta	Mycotoxin	-1.148 (0.213)	(-1.695, -0.602)
12	D.recens - I	Mycotoxin	0.409 (0.198)	(-0.101, 0.919)

Table S2

	Contrast	Treatment	Estimate (SE)	95% CI
1	D.falleni - D	Control	0.303 (0.193)	(-0.192, 0.798)
2	D.falleni - D	Control	-0.216 (0.191)	(-0.707, 0.275)
3	D.falleni - D	Control	-1.531 (0.188)	(-2.015, -1.048)
4	D.neotesta	Control	-0.519 (0.189)	(-1.005, -0.033)
5	D.neotesta	Control	-1.834 (0.186)	(-2.313, -1.356)
6	D.recens - I	Control	-1.316 (0.184)	(-1.788, -0.843)
7	D.falleni - D	Mycotoxin	1.613 (0.230)	(1.021, 2.204)
8	D.falleni - D	Mycotoxin	0.188 (0.201)	(-0.328, 0.703)
9	D.falleni - D	Mycotoxin	0.116 (0.202)	(-0.401, 0.634)
10	D.neotesta	Mycotoxin	-1.425 (0.231)	(-2.018, -0.832)
11	D.neotesta	Mycotoxin	-1.496 (0.232)	(-2.092, -0.900)
12	D.recens - I	Mycotoxin	-0.071 (0.203)	(-0.592, 0.449)

Table S3

Species	Trait	P	Bonferroni	BH
D. falleni	Pupal Survival (Number)	0.212385	1	0.318578
D. falleni	Fly Survival (Number)	0.085801	1	0.16257
D. falleni	Pupal Development time (days)	0.000202	0.007267	0.000908
D. falleni	Thorax Length of eclosed males (µm)	0.526653	1	0.631983
D. falleni	Development time of eclosed males (days)	0.241678	1	0.348016
D. falleni	Thorax Length of eclosed females (µm)	0.817986	1	0.936365
D. falleni	Development time of eclosed females (days)	0.861336	1	0.936365
D. falleni	Longevity (days)	0.31194	1	0.431917
D. falleni	Fecundity (Number of offspring)	0.994897	1	0.994897
D. recens	Pupal Survival (Number)	1.20E-05	0.000433	6.19E-05
D. recens	Fly Survival (Number)	2.14E-06	7.71E-05	1.29E-05
D. recens	Pupal Development time (days)	0.135865	1	0.216651
D. recens	Thorax Length of eclosed males (µm)	0.043591	1	0.087182
D. recens	Development time of eclosed males (days)	0.881748	1	0.936365
D. recens	Thorax Length of eclosed females (µm)	0.102399	1	0.184318
D. recens	Development time of eclosed females (days)	0.910355	1	0.936365
D. recens	Longevity (days)	0.402604	1	0.499784
D. recens	Fecundity (Number of offspring)	0.041689	1	0.087182
D. neotestecea	Pupal Survival (Number)	1.09E-69	< 2e-16	< 2e-16
D. neotestecea	Fly Survival (Number)	0	< 2e-16	< 2e-16
D. neotestecea	Pupal Development time (days)	1.16E-12	4.16E-11	8.33E-12
D. neotestecea	Thorax Length of eclosed males (µm)	0.138416	1	0.216651
D. neotestecea	Development time of eclosed males (days)	0.027905	1	0.071755
D. neotestecea	Thorax Length of eclosed females (µm)	0.909302	1	0.936365
D. neotestecea	Development time of eclosed females (days)	0.000577	0.020761	0.002076
D. neotestecea	Longevity (days)	0.035355	1	0.084852
D. neotestecea	Fecundity (Number of offspring)	0.3303	1	0.4404
D. tripunctata	Pupal Survival (Number)	1.88E-87	< 2e-16	< 2e-16
D. tripunctata	Fly Survival (Number)	1.42E-51	< 2e-16	< 2e-16
D. tripunctata	Pupal Development time (days)	0.017344	0.624394	0.04803
D. tripunctata	Thorax Length of eclosed males (µm)	0.003186	0.114714	0.009559
D. tripunctata	Development time of eclosed males (days)	0.000362	0.013038	0.001449
D. tripunctata	Thorax Length of eclosed females (µm)	0.001515	0.054556	0.00496
D. tripunctata	Development time of eclosed females (days)	0.037953	1	0.085394
D. tripunctata	Longevity (days)	0.360562	1	0.46358
D. tripunctata	Fecundity (Number of offspring)	0.117213	1	0.200937

Table S3 (part 2)

Mean +/- SD (N)		contrasts	Estimate (SE)	95% CI (Lower, Upper)
Control	Mycotoxin			
4.9 +/- 3.42 (294)	4.53 +/- 3.3 (270)	Control - Mycotoxin	0.215 (0.125)	(-0.030, 0.460)
2.95 +/- 2.51 (177)	2.5 +/- 2.06 (150)	Control - Mycotoxin	0.134 (0.107)	(0.134, 0.107)
9.16 +/- 1.11 (289)	9.5 +/- 1.12 (264)	Control - Mycotoxin	-0.305 (0.082)	(-0.305, 0.082)
1061 +/- 62.2 (62)	1065 +/- 72.6 (55)	Control - Mycotoxin	-7.741 (12.566)	(-7.741, 12.566)
15.9 +/- 1.57 (62)	16.2 +/- 1.5 (55)	Control - Mycotoxin	-0.295 (0.257)	(-0.295, 0.257)
1201 +/- 72.4 (99)	1188 +/- 88.1 (82)	Control - Mycotoxin	2.518 (11.160)	(2.518, 11.160)
15.6 +/- 1.77 (99)	15.6 +/- 1.5 (82)	Control - Mycotoxin	0.038 (0.222)	(0.038, 0.222)
47 +/- 27.1 (63)	40.9 +/- 29.1 (43)	Control - Mycotoxin	5.212 (5.301)	(5.212, 5.301)
122 +/- 99 (61)	125 +/- 88.9 (54)	Control - Mycotoxin	0.115 (18.560)	(0.115, 18.560)
5.94 +/- 3.67 (374)	4.63 +/- 3.15 (292)	Control - Mycotoxin	0.458 (0.105)	(0.458, 0.105)
3.37 +/- 2.49 (215)	2.19 +/- 2.31 (138)	Control - Mycotoxin	0.583 (0.123)	(0.583, 0.123)
9.88 +/- 1.51 (373)	9.83 +/- 1.39 (292)	Control - Mycotoxin	0.132 (0.089)	(0.132, 0.089)
1067 +/- 66.1 (86)	1.48 +/- 64.1 (55)	Control - Mycotoxin	22.576 (11.519)	(22.576, 11.519)
16.4 +/- 1.97 (86)	16.3 +/- 1.80 (55)	Control - Mycotoxin	-0.042 (0.285)	(-0.042, 0.285)
1135 +/- 75.4 (104)	1118 +/- 73.4 (65)	Control - Mycotoxin	18.617 (11.619)	(18.617, 11.619)
15.8 +/- 1.81 (104)	15.7 +/- 1.87 (65)	Control - Mycotoxin	-0.030 (0.269)	(-0.030, 0.269)
58.8 +/- 33.9 (82)	64.8 +/- 35.9 (54)	Control - Mycotoxin	-5.124 (6.416)	(-5.124, 6.416)
82.4 +/- 58.8 (59)	106 +/- 65.5 (38)	Control - Mycotoxin	-24.631 (12.648)	(-24.631, 12.648)
8.53 +/- 3.75 (512)	1.82 +/- 2.43 (109)	Control - Mycotoxin	2.135 (0.121)	(2.135, 0.121)
3.05 +/- 2.61 (183)	0.8 +/- 1.13 (48)	Control - Mycotoxin	1.496 (0.002)	(1.496, 0.002)
10.4 +/- 1.03 (512)	11 +/- 1.16 (109)	Control - Mycotoxin	-0.625 (0.088)	(-0.625, 0.088)
953 +/- 55.3 (77)	943 +/- 54.8 (16)	Control - Mycotoxin	22.466 (15.723)	(22.466, 15.723)
17.7 +/- 1.73 (77)	18.6 +/- 1.41 (16)	Control - Mycotoxin	-1.021 (0.484)	(-1.021, 0.484)
1085 +/- 56.7 (92)	1088 +/- 63.4 (29)	Control - Mycotoxin	1.429 (12.990)	(1.429, 12.990)
17.7 +/- 1.69 (92)	17.9 +/- 1.7 (29)	Control - Mycotoxin	-1.200 (0.359)	(-1.200, 0.359)
51.6 +/- 31.4 (77)	28.4 +/- 26.8 (14)	Control - Mycotoxin	18.800 (9.307)	(18.800, 9.307)
75.4 +/- 81.7 (52)	85 +/- 86.6 (10)	Control - Mycotoxin	-23.867 (25.678)	(-23.867, 25.678)
10.7 +/- 3.26 (643)	3.38 +/- 3.41 (203)	Control - Mycotoxin	2.437 (0.123)	(2.437, 0.123)
7.4 +/- 3.8 (444)	2.38 +/- 3.03 (143)	Control - Mycotoxin	1.891 (0.125)	(1.891, 0.125)
8.91 +/- 1.3 (643)	8.87 +/- 1.46 (203)	Control - Mycotoxin	-0.207 (0.087)	(-0.207, 0.087)
1070 +/- 56.5 (146)	1052 +/- 64.8 (39)	Control - Mycotoxin	27.516 (9.452)	(27.516, 9.452)
14.6 +/- 1.47 (146)	15 +/- 1.45 (39)	Control - Mycotoxin	-0.591 (0.166)	(-0.591, 0.166)
1223 +/- 78.7 (170)	1204 +/- 74.6 (71)	Control - Mycotoxin	33.258 (10.642)	(33.258, 10.642)
14.9 +/- 1.48 (170)	14.9 +/- 1.87 (71)	Control - Mycotoxin	-0.391 (0.190)	(-0.391, 0.190)
44.9 +/- 34.5 (100)	52.1 +/- 40.8 (36)	Control - Mycotoxin	-5.751 (6.377)	(-5.751, 6.377)
291 +/- 151 (143)	245 +/- 110 (37)	Control - Mycotoxin	46.196 (30.832)	(46.196, 30.832)

Table S4: Table showing the mycotoxin tolerance status of isofemale lines for each species and location.

Species	Location	No. of isofemale lines with low tolerance	No. of isofemale lines with high tolerance
D. falleni	GSM	0	10
	ESC	2	8
D. recens	GSM	3	7
	ESC	4	6
D. neostestacea	GSM	7	3
	ESC	5	5
D. tripunctata	GSM	8	2
	ESC	7	3

Table S5

Trait	Effect	Chi-square	Degrees of Free	p-value (original)	p-value (Bonferro	FDR (BH)
Pupal Survival	Treatment	0.0005274	1	9.82E-01	1	0.99963
Pupal Survival	Location	2.8478403	1	0.091496774	1	0.25888
Pupal Survival	Isofemale_line	21.7763474	18	0.242066154	1	0.50251
Pupal Survival	Treatment:Location	0.08764981	1	0.767186475	1	0.93872
**Pupal Survival**	**Treatment:Isofemale_line**	**60.3401785**	**18**	**1.80E-06**	**8.10E-05**	**4.05E-05**
Pupal Development time	Treatment	5.41884898	1	0.019920488	0.89642	0.08964
Pupal Development time	Location	7.0248545	1	0.0080386	0.36174	0.04554
Pupal Development time	Isofemale_line	29.2411242	18	0.045510648	1	0.15754
Pupal Development time	Treatment:Location	14.1054612	1	0.000172841	0.00778	0.00259
**Pupal Development time**	**Treatment:Isofemale_line**	**111.419977**	**18**	**1.71E-15**	**7.70E-14**	**7.70E-14**
Fly Survival	Treatment	0.96520932	1	0.325877875	1	0.54313
Fly Survival	Location	4.24719464	1	0.039315225	1	0.14743
Fly Survival	Isofemale_line	40.0416189	18	0.002060196	0.09271	0.01854
Fly Survival	Treatment:Location	0.33566656	1	0.562341307	1	0.79079
**Fly Survival**	**Treatment:Isofemale_line**	**42.9687044**	**18**	**0.000808421**	**0.03638**	**0.00909**
Development time of eclosed males	Treatment	2.32151224	1	0.127595927	1	0.3022
Development time of eclosed males	Location	0.04386846	1	0.834098538	1	0.98775
Development time of eclosed males	Isofemale_line	3.69557983	9	0.930282966	1	0.99963
Development time of eclosed males	Treatment:Location	5.22206432	1	0.022302061	1	0.09124
**Development time of eclosed males**	**Treatment:Isofemale_line**	**21.543247**	**9**	**0.01044493**	**0.47002**	**0.05222**
Thorax length of eclosed males	Treatment	0.87321652	1	0.350066375	1	0.56261
Thorax length of eclosed males	Location	1.06934606	1	0.301093201	1	0.54197
Thorax length of eclosed males	Isofemale_line	3.617272	9	0.93475223	1	0.99963
Thorax length of eclosed males	Treatment:Location	3.26407988	1	0.070812738	1	0.22761
**Thorax length of eclosed males**	**Treatment:Isofemale_line**	**22.2582732**	**9**	**0.008095499**	**0.3643**	**0.04554**
**Development time of eclosed females**	Treatment	0.02669129	1	0.870223431	1	0.99963
**Development time of eclosed females**	Location	0.39565386	1	0.529342363	1	0.7684
**Development time of eclosed females**	Isofemale_line	3.94674671	11	0.97144896	1	0.99963
**Development time of eclosed females**	Treatment:Location	1.1719122	1	0.279008684	1	0.52314
**Development time of eclosed females**	Treatment:Isofemale_line	16.6332086	11	0.119203065	1	0.29801
**Thorax length of eclosed females**	Treatment	0.99132321	1	0.319419185	1	0.54313
**Thorax length of eclosed females**	Location	0.22457825	1	0.635573445	1	0.86669

Table S5 (part 2)

**Thorax length of eclosed females**	Isofemale_line	1.49308622	11	0.999628096	1	0.99963
**Thorax length of eclosed females**	Treatment:Location	1.34774513	1	0.245672703	1	0.50251
**Thorax length of eclosed females**	Treatment:Isofemale_line	16.033824	11	0.139879814	1	0.31473
Longevity	Treatment	2.83821315	1	0.09204652	1	0.25888
Longevity	Location	0.14040914	1	0.707874607	1	0.92608
Longevity	Isofemale_line	6.19470465	9	0.720284348	1	0.92608
Longevity	Treatment:Location	0.74891239	1	0.386820795	1	0.60024
**Longevity**	**Treatment:Isofemale_line**	**22.4374839**	**9**	**0.007591191**	**0.3416**	**0.04554**
Fecundity	Treatment	1.19988467	1	0.27334473	1	0.52314
Fecundity	Location	0.0840835	1	0.771838007	1	0.93872
Fecundity	Isofemale_line	1.80839176	7	0.96968053	1	0.99963
Fecundity	Treatment:Location	2.59607623	1	0.107128647	1	0.28358
Fecundity	Treatment:Isofemale_line	7.05114864	7	0.423573799	1	0.63536

Table S6

Trait	Effect	Chi-square St	Degrees of Free	p-value (original	p-value (Bonferroi	FDR (BH)
Pupal Survival	Treatment	0.43125006	1	0.511376198	1	0.954353
Pupal Survival	Location	0.76388203	1	0.382116374	1	0.954353
Pupal Survival	Isofemale_line	43.8128318	18	0.000613509	0.02761	0.008656
Pupal Survival	Treatment:Location	5.50664138	1	0.018944392	0.8525	0.106562
**Pupal Survival**	**Treatment:Isofemale_line**	**52.7620824**	**18**	**2.86E-05**	**0.00128**	**0.000642**
Pupal Development time	Treatment	0.47533458	1	0.490543194	1	0.954353
Pupal Development time	Location	0.10001424	1	0.751812547	1	0.999978
Pupal Development time	Isofemale_line	4.02911007	18	0.99974977	1	0.999978
Pupal Development time	Treatment:Location	1.76717512	1	0.183732067	1	0.590567
**Pupal Development time**	**Treatment:Isofemale_line**	**93.9570892**	**18**	**2.79E-12**	**1.26E-10**	**1.26E-10**
Fly Survival	Treatment	3.76E-05	1	9.95E-01	1	0.999978
Fly Survival	Location	0.23040501	1	0.631223683	1	0.985029
Fly Survival	Isofemale_line	18.7193941	18	4.09E-01	1	0.954353
Fly Survival	Treatment:Location	6.87576033	1	0.008737258	0.39318	0.078635
**Fly Survival**	**Treatment:Isofemale_line**	**43.1207391**	**18**	**7.69E-04**	**0.03462**	**0.008656**
Development time of eclosed males	Treatment	0.39401595	1	0.530195967	1	0.954353
Development time of eclosed males	Location	0.00173763	1	0.966749878	1	0.999978
Development time of eclosed males	Isofemale_line	1.61587723	14	0.99997792	1	0.999978
Development time of eclosed males	Treatment:Location	0.22561241	1	0.634796417	1	0.985029
Development time of eclosed males	Treatment:Isofemale_line	26.4311116	13	0.01487088	0.66919	0.106562
Thorax length of eclosed males	Treatment	4.80446928	1	0.028386009	1	0.14193
Thorax length of eclosed males	Location	0.72212058	1	0.395449201	1	0.954353
Thorax length of eclosed males	Isofemale_line	3.04794904	14	0.998986283	1	0.999978
Thorax length of eclosed males	Treatment:Location	1.44679096	1	0.229043569	1	0.663729
Thorax length of eclosed males	Treatment:Isofemale_line	6.22253106	13	0.937677475	1	0.999978
Development time of eclosed females	Treatment	1.40437342	1	0.235992689	1	0.663729
Development time of eclosed females	Location	0.02646138	1	0.870778623	1	0.999978
Development time of eclosed females	Isofemale_line	4.88506296	9	0.844210259	1	0.999978
Development time of eclosed females	Treatment:Location	3.06280394	1	0.080103331	1	0.300387
**Development time of eclosed females**	**Treatment:Isofemale_line**	20.0519254	9	0.017594644	**0.79176**	**0.106562**
Thorax length of eclosed females	Treatment	3.69834857	1	0.054466351	1	0.222817
Thorax length of eclosed females	Location	0.29577726	1	0.586541888	1	0.97757

Table S6 (part 2)

Thorax length of eclosed females	Isofemale_line	8.54136744	9	0.480639104	1	0.954353
Thorax length of eclosed females	Treatment:Location	0.14301619	1	0.705300736	1	0.999978
**Thorax length of eclosed females**	**Treatment:Isofemale_line**	18.2666249	9	0.03220324	**1**	**0.144915**
Longevity	Treatment	0.01379778	1	0.906492412	1	0.999978
Longevity	Location	0.05264819	1	0.818517673	1	0.999978
Longevity	Isofemale_line	4.17836231	11	0.964405862	1	0.999978
Longevity	Treatment:Location	0.02063299	1	0.885783277	1	0.999978
Longevity	Treatment:Isofemale_line	16.6564109	11	0.118457665	1	0.410046
Fecundity	Treatment	0.4277388	1	0.513100576	1	0.954353
Fecundity	Location	0.3137595	1	0.575382319	1	0.97757
Fecundity	Isofemale_line	1.6843916	8	0.989207021	1	0.999978
Fecundity	Treatment:Location	0.56304655	1	0.453035338	1	0.954353
Fecundity	Treatment:Isofemale_line	4.670939	8	0.792097604	1	0.999978

Table S7

Trait	Effect	Chi-square Statistics
Pupal Survival	Treatment	23.3735353
Pupal Survival	Location	8.846780867
Pupal Survival	Isofemale_line	39.08227883
Pupal Survival	Treatment:Location	4.09E-05
**Pupal Survival**	**Treatment:Isofemale_line**	**64.04958479**
Pupal Development time	Treatment	12.19825414
Pupal Development time	Location	0.162054412
Pupal Development time	Isofemale_line	3.573057494
Pupal Development time	Treatment:Location	0.121769838
**Pupal Development time**	**Treatment:Isofemale_line**	**95.4514318**
**Fly Survival**	Treatment	0.153821617
**Fly Survival**	Location	1.476558215
**Fly Survival**	Isofemale_line	20.59192339
**Fly Survival**	Treatment:Location	1.456597906
**Fly Survival**	Treatment:Isofemale_line	15.67377604
Development time of eclosed males	Treatment	5.638672394
Development time of eclosed males	Isofemale_line	1.415366563
**Development time of eclosed males**	**Treatment:Isofemale_line**	**12.50258937**
Thorax length of eclosed males	Treatment	3.259547069
Thorax length of eclosed males	Isofemale_line	0.01837851
Thorax length of eclosed males	Treatment:Isofemale_line	3.76708339
**Development time of eclosed females**	Treatment	6.521828465
**Development time of eclosed females**	Location	0.843962021
**Development time of eclosed females**	Isofemale_line	5.066319919
**Development time of eclosed females**	Treatment:Location	1.593427641
**Development time of eclosed females**	Treatment:Isofemale_line	10.85923371
Thorax length of eclosed females	Treatment	4.479716677
Thorax length of eclosed females	Location	1.029516438
Thorax length of eclosed females	Isofemale_line	7.399125751
Thorax length of eclosed females	Treatment:Location	4.129766283
**Thorax length of eclosed females**	**Treatment:Isofemale_line**	**23.17721405**
Longevity	Treatment	2.097549351
Longevity	Isofemale_line	0.380676547
Longevity	Treatment:Isofemale_line	2.345622829
Fecundity	Treatment	4.220864129
Fecundity	Isofemale_line	1.898336553
Fecundity	Treatment:Isofemale_line	3.607405178

Table S7 (part 2)

Degrees of Freedom	p-value (original)	p-value (Bonferroni)	FDR (BH)
1	1.33E-06	4.94E-05	1.65E-05
1	0.002936063	0.1086	0.01358
17	0.00174065	0.0644	0.0092
1	0.994895386	1	0.99976
**17**	**2.22E-07**	8.22E-06	4.11E-06
1	0.000478343	0.0177	0.00354
1	0.687272071	1	0.77914
17	0.999761204	1	0.99976
1	0.727122829	1	0.79128
**16**	**2.45E-13**	9.06E-12	9.06E-12
1	0.694909713	1	0.77914
1	0.224313799	1	0.33324
10	0.024125837	0.8927	0.08115
1	0.227472206	1	0.33324
10	0.109359529	1	0.21296
1	0.017568505	0.65	0.065
1	0.234167552	1	0.33324
**1**	**0.000406388**	0.015	0.00354
1	0.071008737	1	0.15455
1	0.892163307	1	0.94314
1	0.052270702	1	0.12893
1	0.010655839	0.3943	0.04381
1	0.358266084	1	0.4571
6	0.535335705	1	0.64122
1	0.206837102	1	0.33274
6	0.092825638	1	0.19081
1	0.034299407	1	0.10576
1	0.310272269	1	0.41
6	0.285507096	1	0.39125
1	0.04213517	1	0.11136
**6**	**0.000739246**	0.0274	0.00456
1	0.147535439	1	0.25994
1	0.537241382	1	0.64122
1	0.125635339	1	0.23243
1	0.039929821	1	0.11136
1	0.16826463	1	0.28299
1	0.057522805	1	0.13302

Table S8

Trait	Effect	Chi-square	Degrees of	p-value (origin	p-value (Bc	FDR (BH)
Pupal Survival	Treatment	26.70814	1	2.37E-07	1.06E-05	5.06E-06
Pupal Survival	Location	0.198724	1	0.655752537	1	0.983629
Pupal Survival	Isofemale_line	30.94682	17	0.020271799	0.912231	0.069814
Pupal Survival	Treatment:Location	1.148696	1	0.283822309	1	0.608191
**Pupal Survival**	**Treatment:Isofemale_line**	**60.26734**	**17**	**9.49E-07**	4.27E-05	1.07E-05
Pupal Development time	Treatment	1.194529	1	0.274417848	1	0.608191
Pupal Development time	Location	0.083262	1	0.772924575	1	0.999996
Pupal Development time	Isofemale_line	2.055153	17	0.999995767	1	0.999996
Pupal Development time	Treatment:Location	1.736215	1	0.187619088	1	0.469048
**Pupal Development time**	**Treatment:Isofemale_line**	**124.6794**	**17**	**1.97E-18**	< 2e-16	< 2e-16
Fly Survival	Treatment	20.12919	1	7.24E-06	0.000326	6.51E-05
Fly Survival	Location	0.359646	1	0.548702738	1	0.983629
Fly Survival	Isofemale_line	51.59399	16	1.27E-05	0.000573	9.55E-05
Fly Survival	Treatment:Location	0.00342	1	0.953368653	1	0.999996
**Fly Survival**	**Treatment:Isofemale_line**	**49.99147**	**16**	**2.30E-05**	0.001035	0.000148
Development time of eclosed males	Treatment	10.34986	1	0.001294845	0.058268	0.006474
Development time of eclosed males	Location	0.013895	1	0.906164832	1	0.999996
Development time of eclosed males	Isofemale_line	0.652004	6	0.995468879	1	0.999996
Development time of eclosed males	Treatment:Location	6.97425	1	0.008269087	0.372109	0.031009
Development time of eclosed males	Treatment:Isofemale_line	13.68708	6	0.033333872	1	0.100002
Thorax length of eclosed males	Treatment	1.37554	1	0.240862388	1	0.570464
Thorax length of eclosed males	Location	0.669377	1	0.41326889	1	0.821052
Thorax length of eclosed males	Isofemale_line	1.537498	6	0.95696489	1	0.999996
Thorax length of eclosed males	Treatment:Location	0.001673	1	0.96737442	1	0.999996
Thorax length of eclosed males	Treatment:Isofemale_line	4.333453	6	0.631648636	1	0.983629
Development time of eclosed females	Treatment	0.010573	1	0.918101234	1	0.999996
Development time of eclosed females	Location	0.000157	1	0.989999079	1	0.999996
Development time of eclosed females	Isofemale_line	4.726732	11	0.943675097	1	0.999996
Development time of eclosed females	Treatment:Location	9.241336	1	0.002366122	0.106475	0.00968
**Development time of eclosed females**	**Treatment:Isofemale_line**	**51.49119**	**11**	**3.37E-07**	1.52E-05	5.06E-06
Thorax length of eclosed females	Treatment	0.078477	1	0.779371917	1	0.999996
Thorax length of eclosed females	Location	0.00391	1	0.95014304	1	0.999996

Table S8 (part 2)

Thorax length of eclosed females	Isofemale_line	8.187468	11	0.696418776	1	0.999996
Thorax length of eclosed females	Treatment:Location	4.121233	1	0.042348208	1	0.119104
**Thorax length of eclosed females**	**Treatment:Isofemale_line**	**30.55975**	**11**	**0.001293425**	0.058204	0.006474
Longevity	Treatment	0.275705	1	0.599530446	1	0.983629
Longevity	Location	0.023533	1	0.878079426	1	0.999996
Longevity	Isofemale_line	3.689288	6	0.718638882	1	0.999996
Longevity	Treatment:Location	0.651298	1	0.419649008	1	0.821052
**Longevity**	**Treatment:Isofemale_line**	**20.45317**	**6**	**0.002299045**	0.103457	0.00968
Fecundity	Treatment	5.268072	1	0.021720061	0.977403	0.069814
Fecundity	Location	0.328921	1	0.566294925	1	0.983629
Fecundity	Isofemale_line	3.406954	5	0.637510965	1	0.983629
Fecundity	Treatment:Location	2.865173	1	0.090515976	1	0.239601
Fecundity	Treatment:Isofemale_line	3.741355	5	0.587221859	1	0.983629

Table S9

	Location	Isofemale_	contrast	estimate	SE	df	asymp.LCL	asymp.UCL	z.ratio	p.value
1	E	F_E1#32	Control - Mycotoxin	0.011894	0.517899	Inf	-1.823434786	1.84722213	0.022965	1
2	E	F_E10#18	Control - Mycotoxin	-0.43473	0.466979	Inf	-2.089608159	1.22015108	-0.93094	0.999995
3	E	F_E10#19	Control - Mycotoxin	-0.5935	0.547343	Inf	-2.533173756	1.34617693	-1.08433	0.999945
4	E	F_E14#14	Control - Mycotoxin	-1.33785	0.45337	Inf	-2.944504625	0.26880198	-2.9509	0.258885
5	E	F_E14#28	Control - Mycotoxin	0.859355	0.546316	Inf	-1.076680863	2.79539068	1.572998	0.99136
6	E	F_E14#3	Control - Mycotoxin	-1.01976	0.461518	Inf	-2.655286316	0.61577102	-2.20957	0.794965
7	E	F_E19#102	Control - Mycotoxin	1.270061	0.500827	Inf	-0.504771363	3.04489239	2.535924	0.555477
8	E	F_E20#110	Control - Mycotoxin	1.709892	0.478298	Inf	0.014898497	3.40488482	3.574948	0.045132
9	E	F_E4#7	Control - Mycotoxin	0.70119	0.536339	Inf	-1.199487662	2.60186723	1.307363	0.999178
10	E	F_E9#8	Control - Mycotoxin	0.118947	0.48534	Inf	-1.601000926	1.83889399	0.245079	1
11	G	F_G1#1	Control - Mycotoxin	0.332772	0.470605	Inf	-1.33495629	2.00050009	0.707116	1
12	G	F_G1#16	Control - Mycotoxin	0.433398	0.466507	Inf	-1.219810203	2.0866058	0.929027	0.999995
13	G	F_G1#17	Control - Mycotoxin	0.273539	0.522448	Inf	-1.577910545	2.12498867	0.523572	1
14	G	F_G1#18	Control - Mycotoxin	0.662234	0.51953	Inf	-1.178876791	2.50334492	1.274679	0.99942
15	G	F_G1#44	Control - Mycotoxin	1.002936	0.485247	Inf	-0.716681435	2.72255309	2.066857	0.873824
16	G	F_G1#47	Control - Mycotoxin	0.435899	0.538769	Inf	-1.473388842	2.3451863	0.809065	1
17	G	F_G1#5	Control - Mycotoxin	-1.78974	0.493323	Inf	-3.537976295	-0.04150058	-3.62792	0.037789
18	G	F_G1#61	Control - Mycotoxin	0.398391	0.629968	Inf	-1.83408868	2.63087023	0.632399	1
19	G	F_G1#64	Control - Mycotoxin	0.525946	0.46146	Inf	-1.109375976	2.1612685	1.139744	0.999884
20	G	F_G1#9	Control - Mycotoxin	-0.18869	0.436707	Inf	-1.736288403	1.35891447	-0.43207	1

Table S10

	Location	Isofemale_	contrast	estimate	SE	df	asymp.LCL	asymp.UCL	z.ratio	p.value
1	E	F_E1#32	Control - Mycotoxin	0.662187	0.674016	Inf	-1.7263887	3.0507627	0.982451	0.999988
2	E	F_E10#18	Control - Mycotoxin	-0.16641	0.571077	Inf	-2.1901876	1.8573764	-0.29139	1
3	E	F_E10#19	Control - Mycotoxin	-0.99357	0.863001	Inf	-4.0518729	2.0647348	-1.15129	0.999865
4	E	F_E14#14	Control - Mycotoxin	-0.8782	0.557095	Inf	-2.8524329	1.0960312	-1.57639	0.99114
5	E	F_E14#28	Control - Mycotoxin	0.311823	0.793981	Inf	-2.5018853	3.1255307	0.392733	1
6	E	F_E14#3	Control - Mycotoxin	-1.02334	0.524077	Inf	-2.8805658	0.833882	-1.95266	0.921045
7	E	F_E19#102	Control - Mycotoxin	0.649784	0.516437	Inf	-1.180364	2.4799316	1.258206	0.999516
8	E	F_E20#110	Control - Mycotoxin	1.855576	0.533585	Inf	-0.0353432	3.7464947	3.477562	0.061858
9	E	F_E4#7	Control - Mycotoxin	0.95577	0.725231	Inf	-1.6143014	3.5258418	1.317885	0.999083
10	E	F_E9#8	Control - Mycotoxin	0.458966	0.683209	Inf	-1.9621898	2.8801221	0.67178	1
11	G	F_G1#1	Control - Mycotoxin	0.556025	0.615601	Inf	-1.6255425	2.7375927	0.903223	0.999997
12	G	F_G1#16	Control - Mycotoxin	0.148668	0.541918	Inf	-1.7717807	2.0691176	0.274337	1
13	G	F_G1#17	Control - Mycotoxin	0.952425	0.643664	Inf	-1.3285911	3.2334403	1.479692	0.995861
14	G	F_G1#18	Control - Mycotoxin	0.315165	0.560884	Inf	-1.6724937	2.3028234	0.561908	1
15	G	F_G1#44	Control - Mycotoxin	0.854521	0.505121	Inf	-0.9355275	2.6445699	1.691715	0.980466
16	G	F_G1#47	Control - Mycotoxin	0.186635	0.610558	Inf	-1.977059	2.3503282	0.305679	1
17	G	F_G1#5	Control - Mycotoxin	-1.91747	0.569647	Inf	-3.936186	0.1012398	-3.36608	0.087123
18	G	F_G1#61	Control - Mycotoxin	1.71551	1.113912	Inf	-2.2319686	5.6629894	1.540078	0.993268
19	G	F_G1#64	Control - Mycotoxin	0.53173	0.520038	Inf	-1.3111787	2.3746387	1.022484	0.999978
20	G	F_G1#9	Control - Mycotoxin	0.194	0.445745	Inf	-1.3856292	1.7736295	0.435227	1

Table S11

	Location	Isofemale_	contrast	estimate	SE	df	lower.CL	upper.CL	t.ratio	p.value
1	E	F_E1#32	Control - Mycotoxin	0.944397	0.407168	512.9903	-0.50619	2.394989	2.319428	0.720147
2	E	F_E10#18	Control - Mycotoxin	-1.17317	0.406685	502.7215	-2.62219	0.275859	-2.88471	0.303164
3	E	F_E10#19	Control - Mycotoxin	1.709335	0.374662	485.0232	0.374143	3.044528	4.562335	0.00109
4	E	F_E14#14	Control - Mycotoxin	0.789179	0.251322	477.3258	-0.10654	1.6849	3.140115	0.168522
5	E	F_E14#28	Control - Mycotoxin	-2.10072	0.485727	486.7381	-3.83168	-0.36976	-4.3249	0.00301
6	E	F_E14#3	Control - Mycotoxin	0.094845	0.244641	476.5308	-0.77708	0.966765	0.38769	1
7	E	F_E19#102	Control - Mycotoxin	-0.00016	0.353319	502.4137	-1.25905	1.258725	-0.00046	1
8	E	F_E20#110	Control - Mycotoxin	-1.45052	0.297514	475.8409	-2.51089	-0.39014	-4.87546	0.000262
9	E	F_E4#7	Control - Mycotoxin	-0.71868	0.428503	477.6868	-2.24587	0.808517	-1.67719	0.98143
10	E	F_E9#8	Control - Mycotoxin	0.130556	0.372613	511.3068	-1.19695	1.458061	0.350379	1
11	G	F_G1#1	Control - Mycotoxin	-1.33695	0.339462	509.1289	-2.54638	-0.12752	-3.93844	0.013668
12	G	F_G1#16	Control - Mycotoxin	-0.14982	0.306818	476.1983	-1.24335	0.943708	-0.4883	1
13	G	F_G1#17	Control - Mycotoxin	-0.86884	0.419943	512.0992	-2.36496	0.627277	-2.06895	0.871032
14	G	F_G1#18	Control - Mycotoxin	0.392177	0.340437	499.2156	-0.82085	1.605206	1.151982	0.999852
15	G	F_G1#44	Control - Mycotoxin	-0.24632	0.331752	479.1205	-1.42867	0.936039	-0.74247	1
16	G	F_G1#47	Control - Mycotoxin	-0.11911	0.361864	496.3696	-1.40853	1.170306	-0.32917	1
17	G	F_G1#5	Control - Mycotoxin	-0.49306	0.33969	498.8277	-1.70343	0.717316	-1.45149	0.996544
18	G	F_G1#61	Control - Mycotoxin	-0.90983	0.530036	512.0159	-2.79817	0.978515	-1.71654	0.976337
19	G	F_G1#64	Control - Mycotoxin	-0.99603	0.331528	510.1867	-2.17718	0.185116	-3.00437	0.233387
20	G	F_G1#9	Control - Mycotoxin	-0.8297	0.241971	475.4682	-1.69211	0.032718	-3.42891	0.075925

Table S12

	Location	Isofemale_line	contrast	estimate	SE	df	lower.CL	upper.CL	t.ratio	p.value
1	E	F_E10#18	Control - N	-1.53468	1.030272	60.26285	-4.98118	1.911814	-1.48959	0.917638
2	E	F_E14#14	Control - N	2.457183	0.973963	44.72779	-0.84464	5.75901	2.522872	0.319308
3	E	F_E14#3	Control - N	0.523992	0.701825	46.64792	-1.85021	2.898197	0.746613	0.999542
4	E	F_E20#110	Control - N	-1.65634	0.598492	44.66196	-3.68544	0.372764	-2.76751	0.204042
5	E	F_E4#7	Control - N	-0.40465	1.122584	59.67535	-4.16137	3.352071	-0.36046	1
6	G	F_G1#16	Control - N	-0.5	1.880577	16.73627	-7.44555	6.445546	-0.26588	1
7	G	F_G1#18	Control - N	-1.82256	1.233871	60.65765	-5.94911	2.303991	-1.47711	0.921709
8	G	F_G1#44	Control - N	0.094105	0.785452	49.31579	-2.55589	2.744102	0.11981	1
9	G	F_G1#47	Control - N	-0.2102	0.932744	50.75318	-3.35296	2.932564	-0.22536	1
10	G	F_G1#64	Control - N	-0.47122	1.227444	59.78679	-4.57856	3.636114	-0.38391	0.999999
11	G	F_G1#9	Control - N	1.924471	1.169867	60.89007	-1.98745	5.836396	1.645034	0.856259

Table S13

	Location	Isofemale_	contrast	estimate	SE	df	lower.CL	upper.CL	t.ratio	p.value
1	E	F_E10#18	Control - Mycotoxin	38.47541	42.01151	60.96695	-102	178.9512	0.91583	0.997547
2	E	F_E14#14	Control - Mycotoxin	-41.6051	42.01313	46.01631	-183.828	100.6178	-0.99029	0.99512
3	E	F_E14#3	Control - Mycotoxin	-104.692	30.10924	48.31149	-206.375	-3.00893	-3.47707	0.0386
4	E	F_E20#110	Control - Mycotoxin	7.51974	25.83874	46.14472	-79.9373	94.97681	0.291026	1
5	E	F_E4#7	Control - Mycotoxin	113.8834	45.99707	60.44306	-39.9697	267.7365	2.475885	0.33887
6	G	F_G1#16	Control - Mycotoxin	-52.705	65.03607	17.87596	-290.828	185.4185	-0.8104	0.998644
7	G	F_G1#18	Control - Mycotoxin	-80.7116	49.29211	58.79993	-245.763	84.33951	-1.63741	0.85953
8	G	F_G1#44	Control - Mycotoxin	-31.3845	33.62472	54.08884	-144.361	81.59195	-0.93338	0.997067
9	G	F_G1#47	Control - Mycotoxin	-3.56848	39.5689	53.71537	-136.556	129.4195	-0.09018	1
10	G	F_G1#64	Control - Mycotoxin	-19.8357	48.68762	55.50942	-183.243	143.572	-0.40741	0.999998
11	G	F_G1#9	Control - Mycotoxin	-72.3426	47.5173	57.6017	-231.581	86.89598	-1.52245	0.906104

Table S14

	Location	Isofemale_	contrast	estimate	SE	df	lower.CL	upper.CL	t.ratio	p.value
1	E	F_E10#19	Control - Mycotoxin	-0.2136	1.331123	112.9387	-4.71933	4.292133	-0.16046	1
2	E	F_E14#14	Control - Mycotoxin	2.04249	0.843008	114.9086	-0.80996	4.894945	2.42286	0.435939
3	E	F_E14#3	Control - Mycotoxin	-1.37279	0.982419	116.3478	-4.69609	1.95052	-1.39735	0.972721
4	E	F_E19#102	Control - Mycotoxin	-0.32384	0.75178	116.9958	-2.86665	2.218975	-0.43076	1
5	G	F_G1#1	Control - Mycotoxin	-0.9406	1.099351	81.38596	-4.69287	2.811667	-0.8556	0.999677
6	G	F_G1#16	Control - Mycotoxin	-0.24074	0.851141	111.2602	-3.12271	2.641228	-0.28285	1
7	G	F_G1#17	Control - Mycotoxin	-0.49974	0.88404	106.9628	-3.49572	2.496251	-0.56529	0.999997
8	G	F_G1#18	Control - Mycotoxin	0.374916	0.812797	100.051	-2.38395	3.133777	0.461266	1
9	G	F_G1#44	Control - Mycotoxin	0.252349	0.978895	111.9381	-3.06176	3.566455	0.25779	1
10	G	F_G1#47	Control - Mycotoxin	-0.13838	1.020032	112.8793	-3.59114	3.314371	-0.13567	1
11	G	F_G1#5	Control - Mycotoxin	1.034661	0.808998	109.2145	-1.70573	3.775049	1.278941	0.986615
12	G	F_G1#64	Control - Mycotoxin	-0.81497	0.855059	96.42977	-3.71992	2.089977	-0.95312	0.99908
13	G	F_G1#9	Control - Mycotoxin	1.334158	0.581591	102.601	-0.63874	3.307052	2.293981	0.526631

Table S15

	Location	Isofemale_	contrast	estimate	SE	df	lower.CL	upper.CL	t.ratio	p.value
1	E	F_E10#19	Control - Mycotoxin	60.51	61.45006	106.9698	-147.742	268.7622	0.984702	0.998756
2	E	F_E14#14	Control - Mycotoxin	-26.4261	41.33471	102.6406	-166.642	113.7898	-0.63932	0.999986
3	E	F_E14#3	Control - Mycotoxin	152.95	47.64779	115.5939	-8.25386	314.1539	3.210012	0.080782
4	E	F_E19#102	Control - Mycotoxin	-34.5787	35.52572	90.06917	-155.487	86.32946	-0.97334	0.998856
5	G	F_G1#1	Control - Mycotoxin	6.967	45.83464	53.07632	-151.968	165.9016	0.152003	1
6	G	F_G1#16	Control - Mycotoxin	-5.84	42.69736	116.9876	-150.259	138.5792	-0.13678	1
7	G	F_G1#17	Control - Mycotoxin	-25.3544	44.96106	116.2907	-177.449	126.7404	-0.56392	0.999997
8	G	F_G1#18	Control - Mycotoxin	-25.624	42.36964	108.4063	-169.17	117.9221	-0.60477	0.999993
9	G	F_G1#44	Control - Mycotoxin	-12.756	48.96812	115.8254	-178.42	152.9079	-0.2605	1
10	G	F_G1#47	Control - Mycotoxin	42.46	51.05003	114.1209	-130.301	215.2212	0.831733	0.999774
11	G	F_G1#5	Control - Mycotoxin	-69.9861	40.91159	116.3863	-208.38	68.40781	-1.71067	0.886375
12	G	F_G1#64	Control - Mycotoxin	-38.165	45.08503	100.018	-191.197	114.8674	-0.84651	0.999723
13	G	F_G1#9	Control - Mycotoxin	-17.8444	30.48809	116.1922	-120.982	85.29291	-0.58529	0.999995

Table S16

	Location	Isofemale_	contrast	estimate	SE	df	lower.CL	upper.CL	t.ratio	p.value
1	E	F_E10#18	Control - Mycotoxin	31.84515	19.14249	60.99031	-32.1616	95.85185	1.663584	0.847569
2	E	F_E14#14	Control - Mycotoxin	-24.3884	20.59135	48.43126	-93.92	45.14317	-1.1844	0.981333
3	E	F_E14#3	Control - Mycotoxin	-22.9708	14.6409	50.66452	-72.3054	26.36379	-1.56895	0.887693
4	E	F_E20#110	Control - Mycotoxin	-20.4558	12.70526	49.81025	-63.3015	22.38988	-1.61003	0.870529
5	E	F_E4#7	Control - Mycotoxin	50.25356	21.20346	60.26959	-20.6766	121.1837	2.370064	0.402505
6	G	F_G1#16	Control - Mycotoxin	27.5	25.04318	22.45136	-61.8378	116.8378	1.098103	0.987052
7	G	F_G1#18	Control - Mycotoxin	-47.2388	21.84197	58.11589	-120.409	25.93135	-2.16275	0.539336
8	G	F_G1#44	Control - Mycotoxin	4.84216	16.17579	60.80303	-49.2511	58.93543	0.299346	1
9	G	F_G1#47	Control - Mycotoxin	24.02716	18.84551	56.57031	-39.1736	87.22793	1.274954	0.969396
10	G	F_G1#64	Control - Mycotoxin	5.52759	21.20157	51.5486	-65.8582	76.91342	0.260716	1
11	G	F_G1#9	Control - Mycotoxin	8.161523	20.74514	44.21113	-62.2089	78.53198	0.393419	0.999999

Table S17

	Location	Isofemale_line	contrast	estimate	SE	df	lower.CL	upper.CL	t.ratio	p.value
1	E	F_E14#14	Control - Mycotoxin	61.62463	56.86147	45.11461	-123.558	246.8072	1.083768	0.973722
2	E	F_E14#3	Control - Mycotoxin	-101.8	85.7578	53.99988	-378.862	175.2618	-1.18707	0.955884
3	E	F_E19#102	Control - Mycotoxin	-18.9687	54.02756	50.88602	-193.953	156.0158	-0.35109	0.999992
4	G	F_G1#1	Control - Mycotoxin	39.40022	92.20077	13.55049	-296.798	375.5985	0.427331	0.999942
5	G	F_G1#18	Control - Mycotoxin	37.49993	73.82139	47.02561	-202.441	277.4414	0.507982	0.999863
6	G	F_G1#44	Control - Mycotoxin	-40.8327	81.5165	53.94468	-304.203	222.5376	-0.50091	0.99988
7	G	F_G1#5	Control - Mycotoxin	-108.684	73.54563	47.18117	-347.693	130.324	-1.47778	0.860005
8	G	F_G1#64	Control - Mycotoxin	33.1231	53.0939	40.53828	-140.742	206.9881	0.623859	0.999355
9	G	F_G1#9	Control - Mycotoxin	-53.0564	44.67119	47.90972	-198.126	92.01316	-1.18771	0.955309

Table S18

	Location	Isofemale_line	contrast	estimate	SE	df	asymp.LCL	asymp.UCL	z.ratio	p.value
1	E	R_E1#49	Control - lV	0.2874	0.437645	Inf	-1.2635269	1.83832703	0.656696	1
2	E	R_E10#56	Control - lV	0.949992	0.498161	Inf	-0.8153902	2.71537463	1.906998	0.935984
3	E	R_E15#11	Control - lV	1.958009	0.49307	Inf	0.2106689	3.70534826	3.971059	0.010839
4	E	R_E24#41	Control - lV	1.063052	0.450007	Inf	-0.5316837	2.657787	2.362299	0.689866
5	E	R_E3#51	Control - lV	0.671303	0.442072	Inf	-0.8953119	2.23791741	1.518537	0.994316
6	E	R_E31#5	Control - lV	-0.67454	0.523361	Inf	-2.5292267	1.18014849	-1.28886	0.999324
7	E	R_E5#44	Control - lV	-2.01E-05	0.330379	Inf	-1.1708158	1.17077564	-6.08E-05	1
8	E	R_E5#53	Control - lV	-0.51419	0.455304	Inf	-2.1276963	1.09931286	-1.12934	0.999899
9	E	R_E6#52	Control - lV	-1.56835	0.605444	Inf	-3.7139188	0.57722292	-2.59041	0.512492
10	E	R_E8#57	Control - lV	0.225126	0.474504	Inf	-1.456421	1.9066723	0.474444	1
11	G	R_G1#11	Control - lV	-1.21769	0.836227	Inf	-4.1811127	1.74572653	-1.45618	0.996611
12	G	R_G1#14	Control - lV	0.18814	0.432592	Inf	-1.3448777	1.72115757	0.434914	1
13	G	R_G1#17	Control - lV	0.758246	0.509755	Inf	-1.0482214	2.56471424	1.487473	0.995584
14	G	R_G1#20	Control - lV	0.554907	0.473692	Inf	-1.1237644	2.23357766	1.171449	0.999826
15	G	R_G1#26	Control - lV	2.09711	0.821231	Inf	-0.8131672	5.00738636	2.553618	0.541495
16	G	R_G1#3	Control - lV	0.871075	0.508128	Inf	-0.9296274	2.67177778	1.714284	0.977504
17	G	R_G1#31	Control - lV	-0.09445	0.433782	Inf	-1.6316851	1.44278925	-0.21773	1
18	G	R_G1#35	Control - lV	0.303388	0.450414	Inf	-1.29279	1.89956606	0.673575	1
19	G	R_G1#5	Control - lV	1.206374	0.463386	Inf	-0.4357729	2.84852003	2.603389	0.502308
20	G	R_G1#8	Control - lV	2.221263	0.698399	Inf	-0.2537231	4.69624993	3.180507	0.147186

Table S19

	Location	Isofemale_line	contrast	estimate	SE	df	asymp.LCL	asymp.UCL	z.ratio	p.value
1	E	R_E1#49	Control - IV	0.003038	0.495256	Inf	-1.7520499	1.75812544	0.006134	1
2	E	R_E10#56	Control - IV	1.249558	0.635914	Inf	-1.0039938	3.50310954	1.964979	0.916635
3	E	R_E15#11	Control - IV	2.11118	0.799373	Inf	-0.7216375	4.94399766	2.641045	0.472972
4	E	R_E24#41	Control - IV	1.543665	0.546155	Inf	-0.3917982	3.47912863	2.826424	0.337378
5	E	R_E3#51	Control - IV	1.408167	0.695719	Inf	-1.0573213	3.87365505	2.024046	0.893208
6	E	R_E31#5	Control - IV	-0.40937	0.522735	Inf	-2.2618328	1.44310071	-0.78312	1
7	E	R_E5#44	Control - IV	0.121055	0.345588	Inf	-1.1036396	1.34575025	0.350288	1
8	E	R_E5#53	Control - IV	0.134956	0.521331	Inf	-1.7125372	1.98244897	0.258868	1
9	E	R_E6#52	Control - IV	-0.73978	0.61927	Inf	-2.9343428	1.45479065	-1.19459	0.999769
10	E	R_E8#57	Control - IV	-0.00087	0.561233	Inf	-1.9897695	1.98802329	-0.00156	1
11	G	R_G1#11	Control - IV	-1.21238	0.841104	Inf	-4.1930814	1.76832925	-1.44141	0.99702
12	G	R_G1#14	Control - IV	0.992214	0.551644	Inf	-0.9627014	2.94712943	1.798649	0.96322
13	G	R_G1#17	Control - IV	0.460409	0.680434	Inf	-1.9509138	2.87173131	0.67664	1
14	G	R_G1#20	Control - IV	0.961209	0.722671	Inf	-1.5997909	3.52220958	1.330079	0.998961
15	G	R_G1#26	Control - IV	2.765535	1.075586	Inf	-1.0461257	6.57719568	2.571189	0.527626
16	G	R_G1#3	Control - IV	0.586083	0.544916	Inf	-1.3449894	2.51715498	1.075547	0.999952
17	G	R_G1#31	Control - IV	-0.69708	0.535353	Inf	-2.594268	1.20010225	-1.3021	0.999222
18	G	R_G1#35	Control - IV	1.345785	0.62489	Inf	-0.8686997	3.56026956	2.153635	0.828396
19	G	R_G1#5	Control - IV	0.999014	0.552629	Inf	-0.9593919	2.95742018	1.807748	0.961344
20	G	R_G1#8	Control - IV	3.099707	1.07211	Inf	-0.6996369	6.89905162	2.89122	0.295033

Table S20

	Location	Isofemale_	contrast	estimate	SE	df	lower.CL	upper.CL	t.ratio	p.value
1	E	R_E1#49	Control - Mycotoxin	0.21859	0.317085	585.1193	-0.91033	1.347511	0.689372	1
2	E	R_E10#56	Control - Mycotoxin	0.655144	0.420327	608.747	-0.84108	2.15137	1.558652	0.99191
3	E	R_E15#11	Control - Mycotoxin	0.137536	0.312778	588.4396	-0.97602	1.251092	0.439725	1
4	E	R_E24#41	Control - Mycotoxin	-1.51765	0.319759	585.1233	-2.65609	-0.37921	-4.74622	0.000456
5	E	R_E3#51	Control - Mycotoxin	0.788856	0.337338	583.6384	-0.41219	1.989899	2.338472	0.706642
6	E	R_E31#5	Control - Mycotoxin	1.392026	0.422915	614.9551	-0.11334	2.897396	3.291505	0.111806
7	E	R_E5#44	Control - Mycotoxin	1.434181	0.229617	600.9449	0.616773	2.25159	6.245968	1.51E-07
8	E	R_E5#53	Control - Mycotoxin	-0.62653	0.367384	584.4189	-1.93454	0.681478	-1.70538	0.977998
9	E	R_E6#52	Control - Mycotoxin	-1.33963	0.553725	605.7168	-3.31076	0.631492	-2.41931	0.64664
10	E	R_E8#57	Control - Mycotoxin	0.049745	0.383678	586.1779	-1.31626	1.415745	0.129652	1
11	G	R_G1#11	Control - Mycotoxin	0.439766	0.944791	613.4052	-2.92326	3.802796	0.465463	1
12	G	R_G1#14	Control - Mycotoxin	-0.19605	0.318001	582.9861	-1.32825	0.936153	-0.61651	1
13	G	R_G1#17	Control - Mycotoxin	0.361195	0.483245	612.0363	-1.35896	2.081346	0.747437	1
14	G	R_G1#20	Control - Mycotoxin	0.416637	0.393403	583.113	-0.98402	1.817298	1.059059	0.999958
15	G	R_G1#26	Control - Mycotoxin	-0.4036	0.788288	595.9565	-3.20992	2.402712	-0.512	1
16	G	R_G1#3	Control - Mycotoxin	-1.22372	0.470678	618.7119	-2.89905	0.451622	-2.5999	0.506373
17	G	R_G1#31	Control - Mycotoxin	0.182007	0.29753	586.1192	-0.87728	1.241296	0.611726	1
18	G	R_G1#35	Control - Mycotoxin	-0.37512	0.356646	587.603	-1.64486	0.894626	-1.0518	0.999963
19	G	R_G1#5	Control - Mycotoxin	0.129377	0.363925	588.3136	-1.16628	1.425032	0.355505	1
20	G	R_G1#8	Control - Mycotoxin	-0.72688	0.636799	589.4951	-2.994	1.540249	-1.14145	0.999872

Table S21

	Location	Isofemale_	contrast	estimate	SE	df	lower.CL	upper.CL	t.ratio	p.value
1	E	R_E1#49	Control - Mycotoxin	-0.79521	1.31028	73.53911	-5.44311	3.852699	-0.6069	1
2	E	R_E10#56	Control - Mycotoxin	-0.96657	1.082851	73.69296	-4.80745	2.874312	-0.89262	0.99993
3	E	R_E15#11	Control - Mycotoxin	0.350603	1.034882	70.99865	-3.32493	4.026135	0.338786	1
4	E	R_E24#41	Control - Mycotoxin	-1.15094	0.904742	75.1428	-4.35794	2.056058	-1.27212	0.995816
5	E	R_E3#51	Control - Mycotoxin	1	1.154723	80.21669	-3.08435	5.084355	0.866008	0.999954
6	E	R_E31#5	Control - Mycotoxin	-0.16423	0.979561	89.07818	-3.61811	3.28966	-0.16765	1
7	E	R_E5#44	Control - Mycotoxin	3.727152	0.922745	88.89248	0.473402	6.980903	4.039201	0.010241
8	E	R_E5#53	Control - Mycotoxin	-0.0731	0.885543	69.63369	-3.22043	3.074236	-0.08254	1
9	E	R_E6#52	Control - Mycotoxin	0	1.209868	65.87903	-4.30895	4.30895	0	1
10	G	R_G1#11	Control - Mycotoxin	3	2.346051	46.04613	-5.49442	11.49442	1.278745	0.994878
11	G	R_G1#17	Control - Mycotoxin	-1.23292	1.232817	92.81302	-5.57479	3.108942	-1.00009	0.999739
12	G	R_G1#26	Control - Mycotoxin	-2.70759	1.339564	69.79153	-7.46818	2.052997	-2.02125	0.805135
13	G	R_G1#3	Control - Mycotoxin	-0.00518	1.153035	82.24554	-4.08038	4.07002	-0.00449	1
14	G	R_G1#35	Control - Mycotoxin	-1.5	1.306353	92.99551	-6.1006	3.100604	-1.14823	0.998693
15	G	R_G1#5	Control - Mycotoxin	0.016598	1.172231	92.77087	-4.11194	4.145135	0.014159	1
16	G	R_G1#8	Control - Mycotoxin	NA	NA	NA	NA	NA	NA	NA

Table S22

	Location	Isofemale_	contrast	estimate	SE	df	lower.CL	upper.CL	t.ratio	p.value
1	E	R_E1#49	Control - Mycotoxin	109.51	51.8578	50.39129	-77.3706	296.3905	2.111735	0.749097
2	E	R_E10#56	Control - Mycotoxin	62.69299	47.77193	79.22306	-106.348	231.7335	1.31234	0.994304
3	E	R_E15#11	Control - Mycotoxin	40.71	45.84308	75.203	-121.783	203.2034	0.888029	0.999935
4	E	R_E24#41	Control - Mycotoxin	29.46194	39.90463	82.76119	-111.547	170.4707	0.738309	0.999994
5	E	R_E3#51	Control - Mycotoxin	-16.6489	50.31694	85.87849	-194.253	160.9551	-0.33088	1
6	E	R_E31#5	Control - Mycotoxin	25.11698	42.02991	92.40242	-122.926	173.1603	0.597598	1
7	E	R_E5#44	Control - Mycotoxin	15.42428	37.88901	62.16502	-119.828	150.6762	0.407091	1
8	E	R_E5#53	Control - Mycotoxin	24.67083	39.30189	72.97396	-114.781	164.1222	0.627726	0.999999
9	E	R_E6#52	Control - Mycotoxin	2.865	53.96207	66.63015	-189.238	194.9679	0.053093	1
10	G	R_G1#11	Control - Mycotoxin	-2.19	90.83474	56.38519	-327.796	323.4156	-0.02411	1
11	G	R_G1#17	Control - Mycotoxin	74.2025	51.35287	89.26499	-106.855	255.2596	1.444953	0.985672
12	G	R_G1#26	Control - Mycotoxin	-10.0184	59.43503	73.13018	-220.891	200.8539	-0.16856	1
13	G	R_G1#3	Control - Mycotoxin	-5.36571	50.12811	89.4345	-182.095	171.3636	-0.10704	1
14	G	R_G1#35	Control - Mycotoxin	36.77743	54.69552	86.76653	-156.223	229.7781	0.672403	0.999998
15	G	R_G1#5	Control - Mycotoxin	26.47078	48.82206	87.78937	-145.746	198.6873	0.542189	1
16	G	R_G1#8	Control - Mycotoxin	NA	NA	NA	NA	NA	NA	NA

Table S23

	Location	Isofemale_	contrast	estimate	SE	df	lower.CL	upper.CL	t.ratio	p.value
1	E	R_E1#49	Control - Mycotoxin	-1.15932	0.986821	94.03516	-4.41517	2.096531	-1.1748	0.983754
2	E	R_E24#41	Control - Mycotoxin	-0.66342	1.283081	99.98927	-4.89051	3.563669	-0.51705	0.999986
3	E	R_E31#5	Control - Mycotoxin	-0.41286	0.786876	85.2341	-3.01565	2.189926	-0.52469	0.999984
4	E	R_E5#44	Control - Mycotoxin	2.360068	0.669646	98.76001	0.153293	4.566843	3.524352	0.025701
5	E	R_E5#53	Control - Mycotoxin	-0.94083	0.919472	97.67375	-3.97169	2.090021	-1.02323	0.994375
6	E	R_E6#52	Control - Mycotoxin	-1.33971	1.025082	92.81859	-4.72289	2.043466	-1.30693	0.965392
7	E	R_E8#57	Control - Mycotoxin	0.571318	0.869426	80.18591	-2.30946	3.452096	0.657121	0.99987
8	G	R_G1#20	Control - Mycotoxin	0.442025	1.295037	96.85318	-3.82766	4.711713	0.341322	1
9	G	R_G1#3	Control - Mycotoxin	-0.61811	1.001774	99.80283	-3.91858	2.682362	-0.61701	0.99993
10	G	R_G1#31	Control - Mycotoxin	-0.26275	1.009772	86.0281	-3.60198	3.076488	-0.2602	1
11	G	R_G1#5	Control - Mycotoxin	1.478851	1.177244	99.82877	-2.3997	5.357405	1.256197	0.973827

Table S24

	Location	Isofemale_	contrast	estimate	SE	df	lower.CL	upper.CL	t.ratio	p.value
1	E	R_E1#49	Control - Mycotoxin	83.02073	43.66297	98.63745	-60.872	226.9134	1.901399	0.714875
2	E	R_E24#41	Control - Mycotoxin	61.13708	52.94567	72.68794	-114.818	237.0925	1.154714	0.985311
3	E	R_E31#5	Control - Mycotoxin	-16.5586	35.87816	89.28631	-135.088	101.9708	-0.46152	0.999995
4	E	R_E5#44	Control - Mycotoxin	-5.91197	28.88282	69.11218	-102.057	90.23339	-0.20469	1
5	E	R_E5#53	Control - Mycotoxin	-28.8485	39.60332	99.51481	-159.335	101.6384	-0.72844	0.999684
6	E	R_E6#52	Control - Mycotoxin	105.7168	45.25627	99.99438	-43.379	254.8126	2.335959	0.41837
7	E	R_E8#57	Control - Mycotoxin	28.86752	40.31654	83.63058	-104.559	162.2942	0.716022	0.999721
8	G	R_G1#20	Control - Mycotoxin	-67.5127	56.23072	99.58373	-252.781	117.756	-1.20064	0.981051
9	G	R_G1#3	Control - Mycotoxin	46.13923	41.37887	56.31119	-92.6561	184.9346	1.115043	0.988277
10	G	R_G1#31	Control - Mycotoxin	-38.6824	45.85221	93.77728	-189.974	112.6095	-0.84363	0.998851
11	G	R_G1#5	Control - Mycotoxin	106.7733	48.06213	55.53814	-54.5316	268.0782	2.221568	0.500049

Table S25

	Location	Isofemale_line	contrast	estimate	SE	df	lower.CL	upper.CL	t.ratio	p.value
1	E	R_E1#49	Control - Mycotoxin	2.5	22.25622	19.28578	-80.9819	85.98188	0.112328	1
2	E	R_E10#56	Control - Mycotoxin	-63	25.93693	72.7489	-151.84	25.83985	-2.42897	0.43639
3	E	R_E15#11	Control - Mycotoxin	56	25.64488	66.58681	-32.1098	144.1098	2.183671	0.606029
4	E	R_E24#41	Control - Mycotoxin	-30.5	25.40935	73.98243	-117.485	56.48458	-1.20035	0.991791
5	E	R_E3#51	Control - Mycotoxin	-15.3333	27.94618	68.49298	-111.253	80.58634	-0.54867	0.999997
6	E	R_E31#5	Control - Mycotoxin	-23	22.80183	47.08269	-102.524	56.52412	-1.00869	0.99811
7	E	R_E5#44	Control - Mycotoxin	-15.625	17.08798	9.728	-87.798	56.54802	-0.91439	0.997989
8	E	R_E5#53	Control - Mycotoxin	-23.5	21.68484	66.29575	-98.0159	51.01587	-1.08371	0.996635
9	E	R_E6#52	Control - Mycotoxin	27.5	30.09889	54.69742	-76.7297	131.7297	0.913655	0.999306
10	G	R_G1#17	Control - Mycotoxin	34.33333	25.24753	62.96797	-52.5921	121.2587	1.359869	0.976395
11	G	R_G1#3	Control - Mycotoxin	-9.9	26.22955	70.70003	-99.8286	80.02864	-0.37744	1
12	G	R_G1#35	Control - Mycotoxin	11.1	26.30601	45.3563	-80.8237	103.0237	0.421957	1
13	G	R_G1#5	Control - Mycotoxin	-2	23.84954	55.01988	-84.5674	80.56745	-0.08386	1

Table S26

	Location	Isofemale_	contrast	estimate	SE	df	lower.CL	upper.CL	t.ratio	p.value
1	E	R_E1#49	Control - Mycotoxin	-34	52.13938	21.38518	-217.297	149.2971	-0.6521	0.999553
2	E	R_E24#41	Control - Mycotoxin	26.64286	49.52044	40.36421	-139.058	192.3435	0.538017	0.999927
3	E	R_E3#51	Control - Mycotoxin	-49.6667	67.66848	57.0098	-272.427	173.094	-0.73397	0.999145
4	E	R_E31#5	Control - Mycotoxin	-4.56667	34.88072	52.97501	-119.739	110.6056	-0.13092	1
5	E	R_E5#44	Control - Mycotoxin	-26.0833	31.81817	16.25308	-141.696	89.52949	-0.81976	0.997049
6	E	R_E5#53	Control - Mycotoxin	21.16667	54.38265	45.50436	-159.659	201.9923	0.389217	0.999996
7	E	R_E6#52	Control - Mycotoxin	-80.6	50.00717	54.26011	-245.551	84.3515	-1.61177	0.836625
8	E	R_E8#57	Control - Mycotoxin	-37.5	40.51498	49.5493	-171.671	96.67061	-0.92558	0.994817
9	G	R_G1#3	Control - Mycotoxin	-28.5	54.38265	45.50436	-209.326	152.3256	-0.52406	0.999943
10	G	R_G1#31	Control - Mycotoxin	-89.1667	52.32999	52.43385	-262.03	83.69712	-1.70393	0.78823

Table S27

	Location	Isofemale_	contrast	estimate	SE	df	asymp.LCL	asymp.UCL	z.ratio	p.value
1	E	N_E1#70	Control - Mycotoxin	2.133153	0.441225	Inf	0.5813335	3.68497206	4.834618	0.000215
2	E	N_E10#72	Control - Mycotoxin	1.436921	0.419841	Inf	-0.0396903	2.9135328	3.422537	0.067492
3	E	N_E10#73	Control - Mycotoxin	15.9117	403.6702	Inf	-1403.8258	1435.6492	0.039418	1
4	E	N_E14#25	Control - Mycotoxin	2.327712	0.482834	Inf	0.62954904	4.02587555	4.820935	0.00023
5	E	N_E14#86	Control - Mycotoxin	2.778266	0.510759	Inf	0.98188779	4.57464371	5.439481	8.96E-06
6	E	N_E15#83	Control - Mycotoxin	2.463426	0.782467	Inf	-0.2885675	5.21541972	3.148281	0.148509
7	E	N_E17#17	Control - Mycotoxin	4.890601	1.060525	Inf	1.1606592	8.6205436	4.611493	0.00063
8	E	N_E20#5	Control - Mycotoxin	-0.58654	0.384442	Inf	-1.9386523	0.76556836	-1.5257	0.991837
9	E	N_E27#71	Control - Mycotoxin	2.324925	0.46695	Inf	0.68262711	3.96722375	4.978957	0.000105
10	G	N_G1#10	Control - Mycotoxin	2.224935	0.538556	Inf	0.33079516	4.11907422	4.1313	0.005195
11	G	N_G1#13	Control - Mycotoxin	1.671378	0.676257	Inf	-0.707066	4.04982165	2.471514	0.580013
12	G	N_G1#17	Control - Mycotoxin	2.009871	0.539626	Inf	0.11196563	3.90777676	3.72456	0.024687
13	G	N_G1#27	Control - Mycotoxin	2.547758	0.584848	Inf	0.49080445	4.60471214	4.356273	0.002001
14	G	N_G1#34	Control - Mycotoxin	3.403193	1.056718	Inf	-0.3133602	7.11974626	3.220532	0.12213
15	G	N_G1#41	Control - Mycotoxin	2.183835	0.586212	Inf	0.12208412	4.24558633	3.725332	0.024619
16	G	N_G1#5	Control - Mycotoxin	2.458114	0.782086	Inf	-0.292541	5.20876824	3.143021	0.150586
17	G	N_G1#7	Control - Mycotoxin	1.050478	0.455147	Inf	-0.5503074	2.65126432	2.307997	0.704432
18	G	N_G1#71	Control - Mycotoxin	3.326328	1.057949	Inf	-0.3945544	7.0472107	3.14413	0.150146
19	G	N_G1#9	Control - Mycotoxin	2.12805	0.664387	Inf	-0.2086476	4.46474812	3.203028	0.128161

Table S28

	Location	Isofemale_	contrast	estimate	SE	df	asymp.LCL	asymp.UCL	z.ratio	p.value
1	E	N_E1#70	Control - Mycotoxin	0.307722	0.784603	Inf	-2.2563624	2.87180617	0.392201	1
2	E	N_E14#25	Control - Mycotoxin	0.800727	0.582609	Inf	-1.103242	2.70469664	1.374381	0.968446
3	E	N_E14#86	Control - Mycotoxin	1.648582	0.802381	Inf	-0.9736005	4.27076543	2.054614	0.65542
4	E	N_E15#83	Control - Mycotoxin	2.400214	0.777367	Inf	-0.1402247	4.94065217	3.08762	0.08515
5	E	N_E27#71	Control - Mycotoxin	1.231484	0.491526	Inf	-0.3748262	2.83779448	2.505428	0.33625
6	G	N_G1#10	Control - Mycotoxin	0.754046	0.730232	Inf	-1.6323569	3.14044794	1.03261	0.99698
7	G	N_G1#17	Control - Mycotoxin	-1.13682	1.164193	Inf	-4.9414089	2.66776484	-0.97649	0.998171
8	G	N_G1#27	Control - Mycotoxin	1.794449	0.79973	Inf	-0.8190707	4.40796898	2.243819	0.517379
9	G	N_G1#41	Control - Mycotoxin	2.277935	0.652797	Inf	0.14459293	4.41127793	3.489502	0.024414
10	G	N_G1#7	Control - Mycotoxin	0.32959	0.57437	Inf	-1.5474529	2.20663375	0.573829	0.99999
11	G	N_G1#71	Control - Mycotoxin	2.223722	1.07294	Inf	-1.2826493	5.73009259	2.072551	0.642611
12	G	N_G1#9	Control - Mycotoxin	1.666162	0.807157	Inf	-0.9716291	4.30395356	2.064236	0.648562

Table S29

	Location	Isofemale_line	contrast	estimate	SE	df	lower.CL	upper.CL	t.ratio	p.value
1	E	N_E1#70	Control - M	-0.92702	0.265561	529.7057	-1.86577	0.011727	-3.49079	0.057455
2	E	N_E10#72	Control - M	-0.33776	0.242177	525.1861	-1.19388	0.518361	-1.39469	0.997025
3	E	N_E10#73	Control - M	NA	NA	NA	NA	NA	NA	NA
4	E	N_E14#25	Control - M	0.043914	0.298543	526.2502	-1.01146	1.099286	0.147095	1
5	E	N_E14#86	Control - M	-2.97493	0.352664	541.9148	-4.22144	-1.72842	-8.43559	3.72E-10
6	E	N_E15#83	Control - M	-0.44653	0.550808	535.409	-2.39351	1.500448	-0.81068	0.999999
7	E	N_E17#17	Control - M	-2.43128	0.723674	524.303	-4.98957	0.12701	-3.35963	0.0854
8	E	N_E20#5	Control - M	0.368974	0.215136	521.2126	-0.39159	1.129534	1.715073	0.970598
9	E	N_E27#71	Control - M	-0.76816	0.27541	522.1012	-1.7418	0.20547	-2.78916	0.344894
10	G	N_G1#10	Control - M	-0.57136	0.367554	537.2194	-1.87055	0.727835	-1.55449	0.989496
11	G	N_G1#13	Control - M	-0.91679	0.520419	558.4716	-2.75597	0.922385	-1.76164	0.961967
12	G	N_G1#17	Control - M	-1.4402	0.35189	525.7749	-2.68416	-0.19624	-4.09277	0.006887
13	G	N_G1#27	Control - M	-0.37196	0.379748	525.4496	-1.7144	0.97049	-0.97948	0.999976
14	G	N_G1#34	Control - M	0.614408	0.761408	539.5861	-2.07689	3.305701	0.806936	0.999999
15	G	N_G1#41	Control - M	-0.05598	0.458842	557.8086	-1.67755	1.56559	-0.12201	1
16	G	N_G1#5	Control - M	0.164788	0.657941	535.7194	-2.16087	2.490446	0.25046	1
17	G	N_G1#7	Control - M	-1.19957	0.302214	537.3101	-2.26781	-0.13134	-3.96929	0.011032
18	G	N_G1#71	Control - M	-0.44572	0.752717	535.1466	-3.1064	2.214961	-0.59215	1
19	G	N_G1#9	Control - M	-1.11536	0.470474	540.0789	-2.7783	0.547584	-2.37071	0.657655

Table S30

	contrast	estimate	SE	df	lower.CL	upper.CL	t.ratio	p.value
1	Control N_G1#27 - Mycotoxin N_G1#27	-2.39343	1.180519	8.90939	-6.08639	1.299521	-2.02744	0.247941
2	Control N_G1#27 - Control N_G1#7	-1.28597	1.254204	3.09606	-7.20946	4.637514	-1.02533	0.749381
3	Control N_G1#27 - Mycotoxin N_G1#7	0.824544	1.349574	2.85121	-5.92989	7.578976	0.610966	0.922046
4	Mycotoxin N_G1#27 - Control N_G1#7	1.107463	1.107616	5.353931	-2.87671	5.091636	0.999861	0.756227
5	Mycotoxin N_G1#27 - Mycotoxin N_G1#7	3.217978	1.214551	4.755943	-1.35381	7.789763	2.649519	0.153252
6	Control N_G1#7 - Mycotoxin N_G1#7	2.110515	0.927366	8.565551	-0.81453	5.035561	2.275817	0.178309

Table S31

	contrast	estimate	SE	df	lower.CL	upper.CL	t.ratio	p.value
1	Control N_	51.16333	32.38361	8.979657	-49.9783	152.3049	1.579914	0.435016
2	Control N_	4.078333	34.70385	4.939261	-124.587	132.744	0.117518	0.999339
3	Control N_	-14.3317	37.26351	3.479253	-177.909	149.246	-0.3846	0.977833
4	Mycotoxin	-47.085	33.45682	13.69698	-144.603	50.43274	-1.40734	0.515761
5	Mycotoxin	-65.495	36.10499	9.670568	-176.638	45.64752	-1.81402	0.323725
6	Control N_	-18.41	29.9387	8.625954	-112.7	75.87966	-0.61492	0.924655

Table S32

	Location	Isofemale_line	contrast	estimate	SE	df	lower.CL	upper.CL	t.ratio	p.value
1	E	N_E1#70	Control - Mycotoxin	-3.28614	1.365625	40.81755	-7.64672	1.07443	-2.40633	0.265655
2	E	N_E10#72	Control - Mycotoxin	-0.65584	0.879142	35.62319	-3.48445	2.172762	-0.74601	0.994777
3	E	N_E14#25	Control - Mycotoxin	-3.34692	1.120918	44.50741	-6.91068	0.216844	-2.98587	0.07958
4	E	N_E15#83	Control - Mycotoxin	-1.82789	0.997886	34.98487	-5.04206	1.386272	-1.83176	0.603848
5	E	N_E27#71	Control - Mycotoxin	-2.10118	0.9987	39.10639	-5.29741	1.095053	-2.10391	0.430281
6	G	N_G1#10	Control - Mycotoxin	-1.0917	1.802283	10.33073	-7.80533	5.621931	-0.60573	0.997943
7	G	N_G1#7	Control - Mycotoxin	2.304958	1.137242	36.43428	-1.34921	5.959128	2.026797	0.478889
8	G	N_G1#9	Control - Mycotoxin	-1.19687	1.048696	34.66894	-4.57656	2.18282	-1.14129	0.942669

Table S33

	Location	Isofemale_line	contrast	estimate	SE	df	lower.CL	upper.CL	t.ratio	p.value
1	E	N_E1#70	Control - Mycotoxin	-75.5805	37.85416	20.07734	-203.142	51.98081	-1.99662	0.507596
2	E	N_E10#72	Control - Mycotoxin	23.61695	32.4279	42.07498	-79.7673	127.0012	0.728291	0.995596
3	E	N_E14#25	Control - Mycotoxin	18.40789	33.31414	26.46104	-91.0193	127.8351	0.552555	0.999164
4	E	N_E15#83	Control - Mycotoxin	108.3774	37.44437	43.25992	-10.8347	227.5895	2.894357	0.09882
5	E	N_E27#71	Control - Mycotoxin	38.80799	35.45947	44.58381	-73.92	151.536	1.094432	0.954692
6	G	N_G1#10	Control - Mycotoxin	-30.7385	37.4294	7.836716	-179.681	118.2042	-0.82124	0.986178
7	G	N_G1#7	Control - Mycotoxin	-141.458	41.38288	41.23473	-273.528	-9.3879	-3.41827	0.028246
8	G	N_G1#9	Control - Mycotoxin	31.10932	38.9279	38.7244	-93.542	155.7606	0.799152	0.992179

Table S34

	contrast	estimate	SE	df	lower.CL	upper.CL	t.ratio	p.value
1	Control N_G1#27 - Mycotoxin N_G1#27	38.5	30.93562	7.999836	-60.5672	137.5672	1.24452	0.618687
2	Control N_G1#27 - Control N_G1#7	14.25	28.91258	2.009755	-184.994	213.4938	0.492865	0.953751
3	Control N_G1#27 - Mycotoxin N_G1#7	-0.33333	31.66708	2.01605	-217.799	217.132	-0.01053	0.999999
4	Mycotoxin N_G1#27 - Control N_G1#7	-24.25	26.96134	6.555466	-115.129	66.62913	-0.89944	0.805908
5	Mycotoxin N_G1#27 - Mycotoxin N_G1#7	-38.8333	29.89617	5.862916	-143.093	65.42658	-1.29894	0.596157
6	Control N_G1#7 - Mycotoxin N_G1#7	-14.5833	27.40092	7.788106	-102.883	73.71644	-0.53222	0.948687

Table S35

	contrast	estimate	SE	df	lower.CL	upper.CL	t.ratio	p.value
1	Control N_E27#71 - Mycotoxin N_E27#71	-72	36.94273	5.428923	-204.23	60.22974	-1.94896	0.310291
2	Control N_E27#71 - Control N_G1#10	-40.75	34.76898	1.944768	NA	NA	-1.17202	NA
3	Control N_E27#71 - Mycotoxin N_G1#10	-21	37.14542	2.257753	-247.702	205.7017	-0.56535	0.934891
4	Mycotoxin N_E27#71 - Control N_G1#10	31.25	36.30341	3.36877	-131.21	193.7102	0.860801	0.825182
5	Mycotoxin N_E27#71 - Mycotoxin N_G1#10	51	38.58547	3.346232	-122.381	224.3812	1.321741	0.605016
6	Control N_G1#10 - Mycotoxin N_G1#10	19.75	36.096	1.635731	NA	NA	0.547152	NA

Table S36

	Location	Isofemale_line	contrast	estimate	SE	df	asymp.LCL	asymp.UCL	z.ratio	p.value
1	E	T_E14#3	Control - Mycotoxin	4.162132	0.805367	Inf	1.32959591	6.99466786	5.167991	3.92E-05
2	E	T_E15#11	Control - Mycotoxin	2.301377	0.512414	Inf	0.499178428	4.103576	4.491242	0.001096
3	E	T_E17#8	Control - Mycotoxin	1.223743	0.44725	Inf	-0.34926909	2.79675486	2.736148	0.378307
4	E	T_E22#9	Control - Mycotoxin	-0.00062	0.430736	Inf	-1.51555383	1.51430397	-0.00145	1
5	E	T_E25#4	Control - Mycotoxin	3.893939	0.641821	Inf	1.636607129	6.15127174	6.067015	2.22E-07
6	E	T_E25#5	Control - Mycotoxin	3.528185	0.812317	Inf	0.671206487	6.385164	4.343359	0.002117
7	E	T_E27#10	Control - Mycotoxin	2.928401	0.57033	Inf	0.922507032	4.934294	5.134569	4.67E-05
8	E	T_E3#7	Control - Mycotoxin	2.088245	0.485969	Inf	0.379055008	3.7974349	4.297071	0.002588
9	E	T_E30#6	Control - Mycotoxin	2.457498	0.54666	Inf	0.534853922	4.38014127	4.495476	0.001075
10	E	T_E31#1	Control - Mycotoxin	1.289517	0.46141	Inf	-0.33329645	2.91233073	2.79473	0.337763
11	G	T_G1#1	Control - Mycotoxin	2.696438	0.581089	Inf	0.65270484	4.74017042	4.640317	0.00055
12	G	T_G1#10	Control - Mycotoxin	2.336012	0.571057	Inf	0.327564172	4.34445981	4.090684	0.00613
13	G	T_G1#22	Control - Mycotoxin	1.645283	0.569107	Inf	-0.35630678	3.64687366	2.890992	0.276348
14	G	T_G1#3	Control - Mycotoxin	3.524187	0.659685	Inf	1.204026864	5.84434782	5.342227	1.53E-05
15	G	T_G1#4	Control - Mycotoxin	2.57898	0.51686	Inf	0.761146085	4.39681293	4.989709	9.90E-05
16	G	T_G1#5	Control - Mycotoxin	3.27099	0.606556	Inf	1.137689403	5.40429064	5.392728	1.16E-05
17	G	T_G1#6	Control - Mycotoxin	3.566687	0.61449	Inf	1.40548129	5.7278918	5.804306	1.09E-06
18	G	T_G1#7	Control - Mycotoxin	2.095645	0.487568	Inf	0.380831048	3.81045896	4.298155	0.002576
19	G	T_G1#8	Control - Mycotoxin	3.106206	0.568307	Inf	1.107428528	5.10498356	5.465718	7.74E-06

Table S37

	Location	Isofemale_line	contrast	estimate	SE	df	asymp.LCL	asymp.UCL	z.ratio	p.value
1	E	T_E15#11	Control - Mycotoxin	2.299671	0.512569	Inf	0.5114785	4.087863695	4.486557	0.001007
2	E	T_E17#8	Control - Mycotoxin	0.808874	0.456214	Inf	-0.7827123	2.400459849	1.773015	0.951348
3	E	T_E22#9	Control - Mycotoxin	0.095571	0.438019	Inf	-1.4325396	1.623681438	0.218189	1
4	E	T_E25#4	Control - Mycotoxin	2.914285	0.685411	Inf	0.5231023	5.305466663	4.25188	0.002829
5	E	T_E25#5	Control - Mycotoxin	2.004738	0.807333	Inf	-0.8117925	4.821268251	2.483161	0.543325
6	E	T_E27#10	Control - Mycotoxin	2.882076	0.562576	Inf	0.9194256	4.844727301	5.122998	4.45E-05
7	E	T_E3#7	Control - Mycotoxin	1.489113	0.508115	Inf	-0.2835405	3.261767029	2.930661	0.235337
8	E	T_E30#6	Control - Mycotoxin	1.409336	0.696906	Inf	-1.0219494	3.840620719	2.022275	0.858908
9	E	T_E31#1	Control - Mycotoxin	0.395419	0.446502	Inf	-1.1622856	1.953124474	0.885594	0.99999
10	G	T_G1#1	Control - Mycotoxin	1.98936	0.482904	Inf	0.3046592	3.674060218	4.119574	0.004919
11	G	T_G1#10	Control - Mycotoxin	2.103764	0.799018	Inf	-0.6837577	4.891285687	2.632937	0.428764
12	G	T_G1#22	Control - Mycotoxin	0.823103	0.460367	Inf	-0.7829711	2.429177098	1.787929	0.947579
13	G	T_G1#3	Control - Mycotoxin	2.356521	0.521093	Inf	0.5385933	4.174449033	4.522269	0.000855
14	G	T_G1#4	Control - Mycotoxin	4.493006	1.074879	Inf	0.7430927	8.242918689	4.180012	0.003831
15	G	T_G1#5	Control - Mycotoxin	0.971883	0.589877	Inf	-1.0860111	3.029776611	1.647603	0.975606
16	G	T_G1#6	Control - Mycotoxin	2.14849	0.607674	Inf	0.0285063	4.268474272	3.535595	0.042904
17	G	T_G1#7	Control - Mycotoxin	3.293916	0.691542	Inf	0.8813454	5.706486702	4.76315	0.000274
18	G	T_G1#8	Control - Mycotoxin	2.343898	0.557317	Inf	0.3995963	4.288199595	4.205686	0.00344

Tabls S38

	Location	Isofemale_line	contrast	estimate	SE	df	lower.CL	upper.CL	t.ratio	p.value
1	E	T_E14#3	Control - Mycotoxin	0.779213	0.713343	765.9551	-1.73848	3.2969102	1.092339	0.999887
2	E	T_E15#11	Control - Mycotoxin	-0.39423	0.372937	755.4274	-1.71055	0.9220967	-1.05708	0.99993
3	E	T_E17#8	Control - Mycotoxin	0.088585	0.296569	752.438	-0.9582	1.1353726	0.298699	1
4	E	T_E22#9	Control - Mycotoxin	-0.2867	0.308198	766.6896	-1.37446	0.8010636	-0.93024	0.999989
5	E	T_E25#4	Control - Mycotoxin	0.028617	0.441576	752.7958	-1.52999	1.5872273	0.064807	1
6	E	T_E25#5	Control - Mycotoxin	-0.87769	0.700745	760.6568	-3.35099	1.5956033	-1.25251	0.999257
7	E	T_E27#10	Control - Mycotoxin	-0.65808	0.314897	764.4387	-1.7695	0.4533335	-2.08983	0.842677
8	E	T_E3#7	Control - Mycotoxin	-0.05903	0.323917	753.1762	-1.20234	1.0842815	-0.18224	1
9	E	T_E30#6	Control - Mycotoxin	-1.4768	0.418791	753.7087	-2.95498	0.0013852	-3.52633	0.050532
10	E	T_E31#1	Control - Mycotoxin	2.348993	0.270353	761.9469	1.394783	3.3032021	8.688626	0
11	G	T_G1#1	Control - Mycotoxin	-0.7071	0.266812	754.7063	-1.64884	0.2346472	-2.65017	0.442951
12	G	T_G1#10	Control - Mycotoxin	0.466534	0.465504	789.2213	-1.17626	2.1093288	1.002214	0.999968
13	G	T_G1#22	Control - Mycotoxin	-0.31098	0.239533	758.4828	-1.15642	0.5344683	-1.29826	0.998812
14	G	T_G1#3	Control - Mycotoxin	-0.31156	0.342828	774.8308	-1.5215	0.8983838	-0.90879	0.999993
15	G	T_G1#4	Control - Mycotoxin	-1.61481	0.400907	779.7925	-3.0297	-0.199926	-4.0279	0.00853
16	G	T_G1#5	Control - Mycotoxin	0.270098	0.465136	762.9065	-1.37159	1.9117876	0.580687	1
17	G	T_G1#6	Control - Mycotoxin	-1.02271	0.477988	769.4519	-2.70971	0.6642897	-2.13962	0.814756
18	G	T_G1#7	Control - Mycotoxin	-1.73552	0.35534	771.6991	-2.98964	-0.481404	-4.88411	0.000203
19	G	T_G1#8	Control - Mycotoxin	-0.28944	0.386652	754.3956	-1.65418	1.0752932	-0.74859	1

Table S39

	Location	Isofemale_line	contrast	estimate	SE	df	lower.CL	upper.CL	t.ratio	p.value
1	E	T_E15#11	Control - Mycotoxin	-1.76414	0.551539	81.58143	-3.47997	-0.0483	-3.19857	0.039433
2	E	T_E17#8	Control - Mycotoxin	-0.48417	0.564984	84.05834	-2.24048	1.272137	-0.85697	0.988968
3	E	T_E22#9	Control - Mycotoxin	-0.22115	0.431415	80.03523	-1.56395	1.121657	-0.51261	0.999567
4	E	T_E27#10	Control - Mycotoxin	-1.11779	0.498973	85.63058	-2.66817	0.432599	-2.24018	0.33892
5	E	T_E31#1	Control - Mycotoxin	0.62747	0.495488	80.60114	-0.91448	2.169419	1.266367	0.908431
6	G	T_G1#1	Control - Mycotoxin	-0.18044	0.515277	79.41028	-1.78461	1.423721	-0.35019	0.999966
7	G	T_G1#22	Control - Mycotoxin	-0.32277	0.384842	82.30536	-1.51974	0.874196	-0.83871	0.990279
8	G	T_G1#3	Control - Mycotoxin	0.5	0.662167	80.78302	-1.56053	2.560526	0.755097	0.994845

Table S40

	Location	Isofemale_line	contrast	estimate	SE	df	lower.CL	upper.CL	t.ratio	p.value
1	E	T_E15#11	Control - Mycotoxin	35.51497	30.65765	88.40767	-59.6688	130.6987	1.158437	0.941346
2	E	T_E17#8	Control - Mycotoxin	74.45326	30.94947	90.82472	-21.575	170.4815	2.405639	0.251313
3	E	T_E22#9	Control - Mycotoxin	50.89729	24.2511	88.37383	-24.3966	126.1911	2.098762	0.423853
4	E	T_E27#10	Control - Mycotoxin	15.39107	27.11045	90.93028	-68.7234	99.50551	0.567717	0.999163
5	E	T_E31#1	Control - Mycotoxin	32.62132	27.71085	85.82341	-53.4758	118.7184	1.177204	0.936274
6	G	T_G1#1	Control - Mycotoxin	20.53706	29.00176	85.26671	-69.5855	110.6596	0.708131	0.99655
7	G	T_G1#22	Control - Mycotoxin	-16.0482	21.34864	90.84143	-82.2872	50.19088	-0.75172	0.995024
8	G	T_G1#3	Control - Mycotoxin	33.57036	36.96524	86.21219	-81.2671	148.4079	0.90816	0.984529

Tabls S41

	Location	Isofemale_line	contrast	estimate	SE	df	lower.CL	upper.CL	t.ratio	p.value
1	E	T_E15#11	Control - Mycotoxin	0.07855	0.772287	172.6186	-2.51621	2.67331	0.101711	1
2	E	T_E17#8	Control - Mycotoxin	-0.99457	0.57669	168.2997	-2.93285	0.943721	-1.72461	0.881869
3	E	T_E22#9	Control - Mycotoxin	0.169208	0.607777	165.7412	-1.87402	2.212434	0.278405	1
4	E	T_E27#10	Control - Mycotoxin	-1.26411	0.767347	153.8951	-3.84669	1.318472	-1.64738	0.911709
5	E	T_E3#7	Control - Mycotoxin	-0.64013	0.682959	172.9975	-2.93469	1.654429	-0.93729	0.999282
6	E	T_E30#6	Control - Mycotoxin	-3.35305	0.845872	172.1772	-6.19514	-0.51095	-3.96401	0.006874
7	E	T_E31#1	Control - Mycotoxin	1.796084	0.589458	162.5147	-0.18613	3.778302	3.047011	0.118702
8	G	T_G1#1	Control - Mycotoxin	-0.00993	0.520647	151.713	-1.76261	1.742763	-0.01906	1
9	G	T_G1#22	Control - Mycotoxin	-0.04206	0.400102	158.0071	-1.38809	1.303969	-0.10513	1
10	G	T_G1#3	Control - Mycotoxin	0.549329	0.616695	157.8421	-1.5254	2.624058	0.890762	0.999566
11	G	T_G1#5	Control - Mycotoxin	0.192993	0.902337	124.9937	-2.85501	3.241	0.213881	1
12	G	T_G1#7	Control - Mycotoxin	-1.96573	0.791736	156.1814	-4.62978	0.698316	-2.48282	0.393307
13	G	T_G1#8	Control - Mycotoxin	-3.26177	0.79135	155.7407	-5.92464	-0.5989	-4.12178	0.003999

Tabls S42

	Location	Isofemale_line	contrast	estimate	SE	df	lower.CL	upper.CL	t.ratio	p.value
1	E	T_E15#11	Control - Mycotoxin	11.86957	43.07943	165.523	-132.958	156.697	0.275528	1
2	E	T_E17#8	Control - Mycotoxin	59.04332	32.66408	172.6954	-50.7021	168.7887	1.807592	0.843372
3	E	T_E22#9	Control - Mycotoxin	11.77206	32.81965	114.8728	-99.2793	122.8234	0.358689	1
4	E	T_E27#10	Control - Mycotoxin	90.61312	44.47421	161.2473	-58.9616	240.1878	2.03743	0.706655
5	E	T_E3#7	Control - Mycotoxin	-15.5671	37.85112	162.483	-142.852	111.7184	-0.41127	1
6	E	T_E30#6	Control - Mycotoxin	73.03206	46.53928	162.8797	-83.4642	229.5283	1.569256	0.936715
7	E	T_E31#1	Control - Mycotoxin	-82.5416	33.73813	168.9112	-195.931	30.84813	-2.44654	0.417131
8	G	T_G1#1	Control - Mycotoxin	75.82716	30.23971	156.4321	-25.9215	177.5759	2.507536	0.377081
9	G	T_G1#22	Control - Mycotoxin	13.7724	23.0699	167.456	-63.7724	91.31723	0.596986	0.999994
10	G	T_G1#3	Control - Mycotoxin	41.23869	35.53564	165.1904	-78.231	160.7084	1.160488	0.994482
11	G	T_G1#5	Control - Mycotoxin	-46.1926	46.47743	94.9549	-204.155	111.7699	-0.99387	0.998612
12	G	T_G1#7	Control - Mycotoxin	135.7403	45.71305	162.0432	-17.9895	289.47	2.969399	0.143916
13	G	T_G1#8	Control - Mycotoxin	138.2403	45.72842	159.5406	-15.5774	292.0579	3.023071	0.126261

Table S43

	Location	Isofemale_line	contrast	estimate	SE	df	lower.CL	upper.CL	t.ratio	p.value
1	E	T_E15#11	Control - Mycotoxin	11.13072	21.48152	84.584	-55.636227	77.897674	0.518153	0.999537
2	E	T_E17#8	Control - Mycotoxin	5.08093	22.11227	85.25212	-63.6329	73.79476	0.229779	0.999998
3	E	T_E22#9	Control - Mycotoxin	37.04125	17.01151	84.76728	-15.829525	89.912028	2.177423	0.37573
4	E	T_E27#10	Control - Mycotoxin	-42.8668	20.51527	80.56919	-106.71054	20.976939	-2.08951	0.430468
5	E	T_E31#1	Control - Mycotoxin	-34.2381	21.31149	79.69542	-100.57883	32.102715	-1.60655	0.745004
6	G	T_G1#1	Control - Mycotoxin	-55.3599	20.33825	81.55002	-118.6327	7.9128017	-2.72196	0.130851
7	G	T_G1#22	Control - Mycotoxin	-21.1626	14.96179	86.94099	-67.633795	25.308609	-1.41444	0.848215
8	G	T_G1#3	Control - Mycotoxin	38.00093	25.90716	82.35341	-42.576305	118.57816	1.466812	0.822521

Table S44

	Location	Isofemale_line	contrast	estimate	SE	df	lower.CL	upper.CL	t.ratio	p.value
1	E	T_E15#11	Control - Mycotoxin	238.2143	108.0314	32.26703	-101.167	577.5952	2.205047	0.320733
2	E	T_E17#8	Control - Mycotoxin	107.2571	82.7252	22.23511	-160.204	374.718	1.296547	0.846372
3	E	T_E22#9	Control - Mycotoxin	77.16667	87.8579	18.05647	-213.047	367.3806	0.878312	0.971436
4	E	T_E3#7	Control - Mycotoxin	-0.5	102.598	36.84642	-320.274	319.2739	-0.00487	1
5	E	T_E30#6	Control - Mycotoxin	25.16667	119.5505	52.28851	-341.37	391.7037	0.210511	0.999992
6	G	T_G1#22	Control - Mycotoxin	63.64545	63.27213	34.40283	-134.341	261.6315	1.0059	0.949186
7	G	T_G1#5	Control - Mycotoxin	-28	118.1664	30.35013	-400.716	344.7155	-0.23695	0.999983

Table S45

	contrast	Treatment	estimate	SE	df	asymp.LCL	asymp.UCL	z.ratio	p.value
1	E - G	Control	0.166308	0.1353	Inf	-0.09887511	0.431490674	1.229179	0.219005
2	E - G	Mycotoxin	0.41761	0.143444	Inf	0.13646523	0.698754348	2.911314	0.003599

Table S46

	contrast	Treatment	estimate	SE	df	asymp.LCL	asymp.UCL	z.ratio	p.value
1	E - G	Control	-0.07884	0.133108	Inf	-0.33972688	0.18204503	-0.59231	0.553643
2	E - G	Mycotoxin	0.22906	0.149858	Inf	-0.06465529	0.52277619	1.528519	0.126384
